# Up‐regulation of secretory leukocyte protease inhibitor in human samples might have a potential role of predicting prostate cancer recurrence and progression after surgery and hormonal therapy

**DOI:** 10.1002/cam4.5134

**Published:** 2022-08-13

**Authors:** Yu Miyazaki, Takayuki Goto, Xin Li, Kenji Nakayama, Kosuke Okasho, Masashi Takeda, Kei Mizuno, Hiroko Kimura, Masayuki Uegaki, Takayuki Sumiyoshi, Yuki Teramoto, Shusuke Akamatsu, Takashi Kobayashi, Osamu Ogawa, Takahiro Inoue

**Affiliations:** ^1^ Department of Urology, Graduate School of Medicine Kyoto University Kyoto Japan; ^2^ Department of Diagnostic Pathology Kyoto University Hospital Kyoto Japan; ^3^ Department of Nephro‐Urologic Surgery and Andrology Mie University Graduate School of Medicine Tsu Japan

**Keywords:** castration‐resistant, prostate cancer, proteomic analysis, secretory leukocyte protease inhibitor, tumor marker

## Abstract

Using new castration‐resistant prostate cancer (CRPC) cell lines developed from LNCaP cells as a model for CRPC, we searched for novel biomarkers by analyzing the proteins secreted in culture supernatants. The results showed that the levels of secretory leukocyte protease inhibitor (SLPI) in these cell lines were 4.7–6.7 times higher than those secreted in parental LNCaP. Patients with localized prostate cancer (PC) and who expressed SLPI had a significantly lower prostate‐specific antigen (PSA) progression‐free survival rate than those who did not. Multivariate analysis revealed that SLPI expression was an independent risk factor for PSA recurrence. By contrast, when immunostaining of SLPI was performed on consecutive prostate tissue samples obtained from 11 patients, both in hormone naive (HN) and castration resistant (CR) conditions, only one patient expressed SLPI in the HNPC state; however, four of the 11 patients expressed SLPI in the CRPC state. In addition, two of these four patients were resistant to enzalutamide, and there was a discrepancy between their serum PSA levels and radiographic progression of the disease. These results suggest that SLPI can be a predictor of prognosis in patients with localized PC and disease progression in CRPC patients.

## Graphical abstract

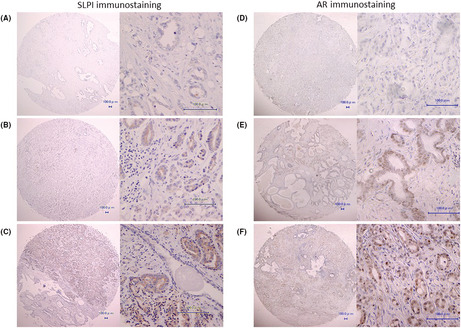

## 1 INTRODUCTION

Prostate cancer (PC) was the most prevalent cancer in the United States, with an estimated 191,930 new cases and 33,330 deaths in 2020.^1^ In Japan, it was the fourth most common type among men in 2019, and despite substantial advances in diagnosis and treatment, more than 12,000 men die from PC each year.^2^ A standard treatment for metastatic advanced PC is androgen deprivation therapy (ADT), and in most cases, the treatment is effective, but within 2–3 years it becomes refractory, termed as castration‐resistant PC (CRPC), the prognosis of which is dismal. However, during the past 15 years, treatment against CRPC has improved, with the introduction of taxanes and androgen receptor signaling inhibitors (ARSIs) in the clinical field.^3^, ^4^, ^5^, ^6^ Nonetheless, we are now confronted with the challenge of choosing the best drugs for individual patients to introduce a personalized treatment approach. Currently, serum prostate‐specific antigen (PSA) is the only clinically applicable biomarker for monitoring efficacy and disease relapse. However, PSA is not ideal for monitoring therapeutic outcomes. Recent metastatic hormone naive PC (HNPC) trials using ARSIs revealed that radiographic progression may occur earlier than PSA progression.^7^ Hence, new reliable markers are needed to monitor and predict treatment outcomes. Several clinical and preclinical biomarkers have been investigated with the aim to improve treatment strategies in CRPC.^8^ AR‐V7 detected in circulating tumor cells (CTCs) has been reported as a biomarker for enzalutamide resistance,^9^ and nuclear‐localized AR‐V7 in CTCs may improve clinical decision‐making in selecting between ARSIs and taxanes.^10^ We also reported androgen receptor gene aberrations in circulating cell‐free DNA as a biomarker for the treatment of CRPC.^11^ However, currently, this test is not widely available in clinical practice due to its high cost and lack of definitive evidence. Therefore, we aimed to evaluate molecular biomarkers for disease monitoring in CRPC patients, especially those with resistance to enzalutamide.

Herein, we performed proteomic analysis of tissue culture supernatants obtained from enzalutamide‐resistant derivatives of LNCaP cells, AILNCaP14 and AILNCaP15 cells, by long‐term subculture in androgen‐depleted conditions. We found that secretory leukocyte protease inhibitor (SLPI) was upregulated in both AILNCaP14 and AILNCaP15 cells than in LNCaP cells. Therefore, we evaluated its potential properties in clinical settings by assessing SLPI expression in human PC tissues and serum of PC patients.

## 2 MATERIALS AND METHODS

### 2.1 Cell culture

#### 2.1.1 Cell culture and collection of culture supernatants

PC cell lines LNCaP, DU145, and 22Rv1 were obtained from the American Type Culture Collection (Manassas, VA, USA). AILNCaP14 and AILNCaP15 cells are kindly provided by Dr. Eijiro Nakamura (National Cancer Center Hospital, Tokyo, Japan). LNCaP cells were routinely cultured in RPMI 1640 (Invitrogen, Waltham, MA, USA) supplemented with 10% fetal bovine serum (FBS). DU145, 22Rv1, AILNCaP14, and AILNCaP15 cells were cultured in phenol red‐free RPMI 1640 supplemented with charcoal‐stripped FBS (CSFBS; Hyclone; GE Healthcare, Chicago, IL, USA) for androgen‐depleted conditions. Because FBS influences subsequent protein analysis, the culture supernatant was discarded at 80% cell confluency, the cells washed twice with PBS, changed to phenol red‐free RPMI 1640 without FBS (or CSFBS), and cultured for 48 h. Next, the culture supernatants were collected and used as samples.

#### 2.1.2 SLPI knockdown in AILNCaP14 cells and cell proliferation assay

AILNCaP14 cells were transfected with the pLKO.1‐TRC cloning vector (Plasmid # 10878, Addgene, Watertown, MA, USA) including the shSLPI (#1: 5’‐GCGTGACTTGAAGTGTTGCAT‐3′, #4: 5’‐GAGTCTGTCCTCCTAAGAAAT‐3'), or with the plasmid scramble shRNA (Plasmid # 1864, Addgene) including shScramble. And these transfected cells were cultured in androgen‐depleted conditions mentioned as above. AILNCaP14 parent cells and transfected cells with totally 500 cells were seeded into 96‐well plates. Proliferative activities were measured using the MTT assay kit (Cell Counting Kit‐8, Dojindo Laboratories, Kumamoto, Japan). All experiments were performed in triplicate.

### 2.2 Proteomic analyses

#### 2.2.1 Sample preparation

A denaturing agent (9 M urea, 2% CHAPS, 50 mM Tris pH 9) was added to 13.5 ml of the collected culture supernatant, and the mixture was sufficiently reacted. The sample was applied to an Amicon Ultra‐15 3,000 NMWL (Merck KGaA, Darmstadt, Germany) filter unit and concentrated using a swing rotor. When the sample was concentrated to about 0.5 ml, 5 ml of 100 mM ammonium bicarbonate was added and concentrated again. By repeating this procedure three times, the culture supernatant was desalted and concentrated. The obtained concentrates were collected in PROTEOSAVE™ microtubes (Sumitomo Bakelite Co., Ltd., Tokyo, Japan).

The samples were dried using a centrifugal concentrator (EYELA CVE‐3000, Tokyo Rikakikai, Tokyo, Japan) connected with a diaphragm vacuum pump (MD1C model, Vacuubrand, Germany) and a cold trap (EYELA Uni Trap UT‐2000, Tokyo Rikakikai). Details of experimental procedures were presented in supporting material and methods (Doc S1).

#### 2.2.2 Two‐dimensional polyacrylamide gel electrophoresis (2D‐PAGE)

The dried culture supernatant was dissolved in sample buffer (8 M urea, 2%CHAPS, 50 mM dithiothreitol [DTT], 0.2% Bio‐Lyte 3/10 carrier ampholyte, 0.001%BPB, #1632108 Bio‐Rad Laboratories, Inc., Hercules, CA, USA) and adjusted to 0.4 mg/ml. A total of 125 μl (50 μg) of the prepared samples was applied to immobilized pH gradient (IPG) strips (ReadyStrip, pH 3‐10 non‐linear, # 1632002, Bio‐Rad Laboratories, Inc.), and isoelectric focusing (IEF) was performed using the PROTEAN® i12 IEF System (Bio‐Rad Laboratories, Inc.) according to the manufacturer's instructions. Later, the IPG strips were reduced with DTT, carbamidemethylated with iodoacetamide, applied to precast gels (Any kD Mini‐PROTEAN TGX Precast Gel # 4569031, Bio‐Rad Laboratories, Inc.), and sodium dodecyl sulfate polyacrylamide gel electrophoresis (SDS‐PAGE) was performed. The electrophoresed gels were stained with Flamingo Fluorescent Gel Stain (# 1610491, Bio‐Rad Laboratories, Inc.) or Coomassie Brilliant Blue (CBB) (Expedeon, Cambridge, UK). Image analyses of the stained gels were performed using a Gel Imaging system (Gel Doc EZ system, Bio‐Rad Laboratories, Inc.) and PDQuest 2‐D analysis software (Bio‐Rad Laboratories, Inc.).

#### 2.2.3 Matrix assisted laser desorption/ionization‐time of flight mass spectrometry (MALDI‐TOF/MS)

The spots that exhibited differences between LNCaP and AILNCaP14 or AILNCaP15 cells in image analysis were excised, destained with acetonitrile, reduced with DTT, carbamidemethylated with iodoacetamide, and digested with trypsin (APRO SCIENCE, Tokushima, Japan). The collected solutions were dried using a centrifugal concentrator CC‐105 (TOMY SEIKO Co., LTD., Tokyo, Japan), mixed with 2, 5‐dihydroxybenzoic acid as a matrix, and analyzed using a MALDI‐TOF mass spectrometer (AXIMA Resonance and/or Performance, Shimadzu Corporation, Kyoto, Japan). The data were analyzed using Mascot (Matrix Science Ltd., London, UK). Details of experimental procedures were presented in supporting material and methods (Doc S1).

#### 2.2.4 Isobaric Tag for Relative and Absolute Quantitation (iTRAQ)/Tandem Mass Tag (TMT) analysis

The concentrated and desalted culture supernatant samples were labeled with TMTsixplex™ Isobaric Label Reagent (Thermo Fisher Scientific Inc., Waltham, MA, USA) and analyzed using the Easy‐nLC 1000‐Orbitrap Q Exactive Plus system (Thermo Fisher Scientific Inc.) and a nano‐HPLC capillary column (Nikkyo Technos, Co., Ltd., Tokyo, Japan) as an analytical column. The data on the proteins present in the culture supernatants of LNCaP, AILNCaP14, and AILNCaP15 were subjected to relative quantification analysis using Proteome Discoverer (Thermo Fisher Scientific Inc.).

### 2.3 Human serum and prostate tissue samples

#### 2.3.1 Serum sample collection

Sixty‐nine male patients were recruited at the Kyoto University Hospital between April 2016 and August 2019. Details of the 69 patients are as follows: 12 preoperative benign prostatic hyperplasia (BPH) patients (all patients pathologically confirmed that the excised tissue did not contain cancer tissue), 10 patients with organ‐confined PC (localized PC) before prostatectomy, 10 metastatic hormone‐naive PC (mHNPC) patients before the start of treatment, and 37 CRPC patients. Consecutive blood samples were collected from 11 of the 37 patients with CRPC as their treatment progressed. The collected sera were stored at −80°C until used.

#### 2.3.2 Localized or local advanced prostate cancer specimens

Tissue‐microarrays (TMAs) were prepared using radical prostatectomy specimens from 175 patients, as previously reported.^12^ PSA failure was defined as two consecutive measurements of PSA levels ≥0.2 ng/ml, and the date of PSA failure as the date at which the first measurement of PSA level was ≥0.2. When PSA levels after surgery did not decline below 0.2 ng/mL, we defined the date of PSA failure as the date at which surgery was performed.

#### 2.3.3 Evaluation of prostate samples in hormone naive and castration‐ resistant states

Consecutive prostate tissue samples obtained from 11 patients, both in hormone naive and castration‐resistant states, were evaluated. HNPC specimens consisted of samples obtained by performing needle biopsy or transurethral resection of the prostate (TUR‐P). CRPC specimens were collected from patients undergoing TUR‐P for urinary retention or gross hematuria, penectomy for pain control, and spinal laminectomy for spinal cord compression due to bone metastases.

### 2.4 Immunohistochemistry

Immunohistochemistry of SLPI (at 1:1000) and androgen receptor (AR) (at 1:400) was performed. We used paraffin‐embedded blocks prepared using 22Rv1 and AILNCaP15 cells as negative and positive controls for staining of SLPI, respectively. The Ventana Discovery Ultra system (Roche Diagnostics, Rotkreuz, Switzerland) was employed as an automatic immunohistochemical staining apparatus. All the specimens were evaluated by a urological pathologist (T.Y.). Similar to our previously reported method, we scored the intensity and proportion of staining, and the sum of these evaluation scores as the total score (TS) to assess the degree of immunostaining with SLPI and AR.^12^ Details of immunostaining score were presented in supporting material and methods (Doc S1).

### 2.5 Data acquisition

RNA‐seq data sets of CRPC patients available online were obtained through TCGA.^13^

### 2.6 Enzyme‐Linked Immunosorbent Assay (ELISA)

Serum SLPI concentration was measured by ELISA using the Human SLPI Quantikine ELISA kit (R&D Systems, Minneapolis, MN, USA) according to the manufacturer's instructions.

### 2.7 Statistical analyses

Data were analyzed using JMP13 software (SAS Institute Inc., Cary, North Carolina, USA), and P‐values were calculated using Kruskal–Wallis test, Pearson's chi‐squared test, and Wilcoxon signed‐rank test. PSA progression‐free survival was estimated using Kaplan–Meier analysis and groups were compared employing the log‐rank test. Cox proportional hazard analysis was used to examine factors associated with PSA progression‐free survival. The correlation coefficient between SLPI and AR mRNA expression was calculated using Spearman's rank correlation coefficient method. Student's *t* test was used for ELISA and proliferation assay results to compare between each group. Statistical significance was set at *p* < 0.05.

## 3 RESULTS

### 3.1 Proteomics analysis of prostate cancer cell culture supernatants

**FIGURE 1:**
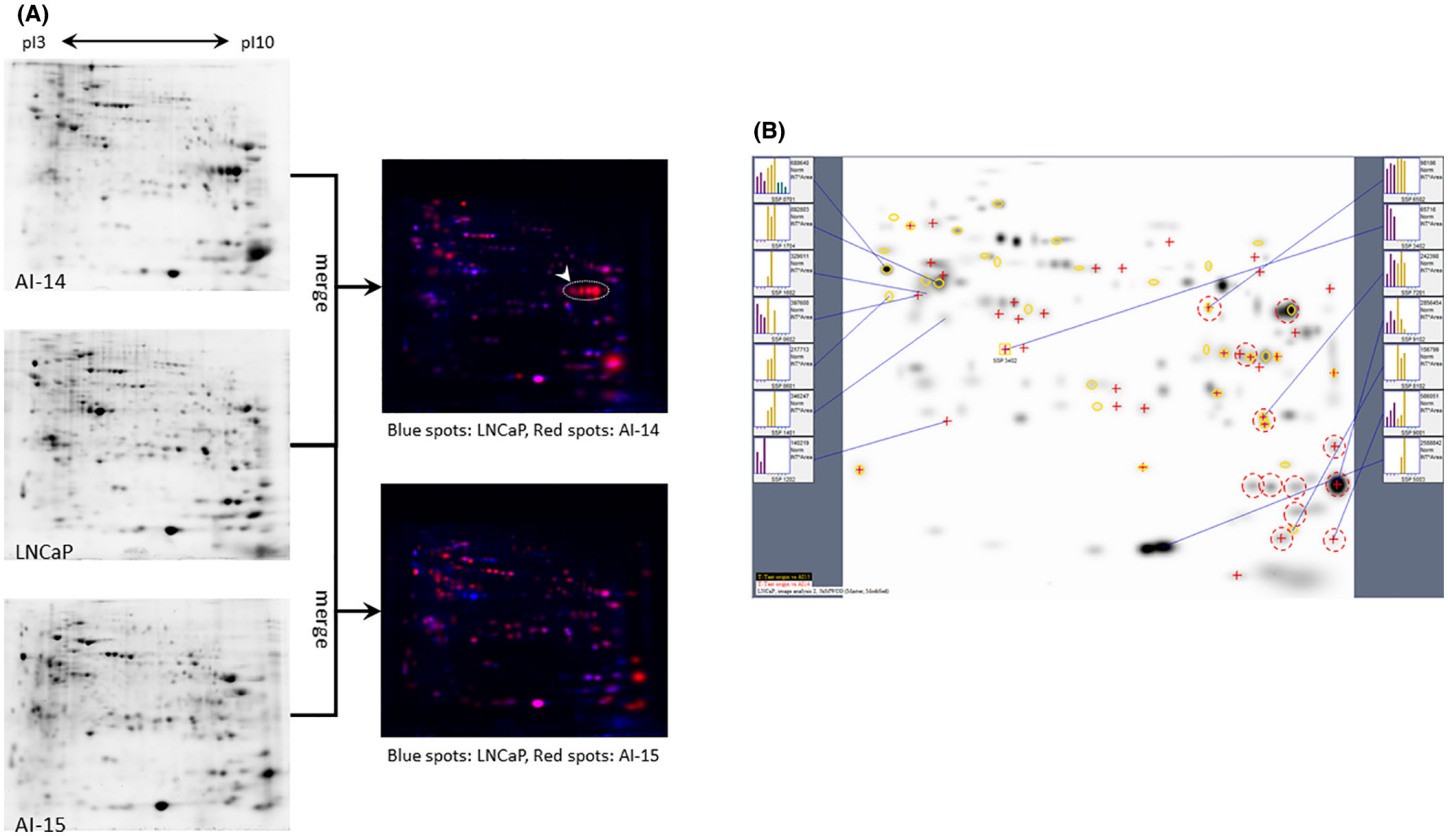
Analyses of 2D‐PAGE images of cell line culture supernatants. (A) Representative gel image and its merged image. Red spots represent the spots of molecules whose expression was abundant in AILNCaP14 (AI‐14) and AILNCaP15 (AI‐15) than in LNCaP cells. The spot represented by a white arrowhead was identified as PSA by subsequent analysis using MALDI‐TOF/MS. 2D‐PAGE: Two‐dimensional polyacrylamide gel electrophoresis, PSA: prostate specific antigen, MALDI‐TOF/MS: Matrix assisted laser desorption/ionization‐time of flight mass spectrometry. (B) Quantitative comparative analysis using image analysis software. Three gel images were created for each cell line using 2D‐PAGE, and the difference in signal intensity at each spot was calculated employing Student's *t* test. Spots with significantly enhanced signal intensity compared to those in LNCaP were used for subsequent mass spectrometry. The bar graphs displayed at both ends of the image show the signal intensity in each gel; purple: AILNCaP14 (AI‐14), yellow: AILNCaP15 (AI‐15), and green: LNCaP. Each spot surrounded by a red dashed circle was cut out and analyzed by mass spectrometry

In 2D‐PAGE image analyses of culture supernatants, unique multiple spots were found in AILNCaP14 and AILNCaP15, as shown in Figure 1. The differentially expressed spots were cut manually and analyzed using MALDI‐TOF/MS. Mass spectrometry analysis showed the presence of PSA (shown by an arrowhead in Figure 1). However, for the other spots, only a spectrum of trypsin after autolysis was obtained. Therefore, to reduce the peak spectra derived from trypsin, experiments were reperformed by reducing the trypsin concentration ten times. However, we could not identify the peptides from the target spots. Therefore, we adopted the TMT proteomic analysis method. The results showed that several molecules were more than twice abundantly expressed in both AILNCaP14 and AILNCaP15 cells than in LNCaP cells (Table 1). Table 1 shows the top three molecules with higher expression levels in AILNCaP14 and AILNCaP15 than in LNCaP, excluding the myosin light chain kinase, which had an extremely low coverage (refer to supporting Table S1 for detailed results).

**TABLE 1:** The results of relative protein quantification (Top 3 molecules) with cell culture supernatant of LNCaP, AI‐14 and AI‐15 using tandem mass tag mass spectrometry

Accession	Description	Score	Coverage	^a^Peptides	Relative quantity			AAs	MW [kDa]	calc. pI
					AI‐14/LNCaP	AI‐15/LNCaP	AI‐15/AI‐14			
P03973	Antileukoproteinase OS=Homo sapiens GN=SLPI PE = 1 SV = 2 ‐ [SLPI_HUMAN]	219.07	43.18	8	4.744	6.723	1.533	132	14.3	8.75
Q99574	Neuroserpin OS=Homo sapiens GN=SERPINI1 PE = 1 SV = 1 ‐ [NEUS_HUMAN]	1394.86	61.71	22	3.301	5.223	1.534	410	46.4	4.91
O76038	Secretagogin OS=Homo sapiens GN=SCGN PE = 1 SV = 2 ‐ [SEGN_HUMAN]	249.01	42.03	10	2.15	2.868	1.303	276	32	5.41

Abbreviations: AAs, the number of amino acids; MW, Molecular Weight.

^a^ Peptides: the number of peptides, AI‐14: AILNCaP14, AI‐15: AILNCaP15,

### 3.2 Secretory leukocyte protease inhibitor (SLPI) expression is upregulated at both mRNA and protein levels in AILNCaP14 and AILNCaP15 cells

**FIGURE 2:**
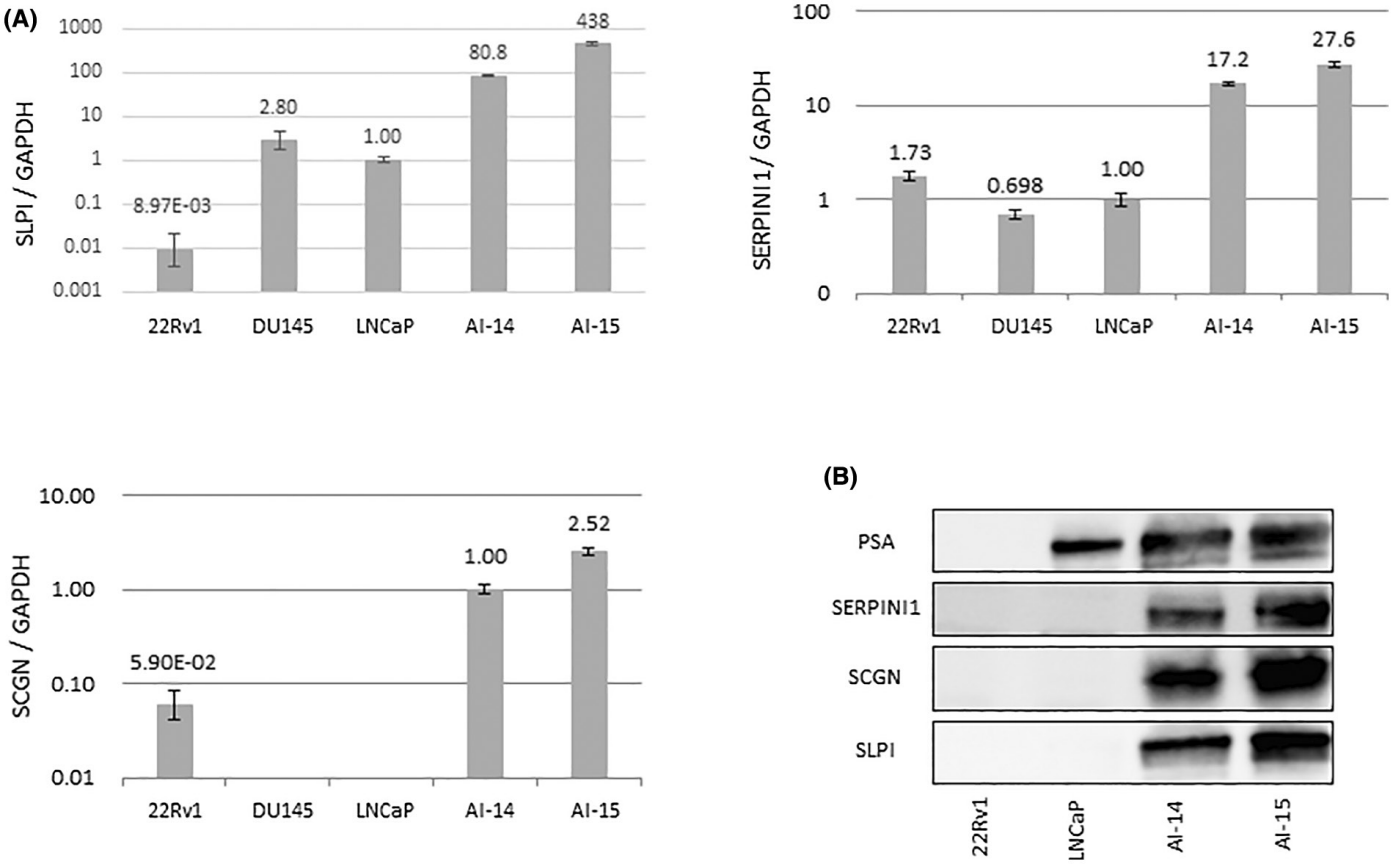
SLPI expression analysis in various prostate cancer cell lines. (A) Relative mRNA expression of SLPI, SERPINI1, and SCGN compared to GAPDH by RT‐PCR. In SLPI and SERPINI1, the relative quantity was based on LNCaP, but in SCGN, almost no expression was observed in LNCaP; therefore, AILNCaP14 (AI‐14) was used as a reference. (B) SLPI, SERPINI1, and SCGN protein expression in cell culture supernatants. SLPI: secretory leukocyte protease inhibitor, SERPINI1: neuroserpin, SCGN: secretagogin

Figure 2 shows the results of quantitative RT‐PCR (qRT‐PCR) and western blotting performed using various PC cell lines. Compared to those in LNCaP cells, the mRNA expression levels of three genes were upregulated in AILNCaP14 and AILNCaP15 (Figure 2A). Cell supernatant analysis showed high expression of these three proteins (Figure 2B). 2D‐western blotting was also performed using culture supernatants. The spots identified by chemiluminescence were at the approximately theoretical positions. The spots that were supposed to be SERPINI1 and SLPI could be detected in 2D gel stained by Coomassie; however, the spot of SCGN could not be identified in spite of using the more sensitive Flamingo staining (see supplementary Figure S1). Therefore, we focused on SLPI as a candidate marker to be used for prognosis and disease progression of PC.

### 3.3 Cell proliferation rate was significantly reduced in SLPI knockdown AILNCaP14 cell than that in its parental cells

Figure S2A shows the results of Western blotting of cells produced by transfecting AILNCaP14 with shSLPI or shScramble, and AILNCaP14 parent cells. There was no significant difference in AR expression, but PSA expression was slightly increased in shScrmble‐transfected cells. SLPI expression was significantly lower in shSLPI‐transfected cells than in shScramble‐transfected cells and their parent cells. Figure S2B shows the results of the cell proliferation assay. There was no difference in growth rate between parent cells and shScramble‐transfected cells. On the other hand, compared to parent cells and shSLPI‐transfected cells, the growth rate was significantly reduced in shSLPI‐transfected cells (*p* = 0.0168, *p* = 0.0197). These results showed that suppressing the expression of SLPI in AILNCaP14 has a negative effect on the cell proliferation.

### 3.4 Analysis of SLPI expression in human prostate cancer tissues

**FIGURE 3:**
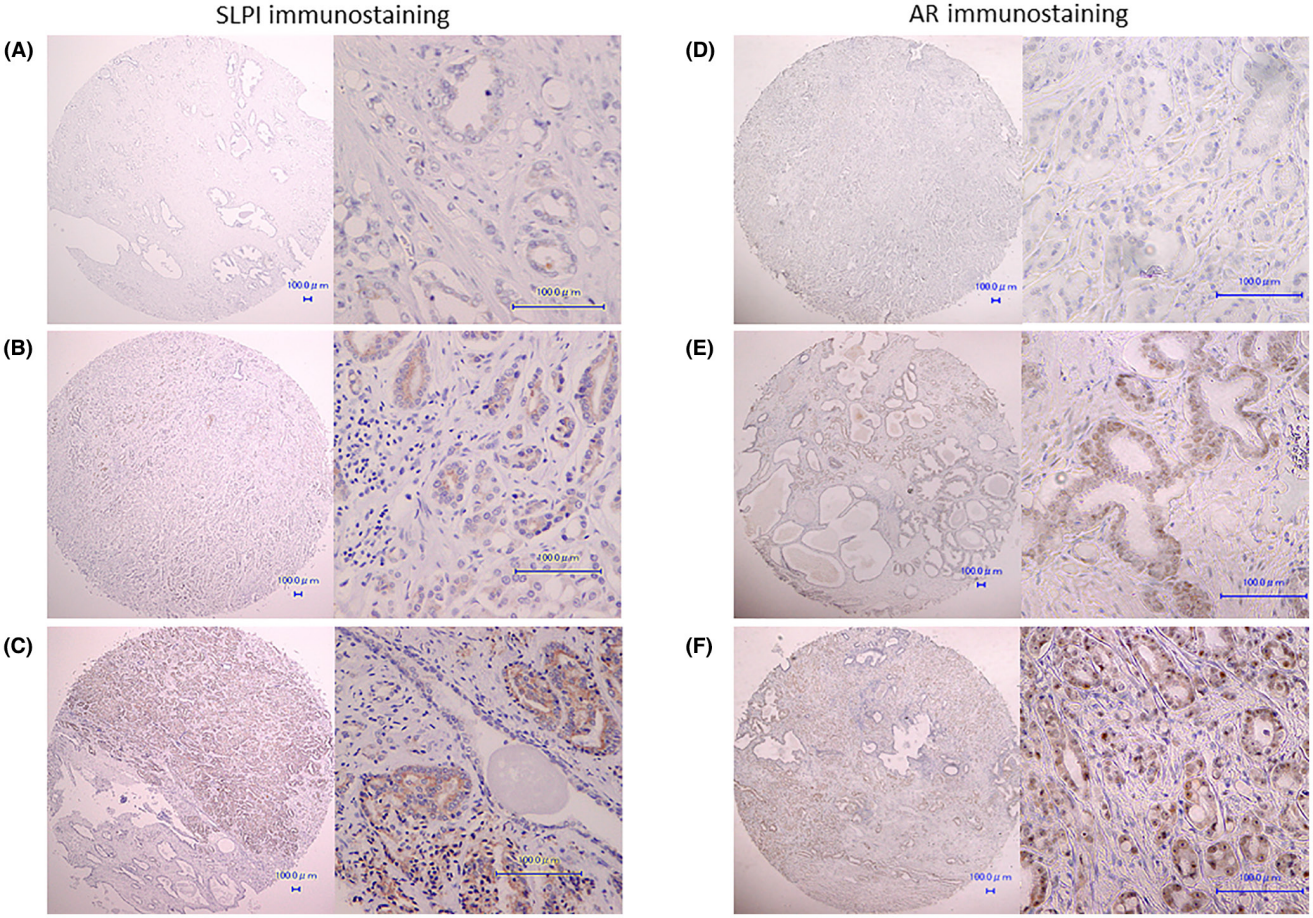
Representative SLPI and androgen receptor (AR) immunostaining. (A), (B), and (C) represent SLPI immunostaining, and (D), (E) and (F) represent AR immunostaining. The immunostaining scores of each sample are as follows; (A) total score (TS) 0 = intensity score (IS) 0 + proportion score (PS) 0, (B) TS 3 = IS 1 + PS2, (C) TS 6 = IS 2 + PS 4, (D) TS 0 = IS 0 + PS 0, (E) TS 3 = IS 1 + PS2, and (F) TS 5 = IS 1 + PS 4

**FIGURE 4:**
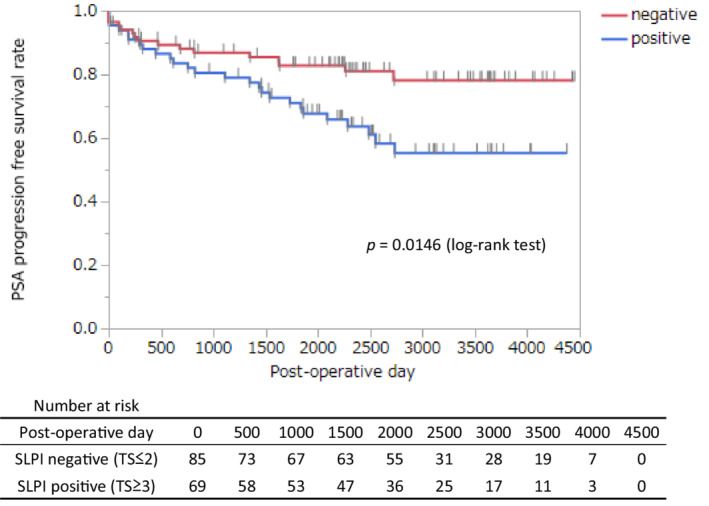
Kaplan–Meier survival curves of PSA progression‐free survival stratified by SLPI expression

The immunostaining of AR and SLPI was evaluated using a tissue microarray (TMA) prepared from 175 radical prostatectomy specimens, and correlation with various clinicopathological factors and postoperative prognosis was examined. Although the TMA consisted of 175 samples, only 154 contained cancer tissue. Representative immunostaining results for SLPI and AR are shown in Figure 3 and the results in Table 2. (Representative immunohistochemical expressions between AR and SLPI at the same spots is shown in supplementary Figure S3) Based on the results of IHC total score (TS), 154 cases were classified either as SLPI‐positive [TS ≥3, 69 cases (44.8%)] or SLPI‐negative [TS ≤2, 85 cases (55.2%)]. Table 3 shows the correlation of SLPI with age, PSA level before radical prostatectomy, pathological T stage, surgical margin, Grade Group, and AR total score. There was no significant correlation between SLPI expression and age, preoperative PSA level, T stage, surgical margin, and grade group; however, a positive correlation was observed between SLPI expression and AR total score when the cases were classified according to SLPI expression (TS≥3 and TS≤2, p = 0.0029). Survival curves plotted to examine the correlation between postoperative PSA recurrence and SLPI expression showed that PSA progression‐free survival was significantly lower in SLPI‐positive cases (TS≥3, *p* = 0.0146) (Figure 4). Prognostic factors for PSA progression‐free survival were evaluated using extracapsular extension, positive surgical margin, PSA level before radical prostatectomy, Grade Group, and SLPI expression. SLPI positivity was an independent risk factor for PSA recurrence (*p* = 0.0097, hazard ratio = 2.35) (Table 4). These results showed that SLPI expression in cancer cells may be a prognostic marker for patients undergoing radical prostatectomy.

**TABLE 2:** SLPI and AR total score distribution of TMA specimens

		Immunostaining	
		SLPI (*n* = 154)	AR (*n* = 154)
Total score	0	84	60
	2	1	26
	3	5	31
	4	9	17
	5	49	20
	6	6	0
	7	0	0

**TABLE 3:** Correlation between Clinicopathological features of TMA specimens and SLPI immunostaining results

		SLPI		*p* Value
		negative (*n* = 85)	positive (*n* = 69)	
Age (mean ± SD)		65.5 ± 6.14	66.4 ± 5.87	0.41^a^
PSA, ng/ml (median)		7.30 (IQR 5.40–10.6)	8.17 (IQR 6.42–11.0)	0.32^a^
Pathological T stage	T2a	4	5	0.93^b^
	T2b	1	1	
	T2c	43	37	
	T3a	30	21	
	T3b	7	5	
Surgical margin	Negative	52	36	0.26^b^
	Positive	33	33	
Grade group (pathological)	1	34	20	0.45^b^
	2	25	19	
	3	19	22	
	4	5	7	
	5	2	1	
AR total score	0	44	16	0.0029^b^
	2	9	17	
	3	17	14	
	4	7	10	
	5	8	12	
	6	0	0	
	7	0	0	

Abbreviations: IQR, interquartile range; SD, standard deviation.

^a^ Kruskal‐Wallis test,

^b^ Peason's chi‐squared test.

**TABLE 4:** Cox proportional hazard regression analysis of PSA progression free survival and clinicopathological variables

Variables	PSA progression free survival rate		*p* value^a^
	HR	95% CI	
Extracapsular extension	1.13	0.59–2.14	0.704
Positive surgical margin	1.82	0.95–3.53	0.0668
PSA level before RP	1.16	1.03–1.16	0.0014
Grade group	1.66	1.23–2.25	0.0005
SLPI positive (TS≥3)	2.33	1.23–4.55	0.0097

Abbreviations: CI, confidense interval; HR, hazard ratio.

^a^ Wald test.

### 3.5 Expression analysis of SLPI using human prostate cancer tissues derived from the same patients both under hormone naive and castration‐resistant conditions

**FIGURE 5:**
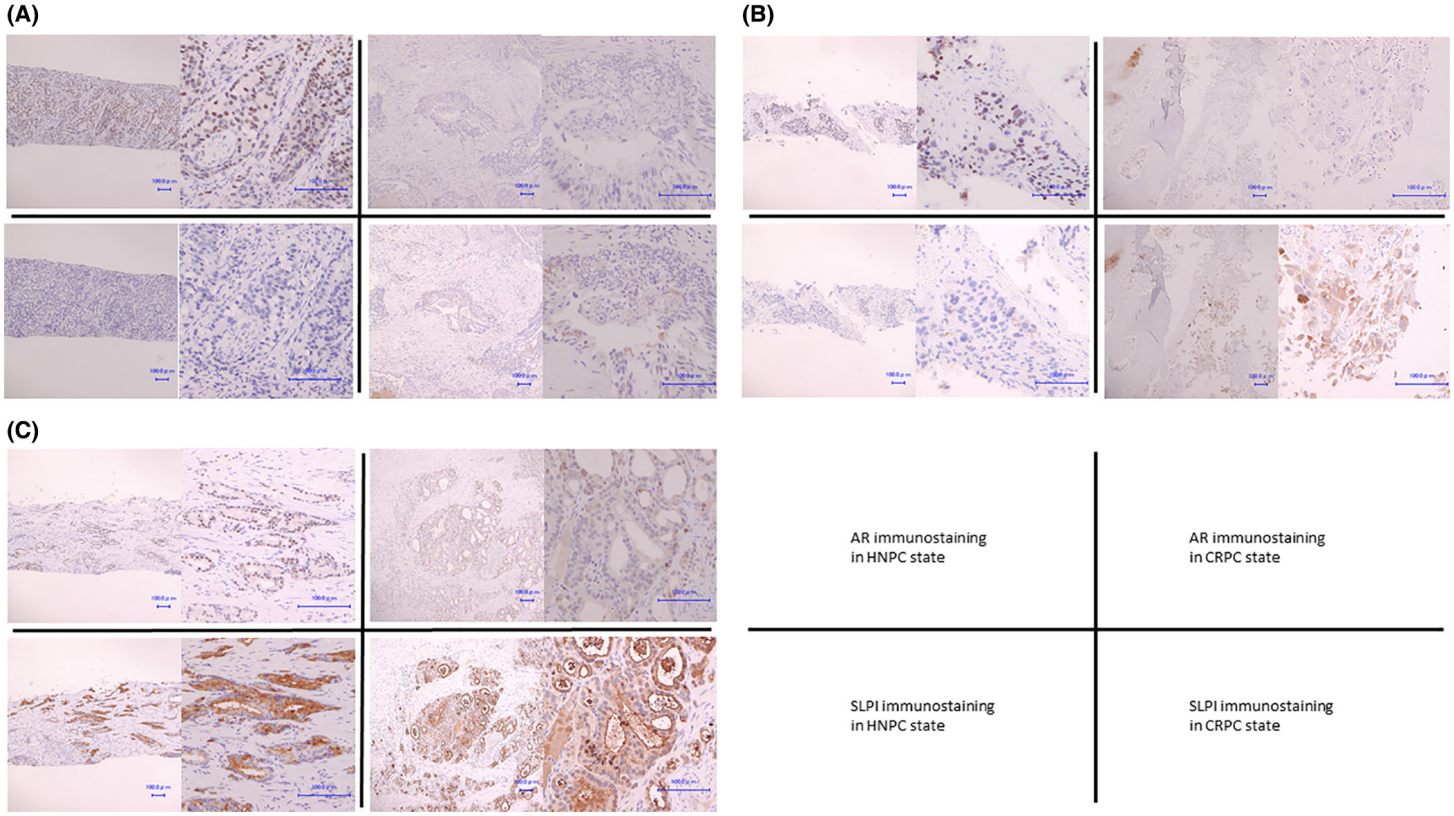
Immunostaining results of AR and SLPI at hormone‐naive prostate cancer (HNPC) and Castration resistant prostate cancer (CRPC) states in the same patients. AR expression was decreased in three patients with positive SLPI expression in the CRPC state. Figures A, B, and C represent cases 7, 8, and 11, respectively.

We obtained HNPC and CRPC tissues from the same patients in 11 cases^12^; clinical and pathological characteristics are presented in Table 5. Immunostaining scores for AR and SLPI in HNPC and CRPC conditions for each sample are shown in Table 6. In the HNPC state, AR was expressed in all cases, whereas SLPI was expressed in only one case. In the CRPC state, SLPI expression was observed in four cases, of which three cases had TS ≥3. The AR expression scores in the CRPC state of these three cases decreased in both IS and PS. In case 4, SLPI expression was TS = 2 and AR expression was TS = 7 in the CRPC state. However, AR expression was reduced at the site where SLPI was expressed, and vice versa (see supplementary Figure S4, case 4). In contrast, AR expression scores were the same or increased in the other cases. IHC images of cases with SLPI TS ≥3 are shown in Figure 5 (see supplementary Figure S4 for IHC images of all cases). The RNA‐seq dataset available through TCGA was used to calculate the correlation coefficients for SLPI and AR expression in CRPC patients. There was a weak negative correlation between SLPI and AR (*r* = −0.37, *p* < 0.0001) (supplementary Figure S5), which was consistent with our results. Thus, the results suggest that SLPI may increase in some cases during disease progression, and SLPI expression and AR expression may be negatively correlated.

**TABLE 5:** Clinicopathological features in 11 CRPC cases

Case	Age at Diagnosis	Clinical Stage at Diagnosis	Gleason score at diagnosis	Excised CRPC Organ	Age at Excision	PSA at Excision	Days from diagnosis to castration resistance	Treatment until Excision of CRPC Specimens	Whether Enz was used for treatment	Discrepancy between serum PSA levels and radiographic progression
Case 1	57	cT3bN1M0	3 + 3	Prostate	62	25	786	CAB + DOC	not used	not observed
Case 2	73	cT3bN0M0	3 + 4	Prostate	84	5	2860	CAB	not used	not observed
Case 3	60	cT3aN0M1c	4 + 4	Prostate	65	392	603	CAB + DOC	not used	not observed
Case 4	79	cT3aN0M0	4 + 4	Penis	82	16.1	1051	CAB	not used	not observed
Case 5	68	cT3aN0M1c	4 + 4	Thoracic vertebra	75	39.21	702	CAB + DOC + Abi	used	not observed
Case 6	70	cT3aN0M0	4 + 3	Prostate	78	408.2	2112	CAB	used	not observed
Case 7	76	cT4N1M1b	4 + 5	Prostate	85	14.07	2320	CAB + Enz	used	observed
Case 8	78	cT4N1M1b	5 + 4	Thoracic vertebra	79	7.58	321	CAB	not used	not observed
Case 9	69	cT4N0M0	4 + 5	Prostate	71	46.74	567	CAB	used	not observed
Case 10	80	cT3bN1M0	4 + 5	Prostate	82	6.21	544	CAB	used	not observed
Case 11	69	cT4N0M1b	4 + 5	Prostate	70	127	318	CAB + Enz	used	observed

Abbreviations: Abi, abiraterone; CAB, combined androgen blockade; DOC, docetaxel; Enz: enzalutamide.

**TABLE 6:** The results of AR and SLPI immunostaining score in 11 cases, including both hormone‐naïve and castration‐resistant specimens

Case	AR immunostaining score						SLPI immunostaining score					
	at HNPC			at CRPC			at HNPC			at CRPC		
	IS	PS	TS	IS	PS	TS	IS	PS	TS	IS	PS	TS
Case 1	1	2	3	3	4	7	0	0	0	0	0	0
Case 2	2	4	6	3	4	7	0	0	0	0	0	0
Case 3	2	4	6	3	4	7	0	0	0	0	0	0
Case 4	2	4	6	3	4	7	0	0	0	1	1	2
Case 5	2	4	6	2	4	6	0	0	0	0	0	0
Case 6	2	4	6	3	4	7	0	0	0	0	0	0
Case 7	2	4	6	1	1	2	0	0	0	2	1	3
Case 8	2	4	6	1	3	4	0	0	0	2	3	5
Case 9	2	4	6	2	4	6	0	0	0	0	0	0
Case 10	3	4	7	3	4	7	0	0	0	0	0	0
Case 11	2	4	6	1	3	4	3	3	6	3	4	7

### 3.6 Serum SLPI levels in benign prostatic hyperplasia and prostate cancer patients

**FIGURE 6:**
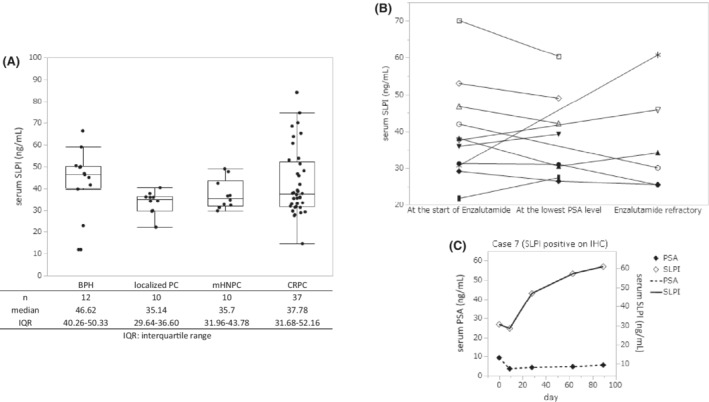
Serum SLPI levels. (A) Serum SLPI levels in benign prostatic hyperplasia (BPH) and prostate cancer patients. No significant difference was observed between the groups. (B) Consecutive serum SLPI measurements during enzalutamide treatment. No correlation was found between changes in serum PSA and serum SLPI levels in these 11 patients. (C) Consecutive serum SLPI and PSA measurements before enzalutamide treatment and after becoming refractory to it. Focusing on case 7 shown in Table 5, serum SLPI decreased after enzalutamide treatment started, but increased when his disease progressed; however, serum PSA did not reflect his disease progression after becoming enzalutamide‐resistant state.

The results of serum SLPI concentration measurement in 69 patients by ELISA are shown in Figure 6A. Although there was a tendency for serum SLPI levels to increase in CRPC patients compared to HNPC patients, no significant difference was found among all the groups. Consecutive blood sampling was performed in 11 of 37 CRPC patients. Serum SLPI was measured in each sample, and the change in SLPI value at three time‐points, i.e., (1) at the start of enzalutamide treatment, (2) when the PSA level was the lowest under enzalutamide administration (at the lowest PSA level), and (3) when the PSA level increased continuously with enzalutamide refractory state, are shown in Figure 6B. No correlation was found between disease states and serum SLIP levels in these 11 patients. In case 7 in Table 5, serum and tissue samples were also obtained both under HNPC and CRPC states. Interestingly, his serum SLPI levels increased significantly during enzalutamide treatment but in contrast, his PSA levels increased only slightly, and finally radiological disease progression were seen without PSA progression. Therefore, serum SLPI might be useful in limited cases as a disease progression marker of enzalutamide without PSA progression. (Figure 6C).

## 4 DISCUSSION

At present, there is very few or no serum marker that can predict the resistance to enzalutamide in CRPC patients. In order to discover those markers and reveal its mechanisms to the transition, CRPC cell lines derived from LNCaP by long term culturing under androgen‐deprived condition is good models. There are reports using these models showing that over‐expression of Enhancer of zeste homolog 2(EZH2) and decreased‐expression of histone demethylase lysin‐specific demethylase 5D (KDM5D) could be potential mechanisms of transition to CRPC.^14^, ^15^ AILNCaP14 and 15 cells are derivatives of LNCaP cells cultured for a long time in medium with charcoal‐stripped fetal bovine serum^16^ and can proliferate under enzalutamide treatment (Matsuoka T, et al. manuscript in preparation).

Therefore, we collected cell supernatants from those three cell lines to evaluate secreted proteins as serum marker candidates for patients with CPRC including the enzalutamide resistance cases. iTRAQ‐based quantitative proteomics revealed five molecules that were more than twice as abundant in the supernatants of AILNCaP14 and AILNCaP15 compared to those in LNCaP cells. Western blotting using a 2D‐PAGE gel was performed to investigate why the top three molecules could not be identified by 2D‐PAGE and MALDI‐TOF/MS analysis. As for SLPI, the spot itself was an unclear and large spot, which moved toward the edge when analyzed by IEF. Therefore, it may have contained impurities and could not be separated properly. SERPINI1 was recognized as a spot heavier than its theoretical molecular weight. It might have existed in a post‐translational modified form, and the position detected by western blotting might also have contained other different proteins, which made it difficult to be detected by MS analysis. Furthermore, spots for SCGN could not be identified even with Flamingo staining, which is more sensitive than CBB staining; therefore, it was speculated that the amount expressed was very low.

SLPI was originally discovered in human parotid secretions as a potent leucocyte protease inhibitor.^17^ *SLPI* belongs to a gene family coding for proteins with a so‐called whey acidic protein (WAF) motif. It is physiologically produced and secreted by the mucosa of respiratory, gastrointestinal, and reproductive organs, and functions as an anti‐inflammatory molecule with a role in defense against bacterial infection and inflammation, cell repair, and epithelial proliferation.^18^ Some indicators of systemic inflammation and nutrition status have also been reported to be useful in predicting the therapeutic prognosis of malignant tumors, such as the neutrophil/lymphocyte ratio, the C‐reactive protein/albumin ratio, and the platelet/hemoglobin ratio, that can be easily measured with conventional devices.^19^, ^20^ Modulated expression of SLPI has been reported in various human malignancies, such as gastric cancer, thyroid cancer, lung cancer, ovarian cancer, endometrial cancer, and oral cancer.^21^, ^22^, ^23^, ^24^, ^25^, ^26^, ^27^ Its expression has been reported as context‐dependent; however, SLPI overexpression is commonly associated with high‐risk aggressive cancer from various organs.^22^, ^28^, ^29^ The mechanisms of SLPI in cancer cell proliferation and metastasis have been previously reported. SLPI interacts with the retinoblastoma tumor suppressor protein and releases FoxM1 from the Rb‐FoxM1 complex, which may activate FoxM1 target genes involved in breast cancer metastasis^28^ SLPI can affect not only tumor cell characteristics, but also blood vessel formation.^30^ It increases *cyclin D1* gene expression and negatively influences anti‐proliferative and proapoptotic insulin‐like growth factor‐binding protein‐3 expression.^26^

Focusing on C4‐2B, an androgen receptor‐dependent but androgen‐independent cell line, Decker et al. demonstrated that SLPI expression was regulated by androgen receptors in a ligand‐independent manner, and upon SLPI‐specific siRNA transfection, it resulted in growth attenuation by increased caspase 3 and 7 activity in C4‐2B cells.^31^ They also reported that SLPI expression promotes CRPC cell survival and growth after androgen deprivation in vitro and *in vivo*.^32^ We also created stable SLPI knockdown AILNCaP14 cells by transfecting with shRNA. The growth rate of SLPI knockdown cells were significantly reduced as compared with the parental cells and the control cells transfected by scrambled shRNA (supplementary Figure S2). Summarized these manuscripts and our results, SLPI is positively regulated by AR with ligand independent manner in CRPC cells and its expression support CRPC cells proliferation under androgen deprived condition. In contrast, Yang et al. revealed that PC3 cells overexpressing the androgen receptor (PC3AR) showed a decrease in SLPI compared to parental cells. Moreover, 10 nM dihydrotestosterone treatment reduced SLPI expression, but addition of enzalutamide induced SLPI expression in PC3AR cells. They concluded that the androgen receptor negatively regulates SLPI through miR‐525‐5p and inhibits vasculogenic mimicry formation using PC3AR cells.^33^ Cell proliferation of PC3AR cells is independent of the AR itself; thus, the relationship between AR and SLPI might be different, depending on how AR participates in proliferation of the cells analyzed. This hypothesis may be partly supported by SLPI and androgen receptor expression in human PC tissues. Our prostatectomy tissue analysis showed a positive relationship with AR and SLPI expression, which are basically HNPC and AR‐dependent cancers. In contrast, analyses of 11 CRPC tissue expression revealed that most of the SLPI‐positive CRPC specimens showed reduced AR expression compared to the paired HNPC specimens. These CRPC specimens may have been transformed to androgen receptor‐independent proliferative state. Among these 11 matched samples in HNPC and CRPC states, four CRPC cases were positive for SLPI expression. Interestingly, tissues from two CRPC cases after becoming refractory to enzalutamide treatment showed increased SLPI expression compared to those from the tissues before enzalutamide treatment corresponding ones. Additionally, in one case, serum SLPI increased after becoming refractory to enzalutamide. Here we further reviewed the medical history of four SLPI‐positive cases. Two cases died of PC without use of enzalutamide as a treatment for CRPC, and PSA was also elevated in correlation with the disease progression (case 4 and case 8). On the other hand, enzalutamide was used as a treatment for CRPC in the remaining two cases (case 7 and case 11). Before enzalutamide administration, PSA also increased in correlation with the progression of the disease, but after using enzalutamide, a discrepancy appeared between the radiographic progression of the disease and the increase in their serum PSA levels. Moreover, among all 11 cases, only these 2 cases had such a discrepancy between serum PSA levels and radiographic progression (refer to Table 5). According to clinical trial of enzalutamide for CRPC patients, significant number of patients had radiological disease progression without PSA progression.^34^, ^35^ Considering our experimental results, increase in SLPI expression might correlate with a discrepancy between serum PSA levels and radiographic progression.

Sixty‐nine of the 154 (44.8%) localized PCa patients were SLPI positive, whereas only 1 of 11 (9.1%) CRPC patients were positive during the HNPC state. The immunostainability of SLPI is heterogeneous in prostate cancer tissue. (see supplementary Figure S3 and S4). This heterogeneity can be detected when we used whole mount specimens for IHC. However, using prostate biopsy specimens as representatives of HNPC cells, we may miss SLPI positive cells. We think that this is a reason why there was a discrepancy in SLPI positivity between HNPC prostatectomy specimens and mHNPC biopsy ones. However, due to the small number of cases, further research is needed.

When we consider relation with SLPI expression and neuroendocrine differentiation, we analyzed expressions between AR, SLPI, and ENO2 (neuron specific enolase, a marker of neuroendocrine differentiation) from the available datasets of TCGA using RNA‐seq data derived from CRPC patients with and without neuroendocrine differentiation,^36^ but we could not find a definitive tendency (data not shown).

There have been no previous reports on SLPI expression in localized PC. In this study of postoperative PSA progression‐free survival rate in patients who underwent radical prostatectomy, it was found that the expression of SLPI is an independent risk factor for PSA recurrence. In localized PC patients, who were not androgen‐ deprived, SLPI expression and AR expression were positively correlated and may have affected tumor growth; nevertheless, further research is needed.

Zheng et al. showed that serum SLPI levels were significantly elevated in metastatic CRPC patients compared to HN patients.^32^ They also reported that SLPI levels in eight matched serum samples before and after androgen deprivation therapy increased significantly. This is in contrast with our results that our CRPC serum samples did not show an increase in SLPI protein levels, in spite of using the same ELISA kit. Zheng et al. reported that the serum levels of SLPI in mCRPC patients were approximately 70 ng/ml, but our samples showed around 37.78 (IQR 31.68–52.16) ng/ml. However, the number of sera analyzed was too small to draw definite conclusions and further experiments are required.

In conclusion, we found that SLPI is abundant in supernatants of LNCaP cells derivatives, which are resistant to enzalutamide treatment. Some CRPC tissues showed increased SLPI expression compared to the matched HNPC tissues. Therefore, SLPI may be employed as a marker for CRPC, especially in patients who are resistant to enzalutamide treatment.

## AUTHOR CONTRIBUTIONS

Conceived and designed the study: YM and TI. Analyzed and Interpretation of the data: YM and TI. Drafted the manuscript: YM and TI. Acquisition of the data: YM, TG, XL, KN, KO, MT, KM, HK, MU, TS and YT. Revising the manuscript with critically important intellectual contents: SA, TK, and OO. All the authors read and approved the final version of the manuscript.

## CONFLICT OF INTEREST

The authors have no conflict of interest.

## ETHICAL APPROVAL STATEMENT

All experiments in humans were approved by the Ethics Committee of Kyoto University Hospital (G1083) and were performed in accordance with the Japanese Ethical Guidelines for Human Genome/Gene Analysis Research and Ethical Guidelines for Medical and Health Research involving Human Subjects. The study included no clinical trial but only retrospective observation one (approvals included in G1083). Written informed consent was obtained from all patients.

## Supporting information


Appendix S1

Figure S1
Figure S2
Figure S3
Figure S4
Figure S5

Table S1

## Data Availability

The data that support the findings of this study are available in the supplementary material of this article.
