# Supplementary material for: Up‐regulation of secretory leukocyte protease inhibitor in human samples might have a potential role of predicting prostate cancer recurrence and progression after surgery and hormonal therapy
Source: Cancer Med. 2022 Aug 13;12(3):3328–42. doi: 10.1002/cam4.5134 (PMC9939162; doi:10.1002/cam4.5134)
Supplement: Supplementary file 1 — Appendix S1 [file CAM4-12-3328-s003.docx]

**Doc. S1**

**Supporting Materials and Methods**

**Sample preparation for proteomic analyses**

To denature the samples, 5.4 mL of denaturation agent (9 M urea, 2% CHAPS, 50 mM Tris pH 9) was added to 13.5 mL of the collected culture supernatants, and the mixtures were rotated at 4 °C for 30 min. Next, the samples were applied to Amicon Ultra-15 3000 NMWL (Merck KGaA, Darmstadt, Germany) filter units and concentrated at 4 °C, 4000 × *g* for 60 min using a swinging rotor. When the samples were concentrated to about 0.5 mL, 5 mL of 100 mM ammonium bicarbonate was added and the samples were concentrated again at 4 °C, 4000 × *g* for 60 min. The culture supernatants were desalted and concentrated by repeating the above-mentioned procedure three times. The concentrates remaining on the columns were collected in PROTEOSAVE^TM^ microtubes (Sumitomo Bakelite Co., Ltd., Tokyo, Japan). Meanwhile, the filter membranes were washed with 100 µL of 1 M ammonium bicarbonate several times, and the washed solutions were added to the concentrates in the microtubes. The samples were dried using a centrifugal concentrator (EYELA CVE-3000, Tokyo Rikakikai, Tokyo, Japan) connected with a diaphragm vacuum pump (MD1C model, Vacuubrand, Germany) and a cold trap (EYELA Uni Trap UT-2000, Tokyo Rikakikai, Tokyo, Japan) for about 3 h.

**Matrix assisted laser desorption/ionization-time of flight mass spectrometry (MALDI-TOF/MS)**

The spots that exhibited differences between LNCaP and AILNCaP14 or AILNCaP15 cells in image analysis were excised, destained with acetonitrile, reduced with DTT, carbamidemethylated with iodoacetamide, and digested with trypsin for mass spectrometry (APRO SCIENCE, Tokushima, Japan). To stop the trypsin reaction in the gel, add 0.5 µL of 100% trifluoroacetic acid (TFA) to the digested sample, stir and collect the supernatant. Next, to re-suspend digested molecules, the gel pieces after the above reaction were added to 0.1% TFA 50 µL and placed in an ultrasonic cleaner for 30 minutes to react, and the supernatant was collected. The collected solutions were dried using a centrifugal concentrator. After sample purification using ZipTip C18 (Millipore, Bedford, MA, USA), the sample solutions were mixed with 2, 5-dihydroxybenzoic acid (DHB) as a matrix and analyzed using two MALDI-TOF mass spectrometers (AXIMA Resonance [MALDI-qTOF/MS] and/or Performance [MALDI-TOF/TOF/MS], Shimadzu Corporation, Kyoto, Japan). The data were analyzed using the Mascot search engine (Matrix Science, London, UK).

**Quantitative RT-PCR (qRT-PCR)**

Total RNA was isolated using a RNeasy Mini Kit (QIAGEN N.V., Venlo, Netherlands), and cDNA was synthesized from 1 µg of RNA using a First-Strand cDNA Synthesis kit (FSQ-101; TOYOBO. CO., LTD. Osaka, Japan). PCR was performed using SYBR green PCR Master Mix (Applied Biosystems, Waltham, MA, USA) and monitored in triplicate using the Thermal Cycler Dice Real Time System II (TP900; Takara Bio Inc., Shiga, Japan). The thermal cycling conditions were as follows: initial heating to 95°C for 10 min followed by 40 cycles of denaturation at 95°C for 15 s, annealing at 60 °C for 30 s, and extension at 72 °C for 30 s. The values were normalized to the levels of glyceraldehyde-3-phospate dehydrogenase (GAPDH). The following primer sequences were used: SLPI, 5'-AGTCACTCCTGCCTTCACCAT-3' (sense) and 5'-GCACTGGGCAGATTTCTTAGG-3' (antisense); neuroserpin (SERPINI1), 5'-CTGAGGAAGCCATTGCTGACT-3' (sense) and 5'-AGTTCCATCATTCCCATTGCA-3' (antisense); secretagogin (SCGN), 5'-AGAAGTGGATGGGTTTGTC-3' (sense) and 5'-CTGGGATTATGGGTTGATT-3' (antisense); and GAPDH, 5'-GAATATAATCCCAAGCGGTTTG-3'(sense) and 5'-ACTTCACATCACAGCTCCCC-3' (antisense).

**Antibodies**

Anti-PSA (C-19: sc7638) antibodies for Western blotting were obtained from Santa Cruz Biotechnology (Dallas, TX, USA). Anti-β-actin antibody (AC-15: ab6276) was purchased from Abcam PLC (Cambridge, UK). Anti-AR (N-20: sc-816), Anti-AR (C-19: sc-815) and Anti-SLPI (N-14: sc-10534) for Western blotting was purchased from Santa Cruz Biotechnology. Anti-SERPINI1 antibody (12558-1-AP) and anti-SCGN antibody (14919-1-AP) for Western blotting were purchased from Proteintech (Rosemont, IL, USA). Anti-AR antibody (CST #5153) for immunohistochemistry was obtained from Cell Signaling Technology, Inc. (Danvers, MA, USA). And anti-SLPI antibody (ab17157) for immunohistochemistry was purchased from Abcam PLC.

**Western blotting**

The dried culture supernatants (prepared in 2.2.1) were dissolved in RIPA buffer, supplemented with a protease inhibitor cocktail (Nacalai Tesque, Kyoto, Japan), and the protein concentration was estimated using a protein assay reagent kit (Bio-Rad Laboratories, Inc.). Concentrated culture supernatants were subjected to SDS-PAGE and transferred to polyvinylidene difluoride membranes (Bio-Rad Laboratories, Inc.). Membranes were immunoblotted with primary antibodies, followed by horseradish peroxidase-conjugated secondary antibodies, and developed for reading by enhanced chemiluminescence (Thermo Fisher Scientific Inc.). The dilutions of the primary antibodies used were as follows: PSA, 1:400; SLPI, 1:400; SERPINI1, 1:500; SCGN, and 1:500. Densitometric analysis was performed using an image analyzer (Fujifilm Holdings Corporation, Tokyo, Japan).

**Immunohistochemical analysis**

The proportion score (PS) was evaluated according to the amount of expression of stained tumor cells as follows: < 1% (score 0), 1–10% (score 1), 11–33% (score 2), 34–66% (score 3), and > 67% (score 4). The intensity score (IS) was evaluated as none (score 0), weak (score 1), intermediate (score 2), and strong (score 3) in most immunostained cells. The Gleason score (GS) of hematoxylin and eosin staining was also evaluated by a urological pathologist. Using this scoring system, we evaluated the correlation of SLPI immunostaining of cancer cells with PSA progression-free survival (PFS) after radical prostatectomy and compared SLPI and AR immunostaining in consecutive specimens of cancer cells in each individual.
