# Supplementary material for: Up‐regulation of secretory leukocyte protease inhibitor in human samples might have a potential role of predicting prostate cancer recurrence and progression after surgery and hormonal therapy
Source: Cancer Med. 2022 Aug 13;12(3):3328–42. doi: 10.1002/cam4.5134 (PMC9939162; doi:10.1002/cam4.5134)
Supplement: Supplementary file 2 — Figure S1 Figure S2 Figure S3 Figure S4 Figure S5 [file CAM4-12-3328-s002.pdf]

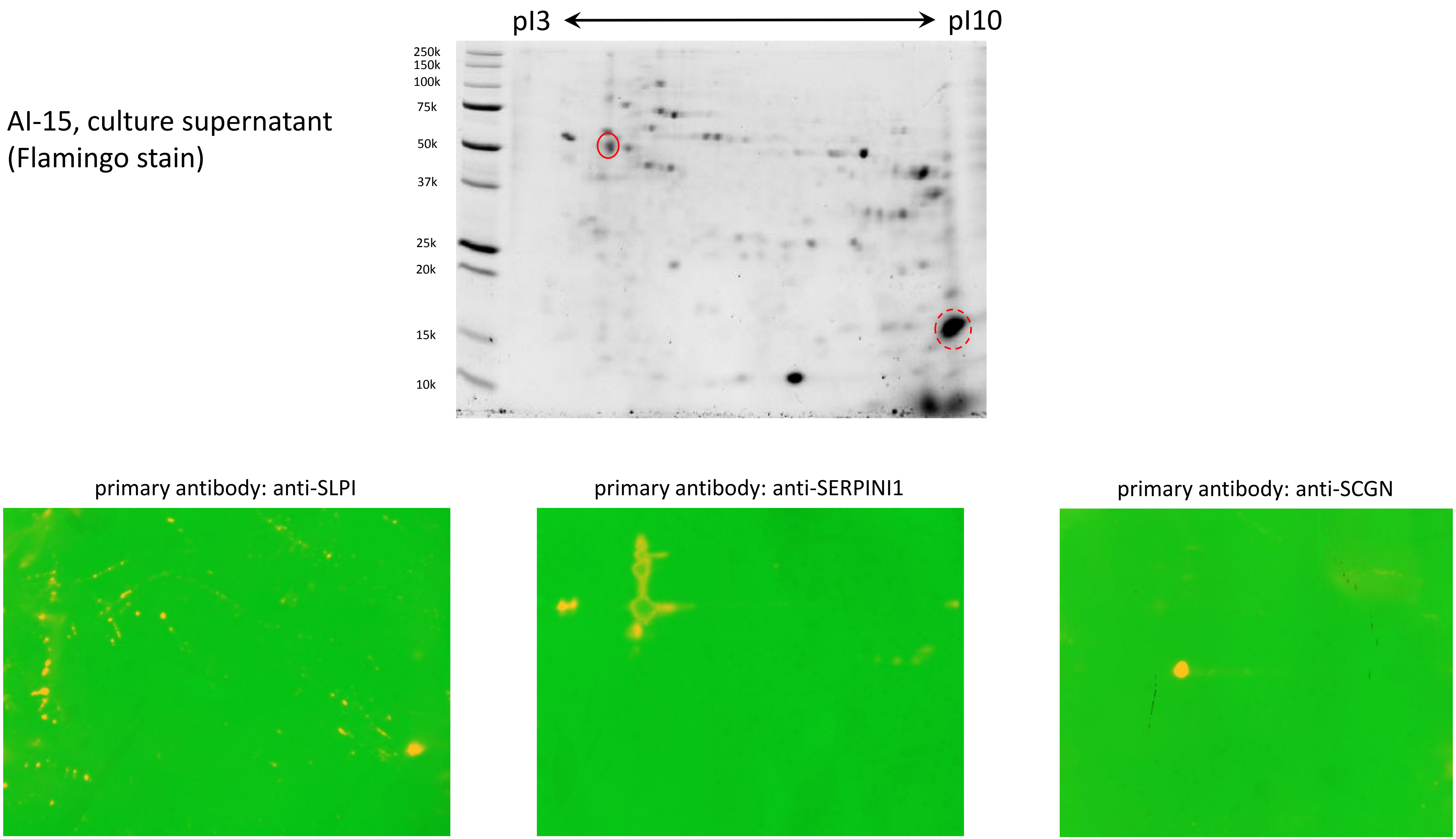

SLPI: MW 14kDa, theoretical pI 8.75

SERPINI1: MW 46kDa, theoretical pI 4.91

SCGN: MW 32kDa, theoretical pI 5.41

Figure S1

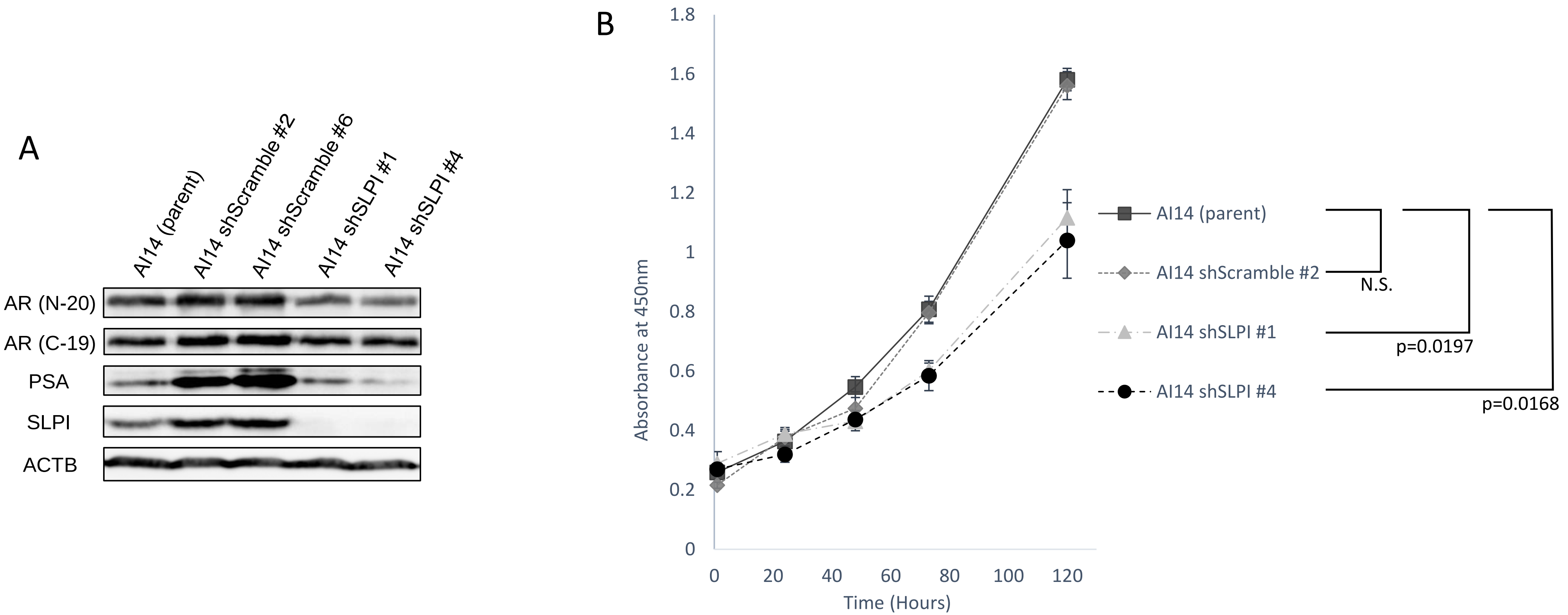
A
AI14 (parent)
AI14 shScramble #2
AI14 shScramble #6
AI14 shSLPI #1
AI14 shSLPI #4
AR (N-20)
AR (C-19)
PSA
SLPI
ACTB
B
Absorbance at 450nm
Time (Hours)
AI14 (parent)
AI14 shScramble #2
AI14 shSLPI #1
AI14 shSLPI #4
N.S.
p=0.0197
p=0.0168

Figure S2

TMA patient No. 2

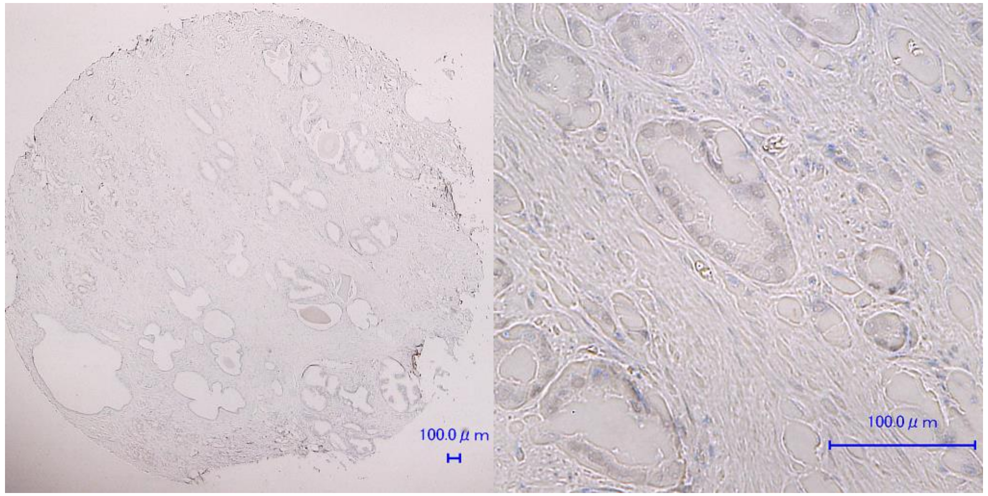

AR immunostaining, TS 0 = PS 0 + IS 0

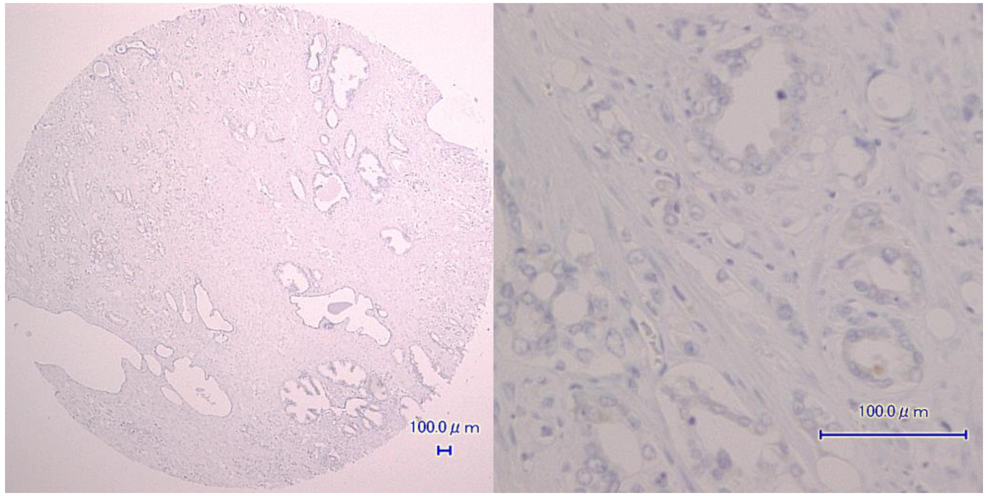

SLPI immunostaining, TS 0 = PS 0 + IS 0

TMA patient No. 27

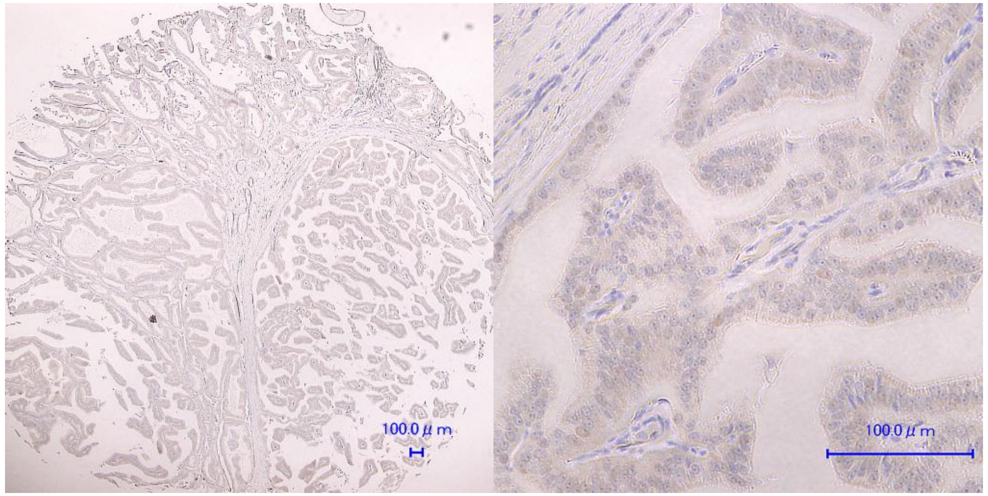

AR immunostaining, TS 0 = PS 0 + IS 0

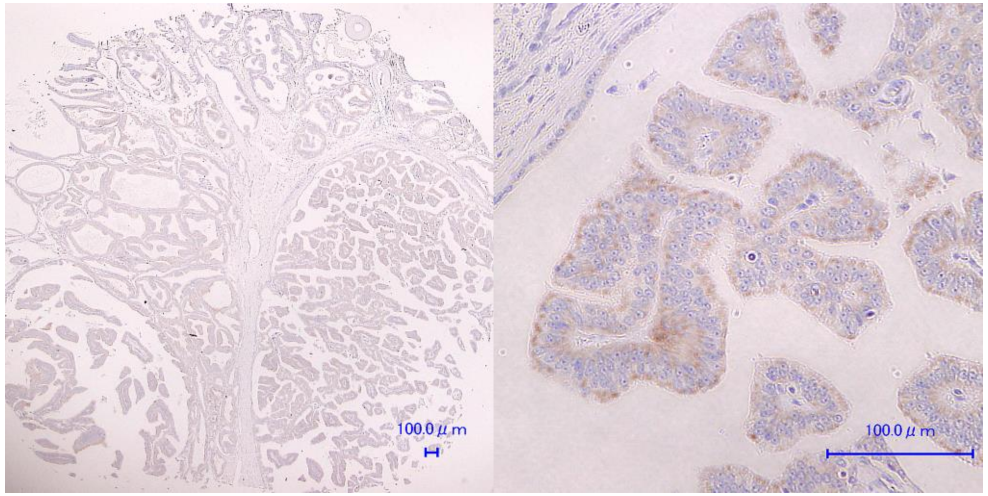

SLPI immunostaining, TS 0 = PS 4 + IS 1

Figure S3_1

TMA patient No. 35

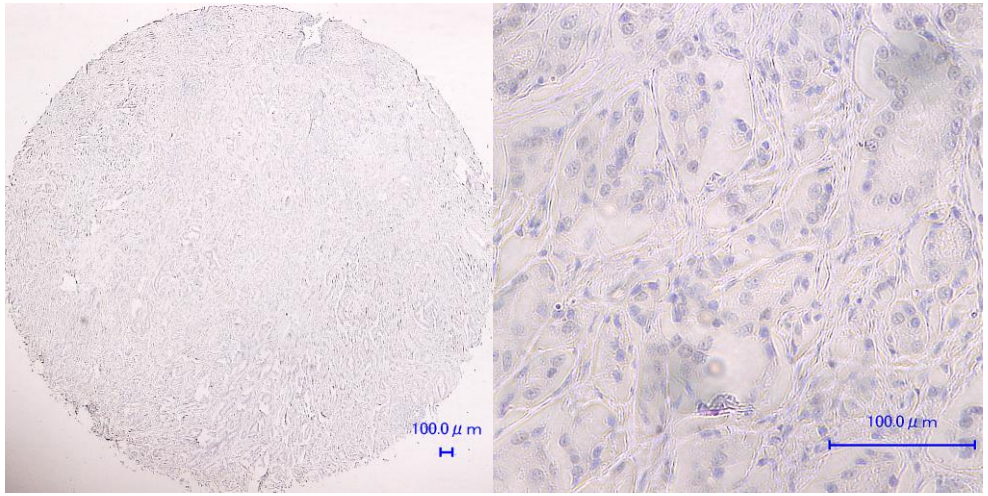

AR immunostaining, TS 0 = PS 0 + IS 0

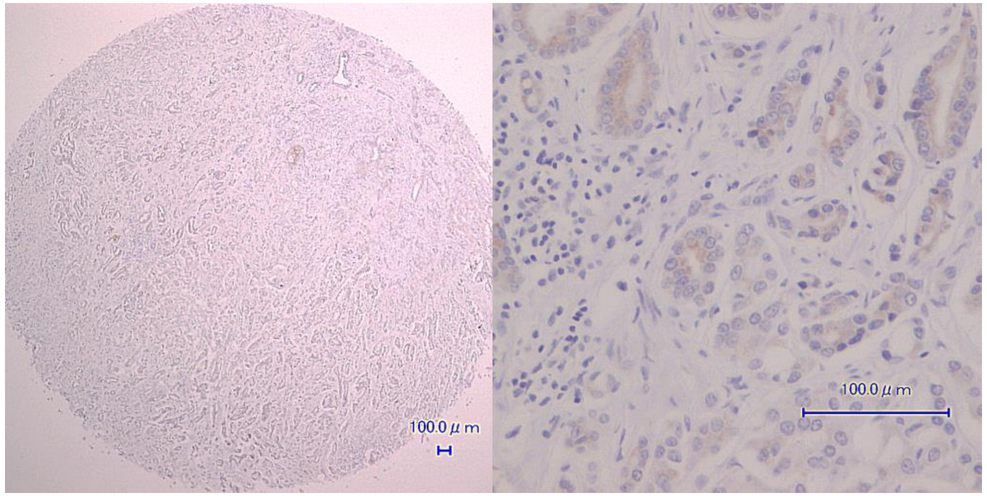

SLPI immunostaining, TS 3 = PS 2 + IS 1

TMA patient No. 62

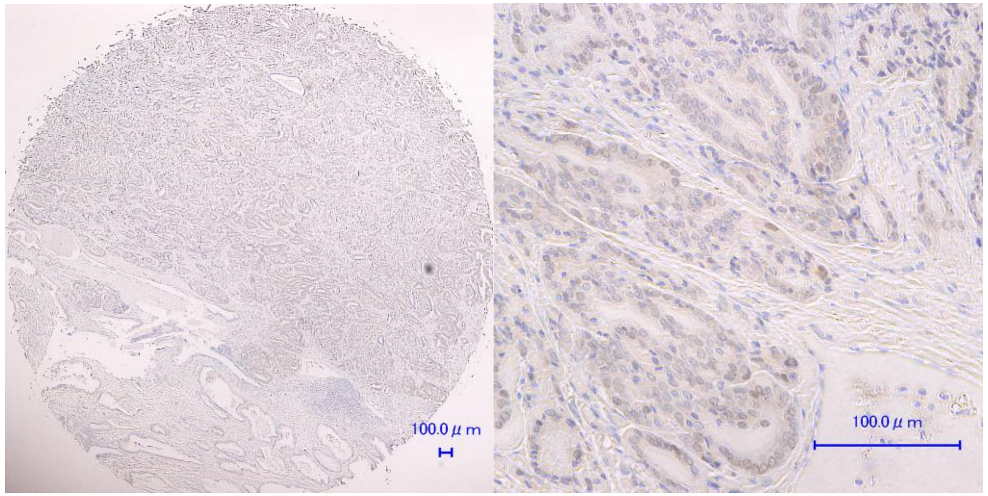

AR immunostaining, TS 2 = PS 1 + IS 1

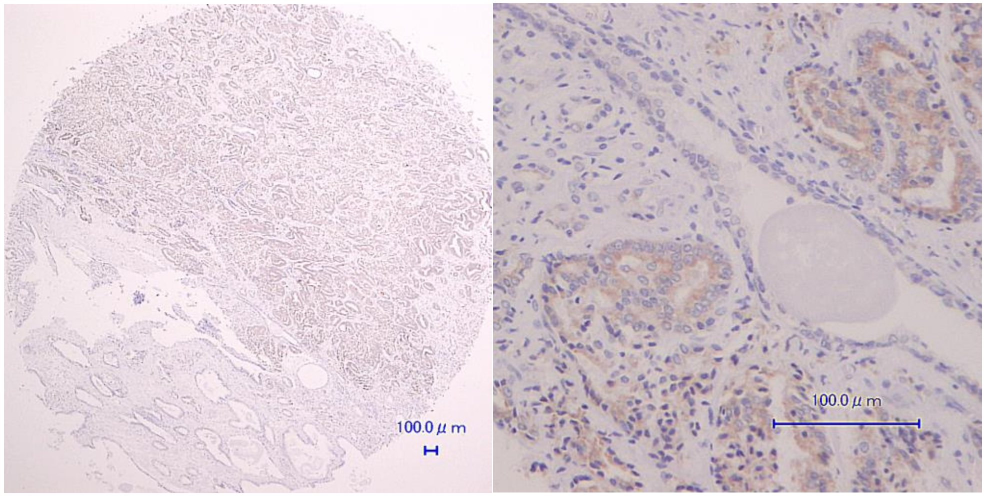

SLPI immunostaining, TS 6 = PS 4 + IS 2

Figure S3_2

TMA patient No. 20

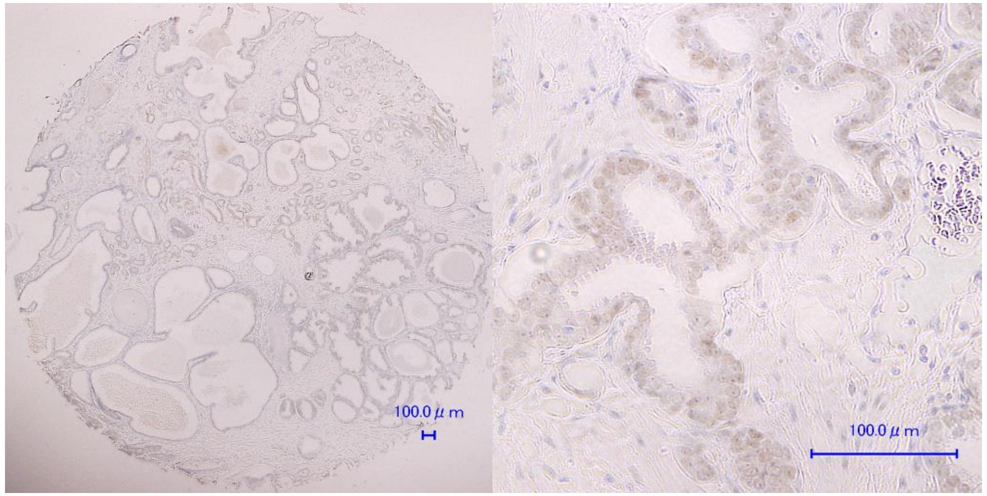

AR immunostaining, TS 3 = PS 2 + IS 1

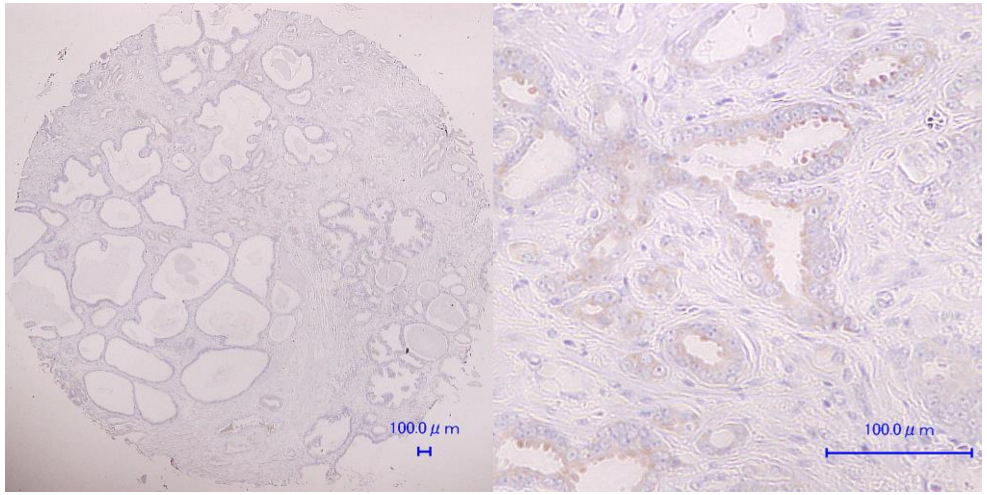

SLPI immunostaining, TS 4 = PS 3 + IS 1

TMA patient No. 40

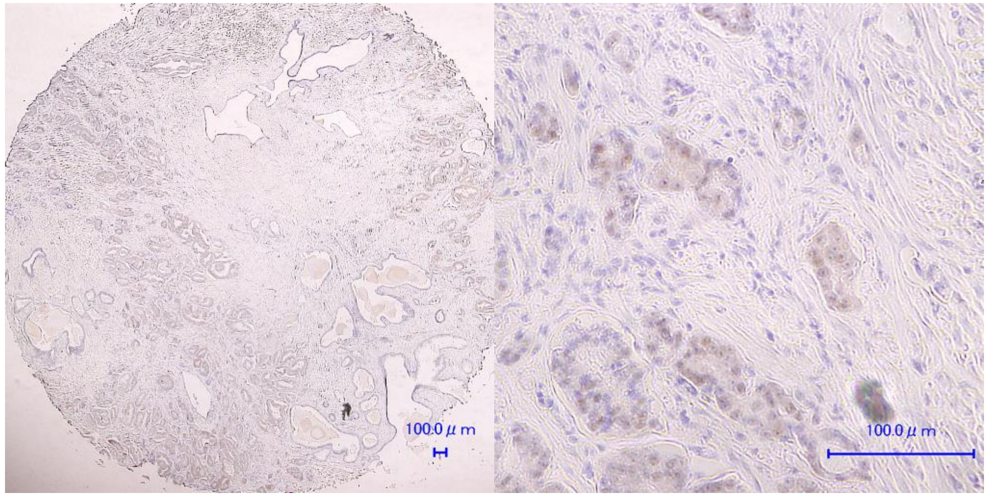

AR immunostaining, TS 4 = PS 3 + IS 1

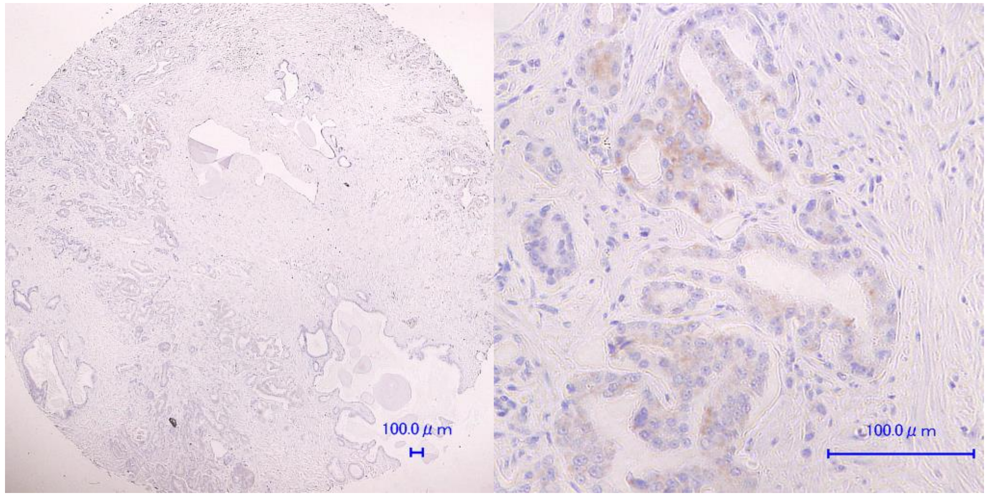

SLPI immunostaining, TS 2 = PS 1 + IS 1

Figure S3_3

TMA patient No. 82

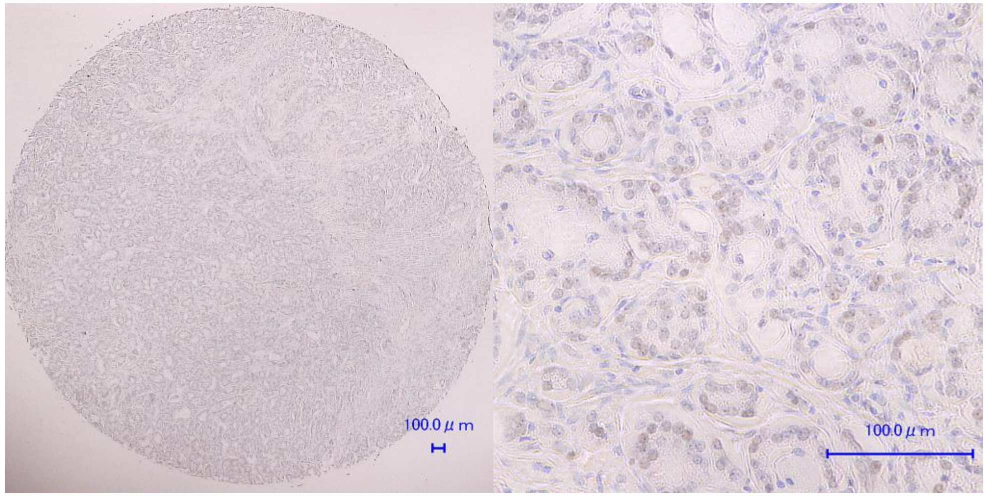

AR immunostaining, TS 4 = PS 3 + IS 1

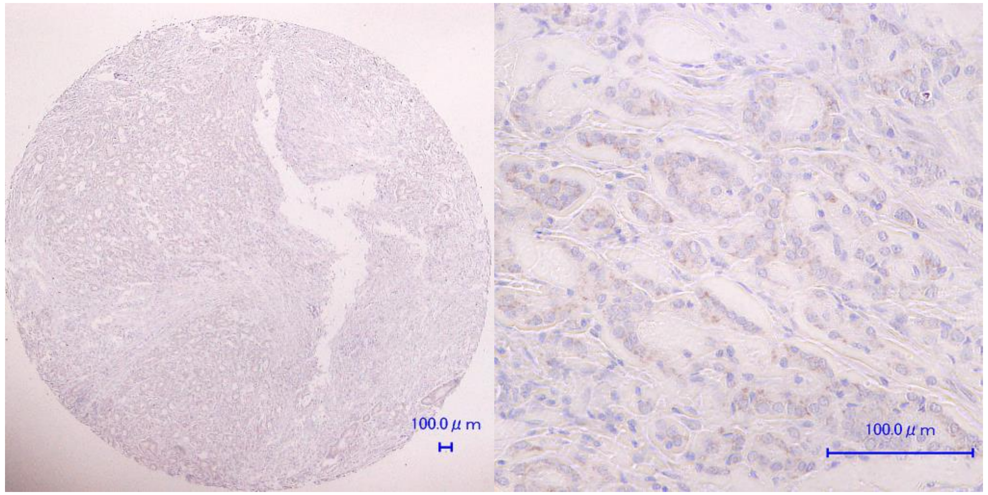

SLPI immunostaining, TS 5 = PS 4 + IS 1

TMA patient No. 6

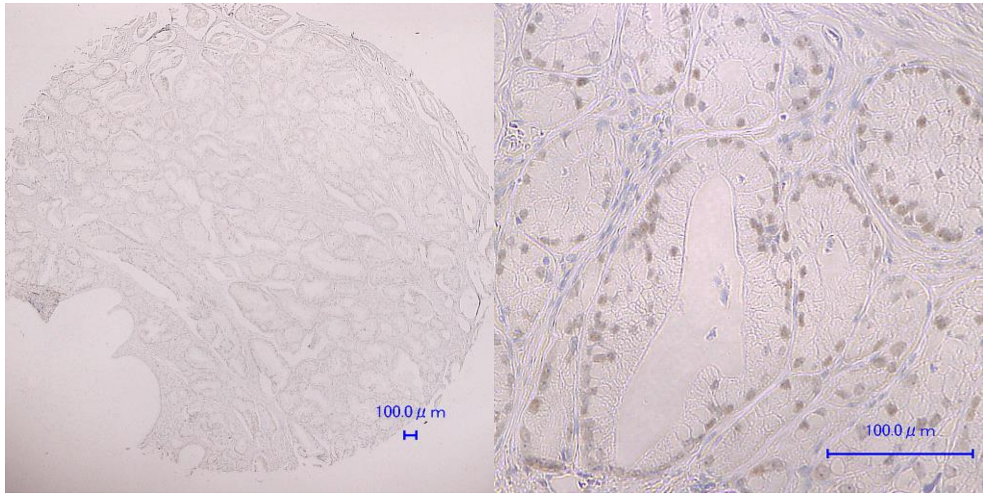

AR immunostaining, TS 5 = PS 4 + IS 1

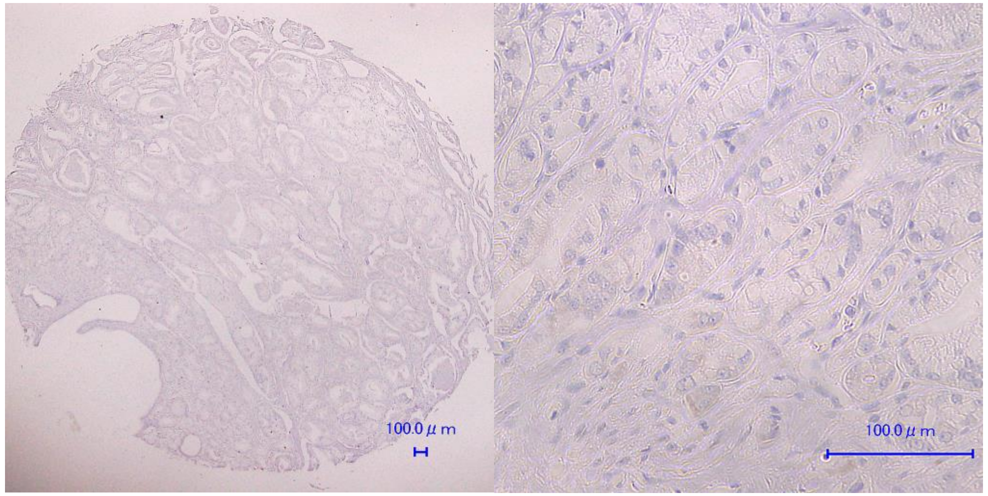

SLPI immunostaining, TS 0 = PS 0 + IS 0

Figure S3_4

TMA patient No. 107

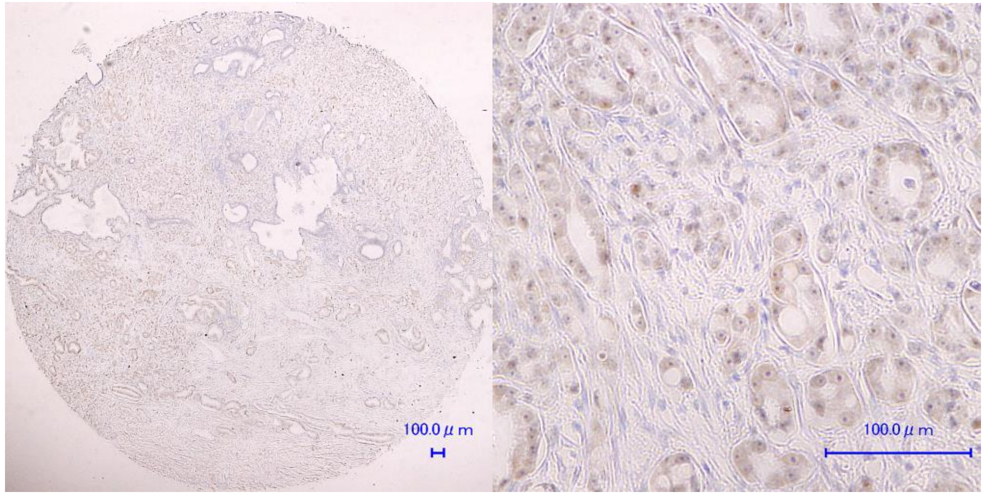

AR immunostaining, TS 5 = PS 4 + IS 1

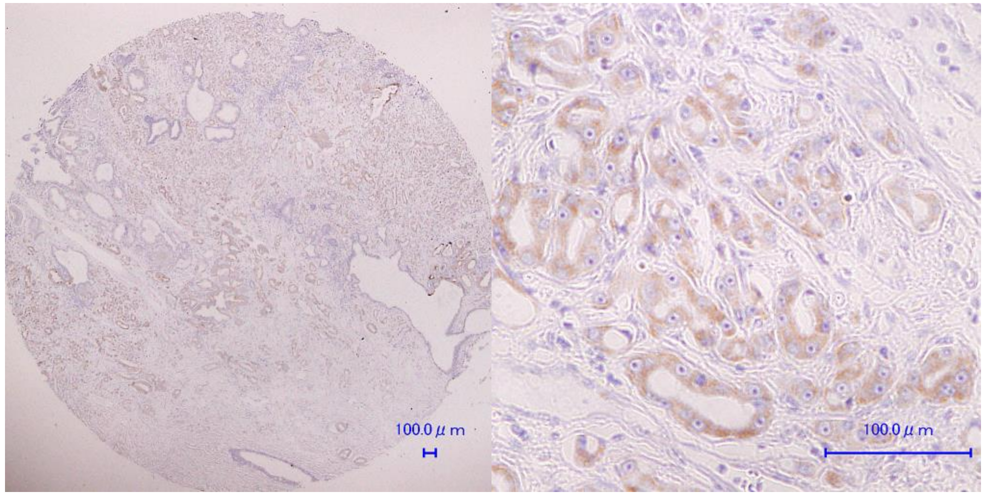

SLPI immunostaining, TS 6 = PS 4 + IS 2

Figure S3_5

Case 1

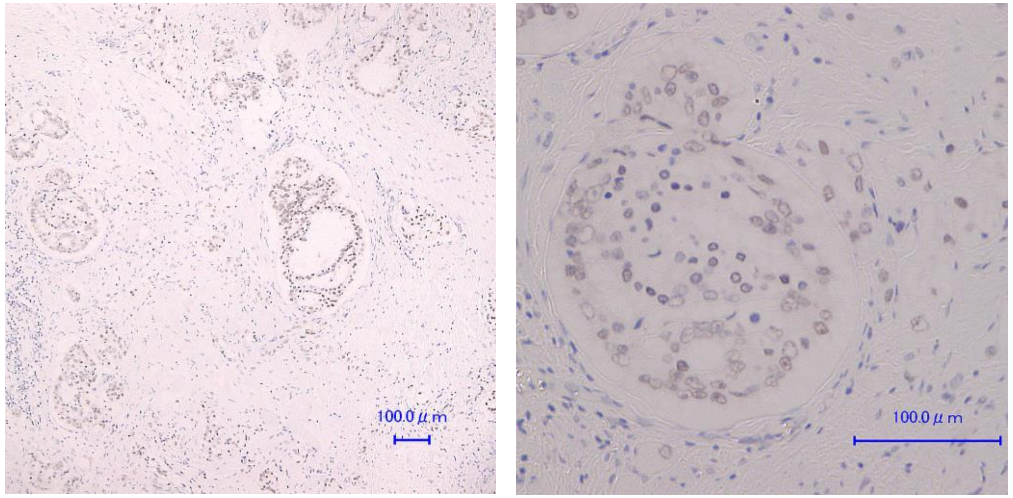

AR immunostaining in HNPC state, TS: 3 = IS: 1 + PS: 2.

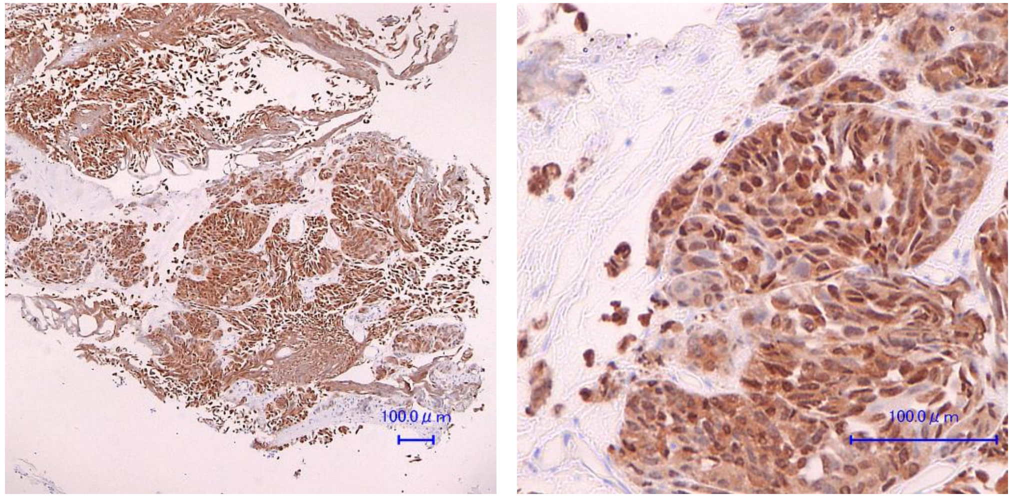

AR immunostaining in CRPC state, TS: 7 = IS: 3 + PS: 4.

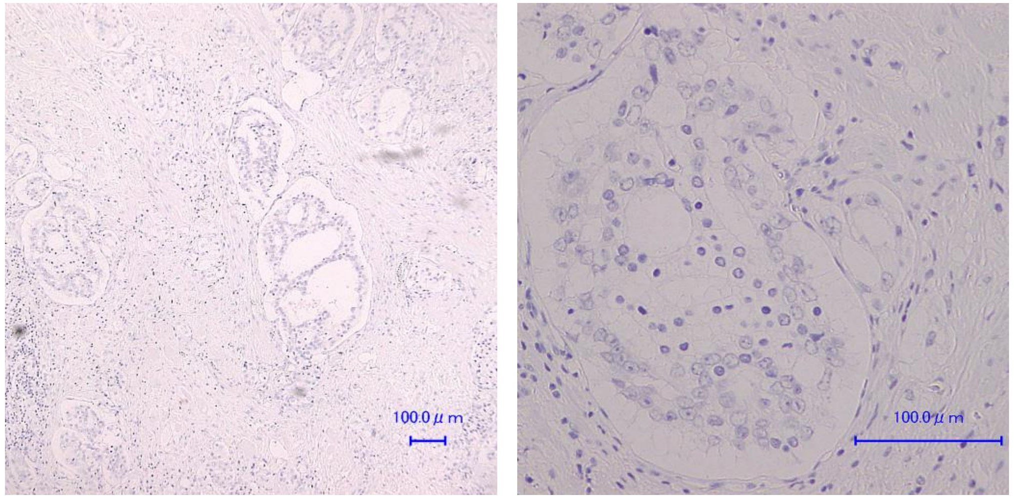

SLPI immunostaining in HNPC state, TS: 0 = IS: 0 + PS: 0.

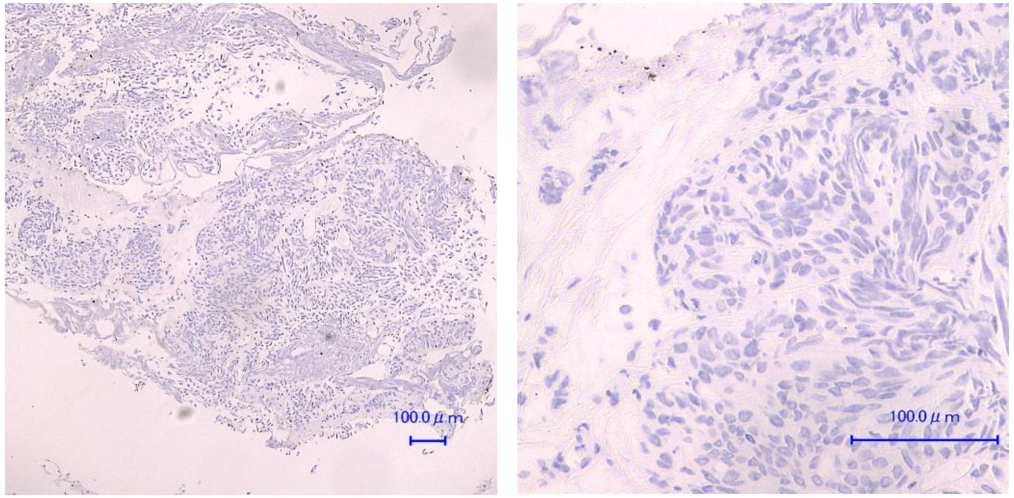

SLPI immunostaining in CRPC state, TS: 0 = IS: 0 + PS: 0.

Figure S4_Case 1

Case 2

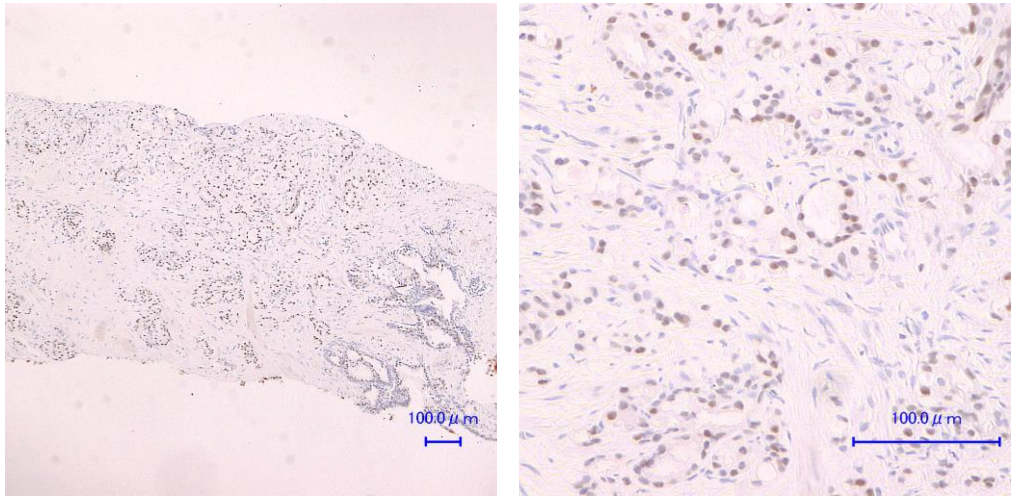

AR immunostaining in HNPC state, TS: 6 = IS: 2 + PS: 4.

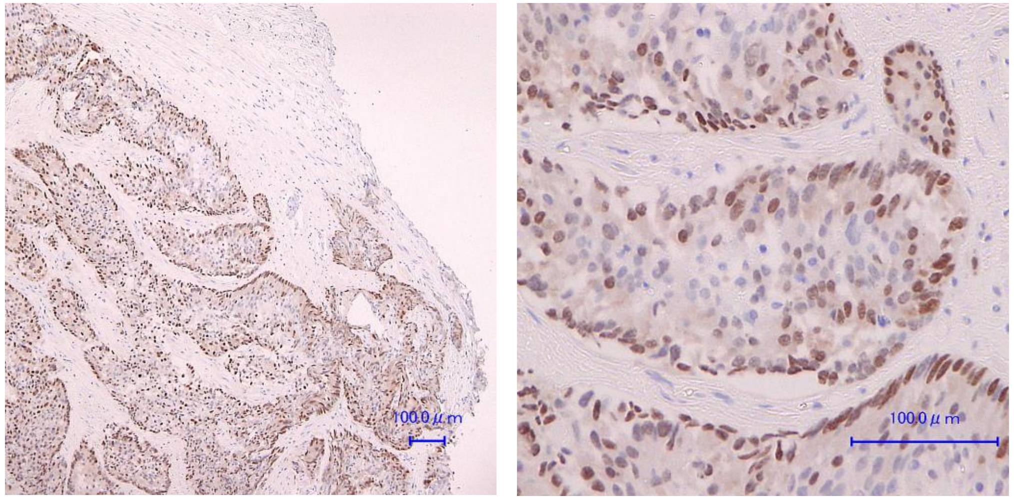

AR immunostaining in CRPC state, TS: 7 = IS: 3 + PS: 4.

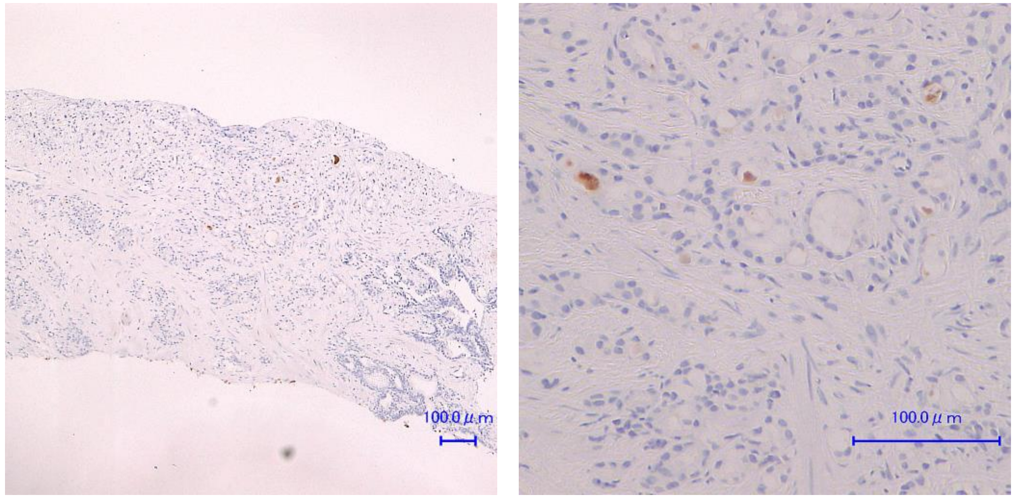

SLPI immunostaining in HNPC state, TS: 0 = IS: 0 + PS: 0.

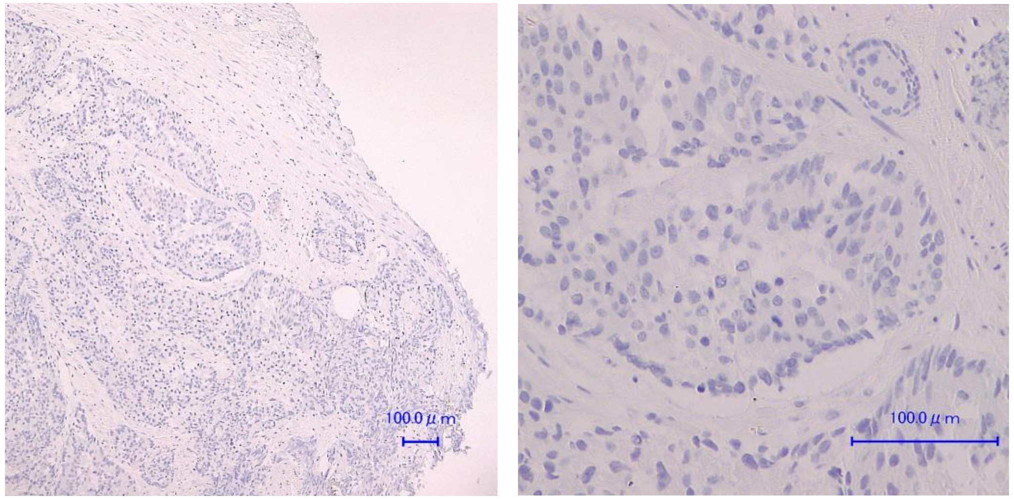

SLPI immunostaining in CRPC state, TS: 0 = IS: 0 + PS: 0.

Figure S4_Case 2

Case 3

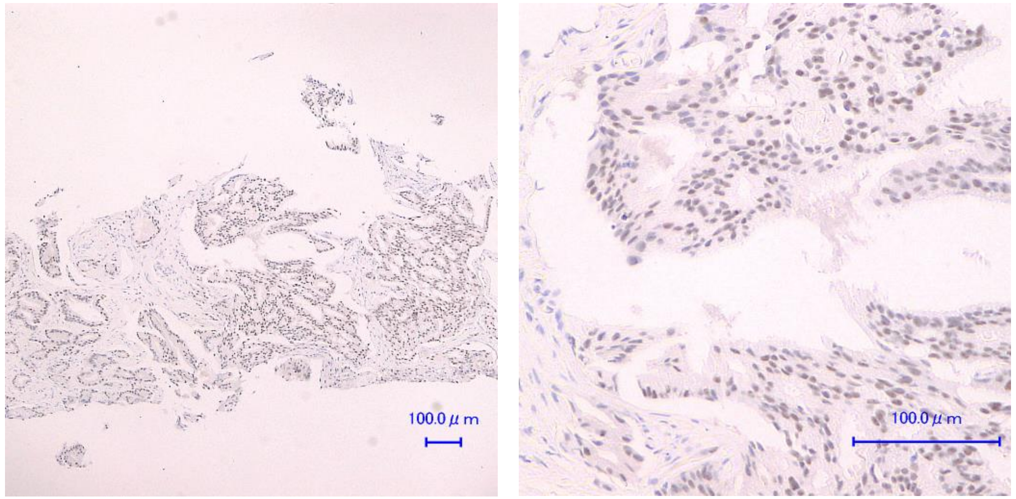

AR immunostaining in HNPC state, TS: 6 = IS: 2 + PS: 4.

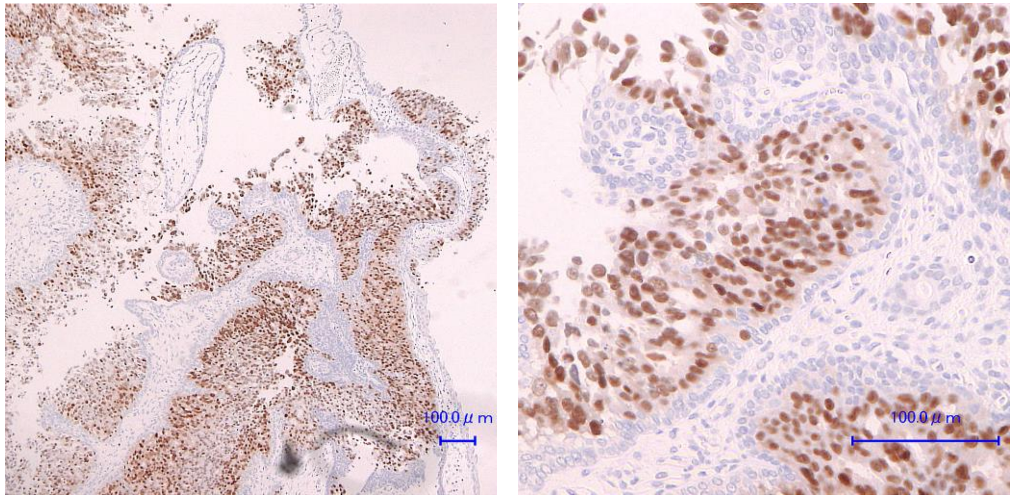

AR immunostaining in CRPC state, TS: 7 = IS: 3 + PS: 4.

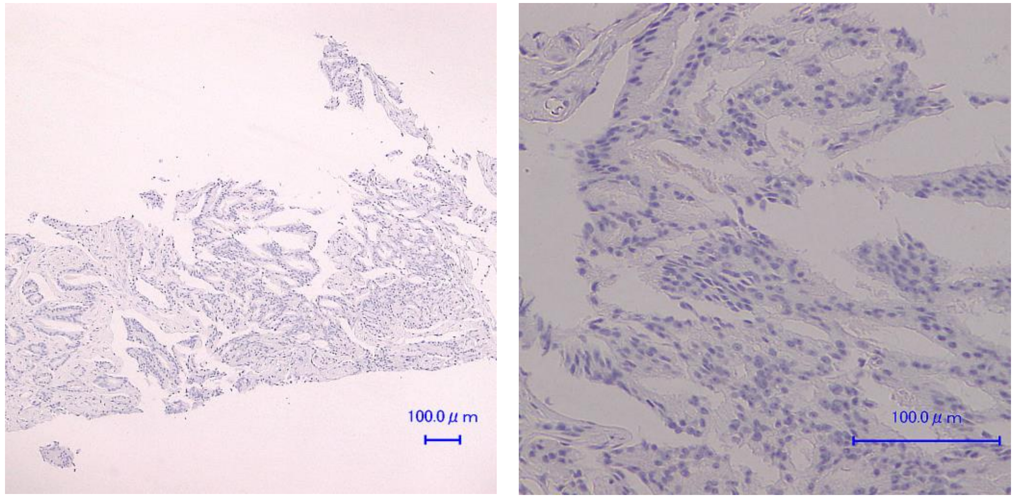

SLPI immunostaining in HNPC state, TS: 0 = IS: 0 + PS: 0.

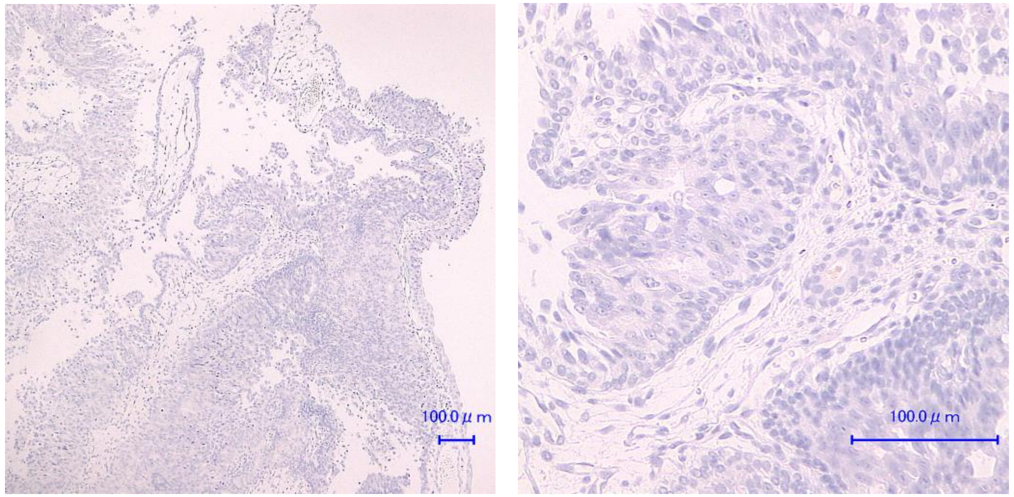

SLPI immunostaining in CRPC state, TS: 0 = IS: 0 + PS: 0.

Figure S4_Case 3

Case 4

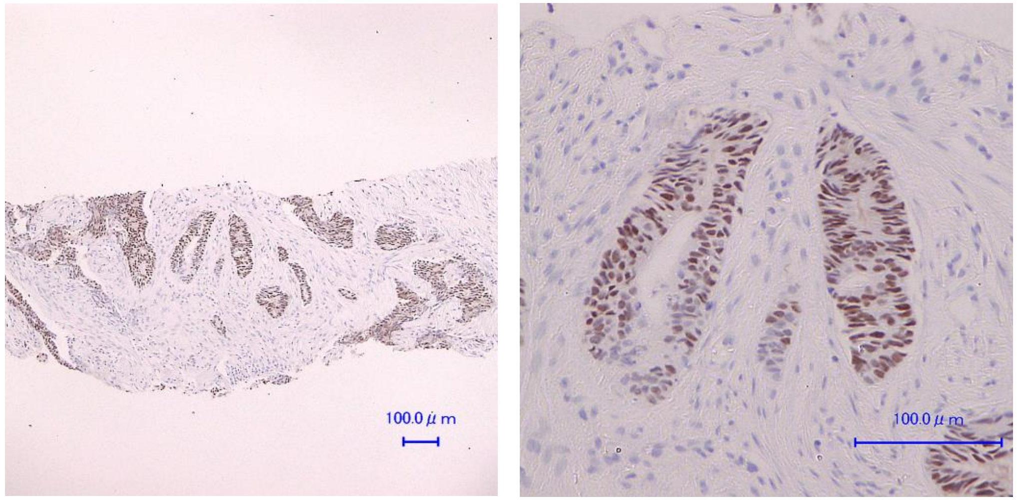

AR immunostaining in HNPC state, TS: 6 = IS: 2 + PS: 4.

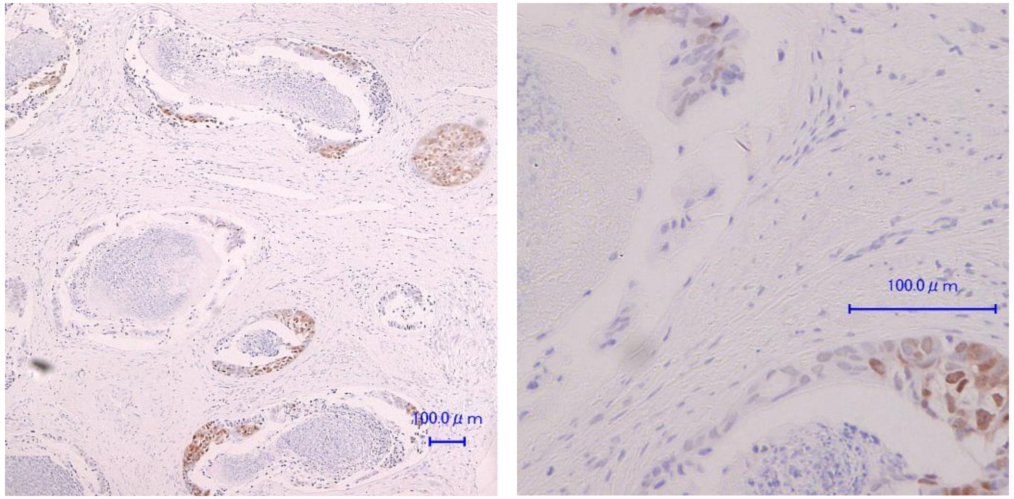

AR immunostaining in CRPC state, TS: 7 = IS: 3 + PS: 4.

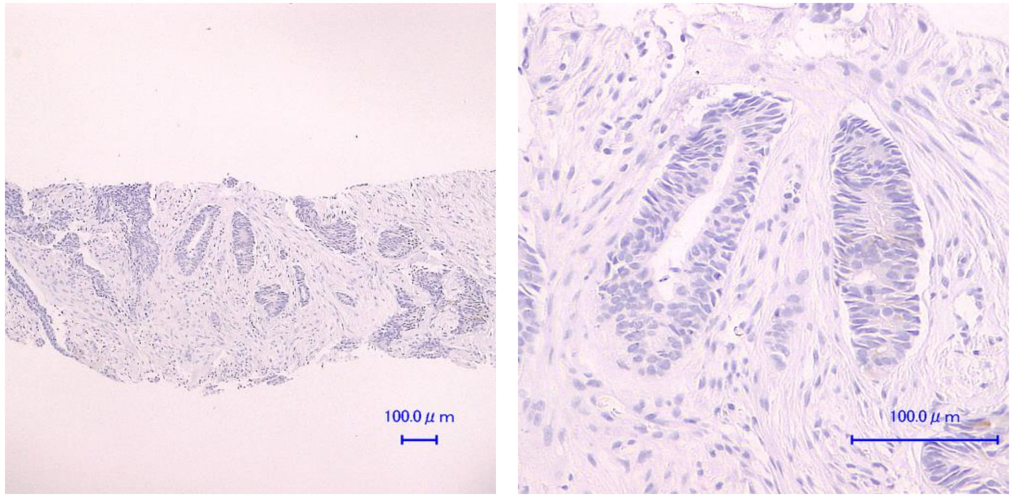

SLPI immunostaining in HNPC state, TS: 0 = IS: 0 + PS: 0.

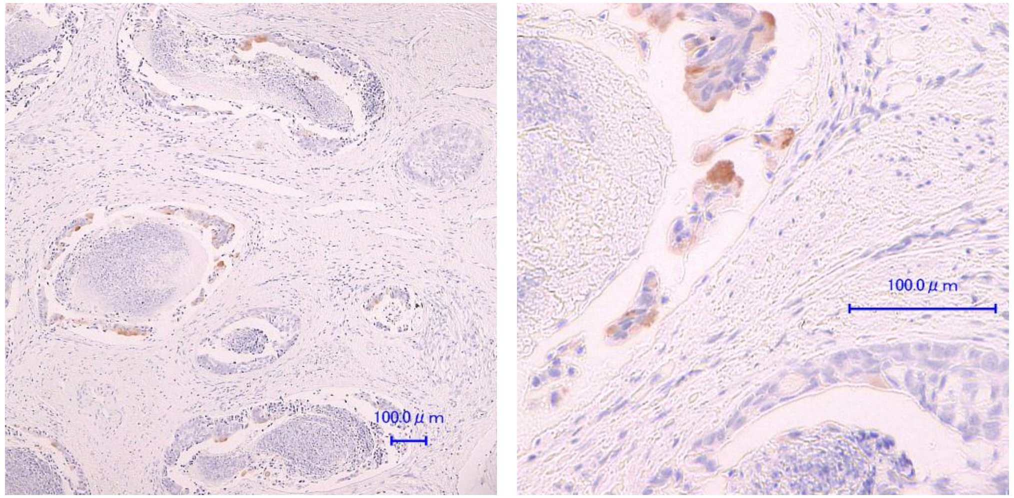

SLPI immunostaining in CRPC state, TS: 2 = IS: 1 + PS: 1.

Figure S4_Case 4

Case 5

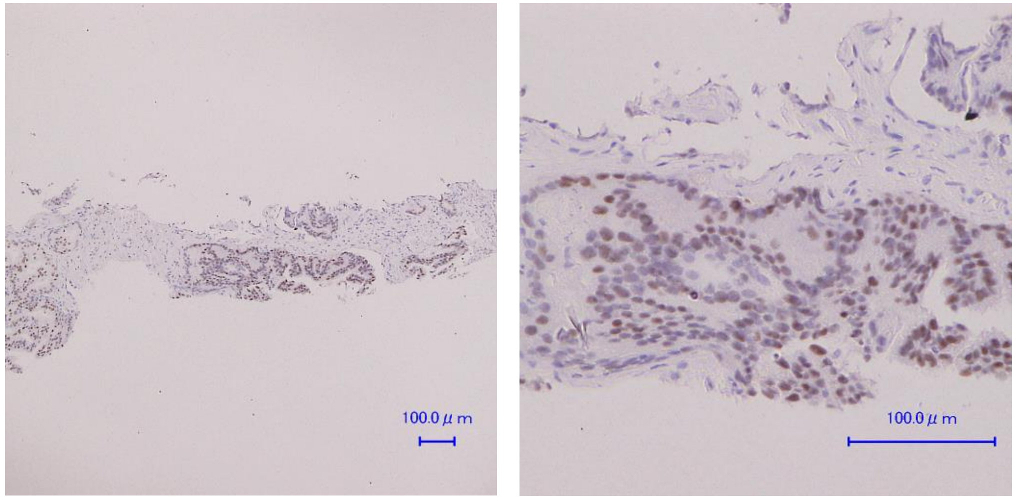

AR immunostaining in HNPC state, TS: 6 = IS: 2 + PS: 4.

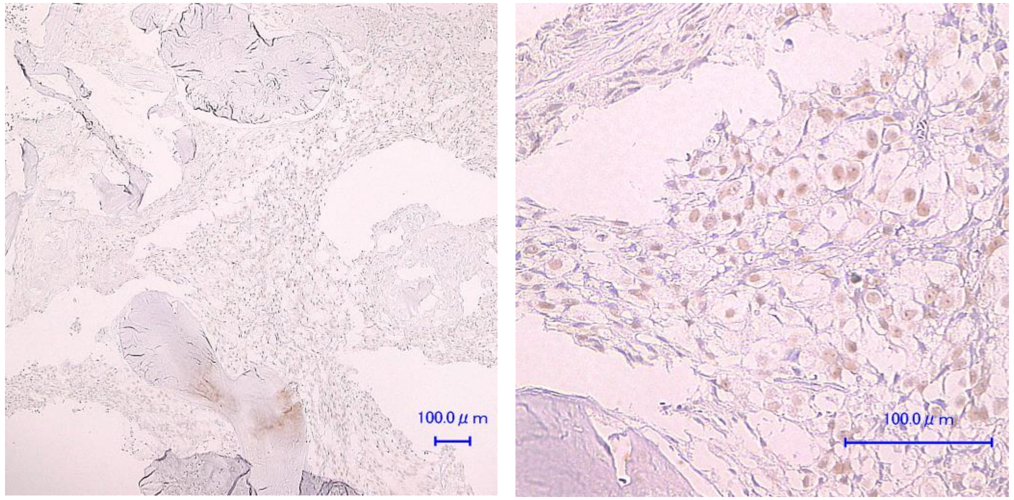

AR immunostaining in CRPC state, TS: 6 = IS: 2 + PS: 4.

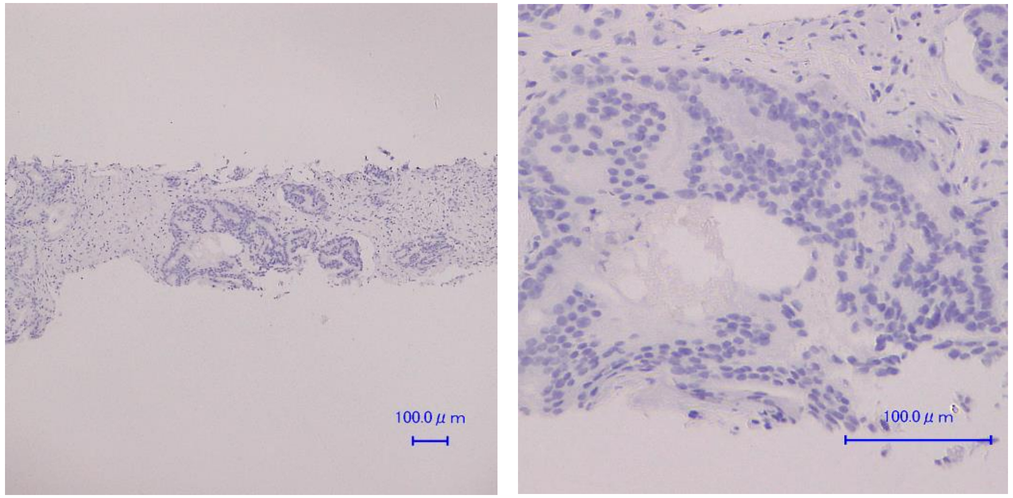

SLPI immunostaining in HNPC state, TS: 0 = IS: 0 + PS: 0.

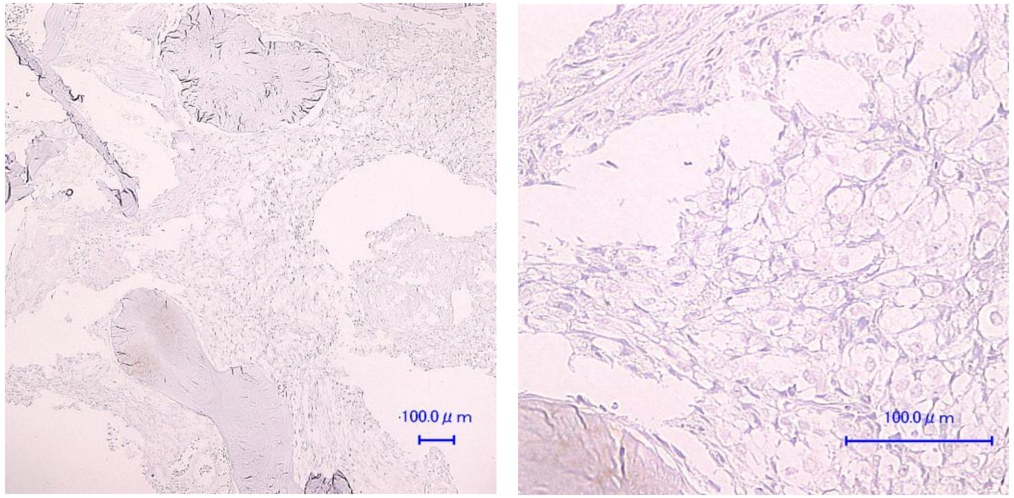

SLPI immunostaining in CRPC state, TS: 0 = IS: 0 + PS: 0.

Figure S4_Case 5

Case 6

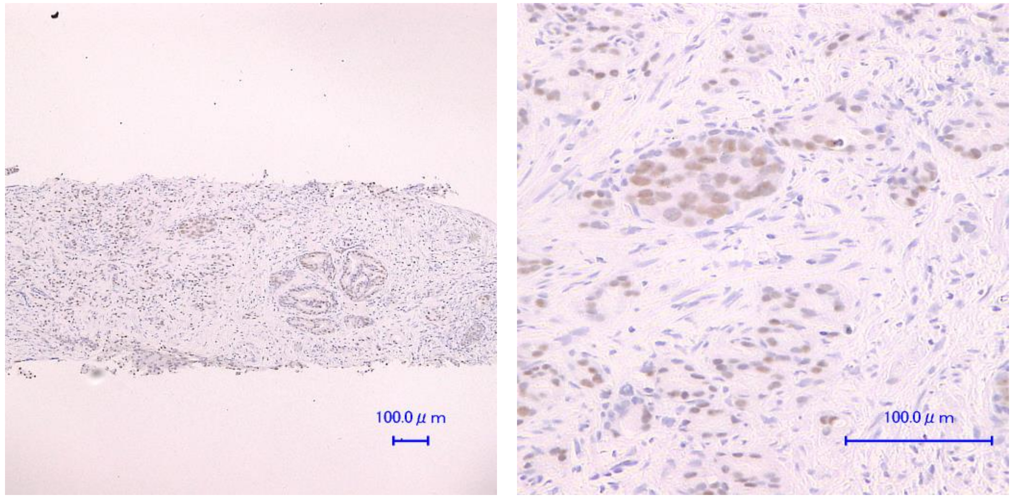

AR immunostaining in HNPC state, TS: 6 = IS: 2 + PS: 4.

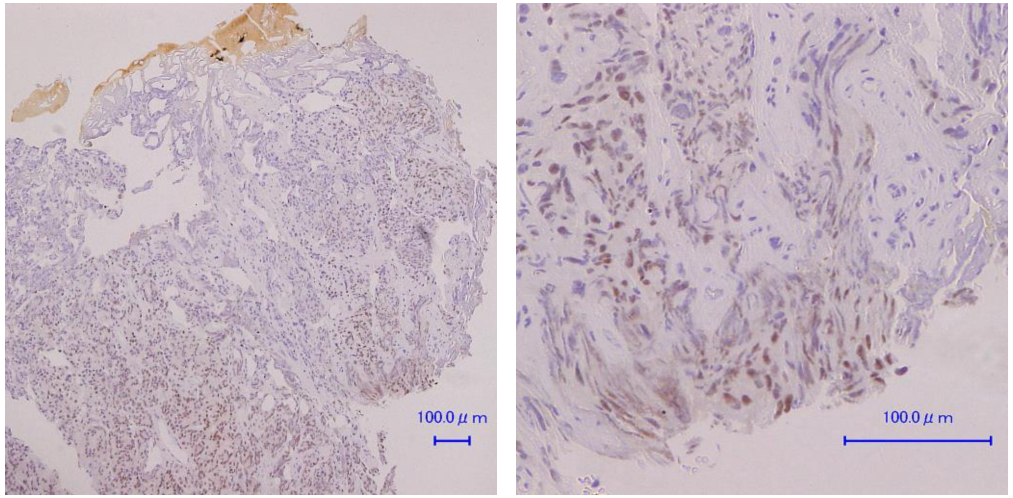

AR immunostaining in CRPC state, TS: 7 = IS: 3 + PS: 4.

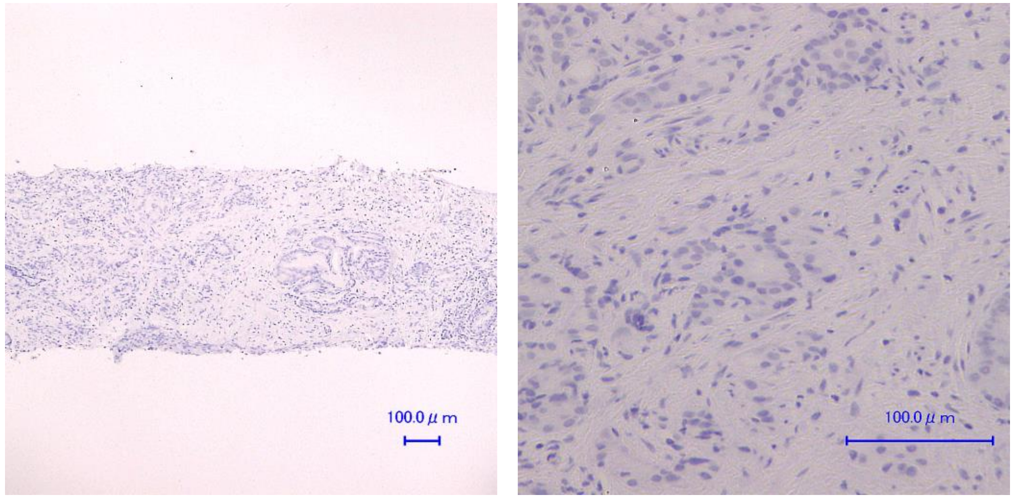

SLPI immunostaining in HNPC state, TS: 0 = IS: 0 + PS: 0.

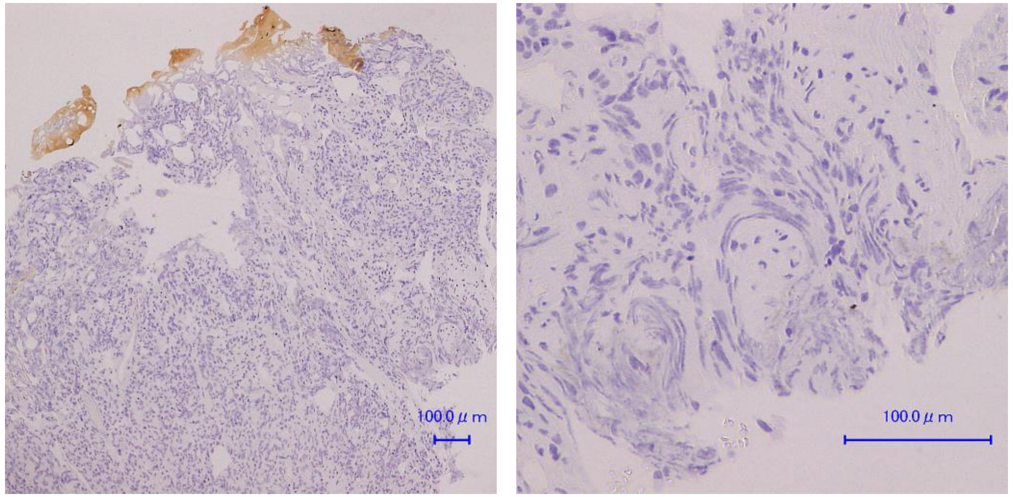

SLPI immunostaining in CRPC state, TS: 0 = IS: 0 + PS: 0.

Figure S4_Case 6

Case 7

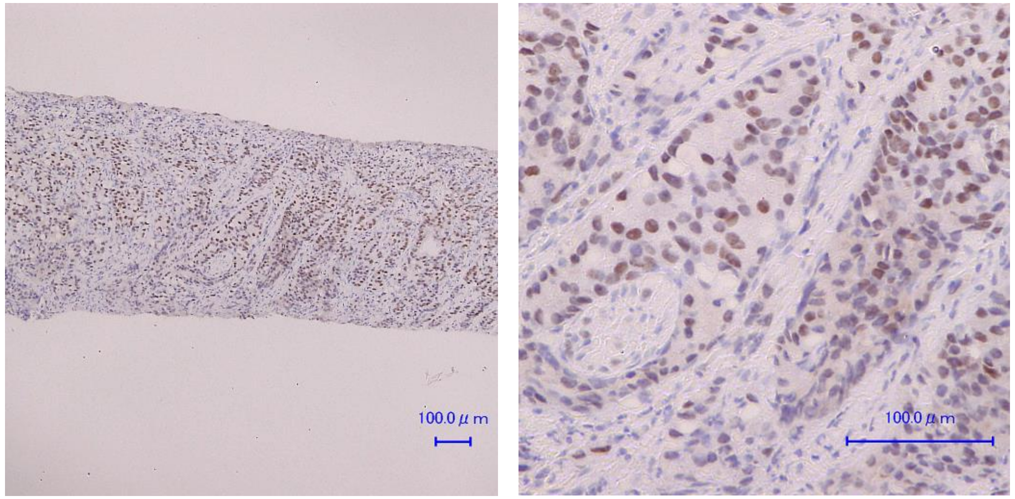

AR immunostaining in HNPC state, TS: 6 = IS: 2 + PS: 4.

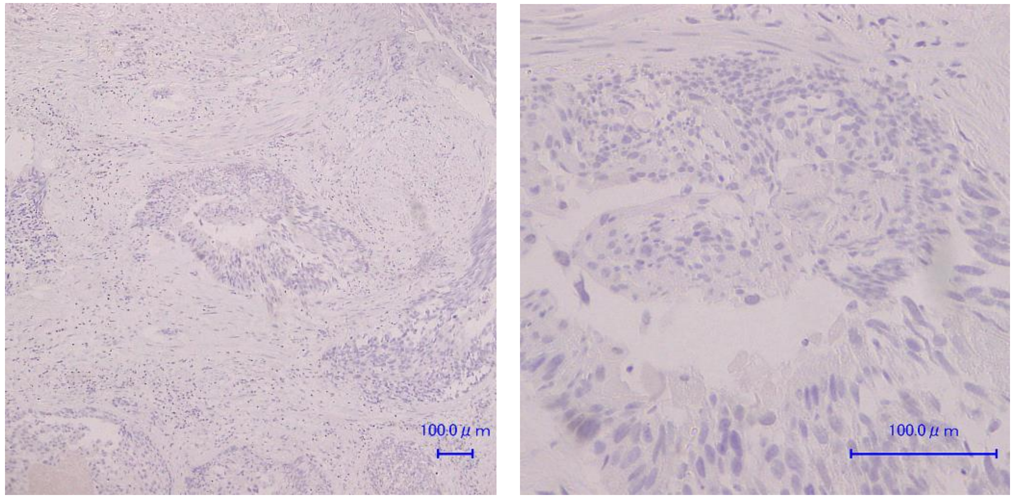

AR immunostaining in CRPC state, TS: 2 = IS: 1 + PS: 1.

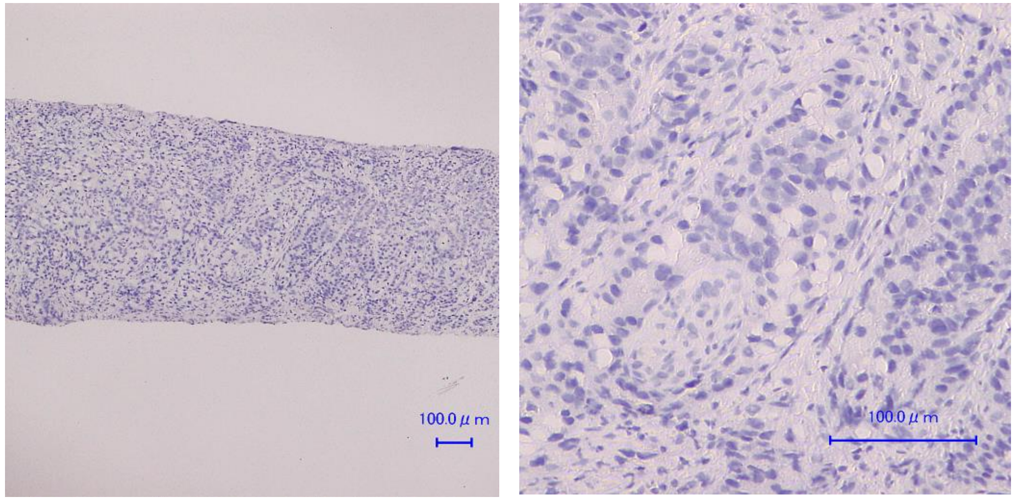

SLPI immunostaining in HNPC state, TS: 0 = IS: 0 + PS: 0.

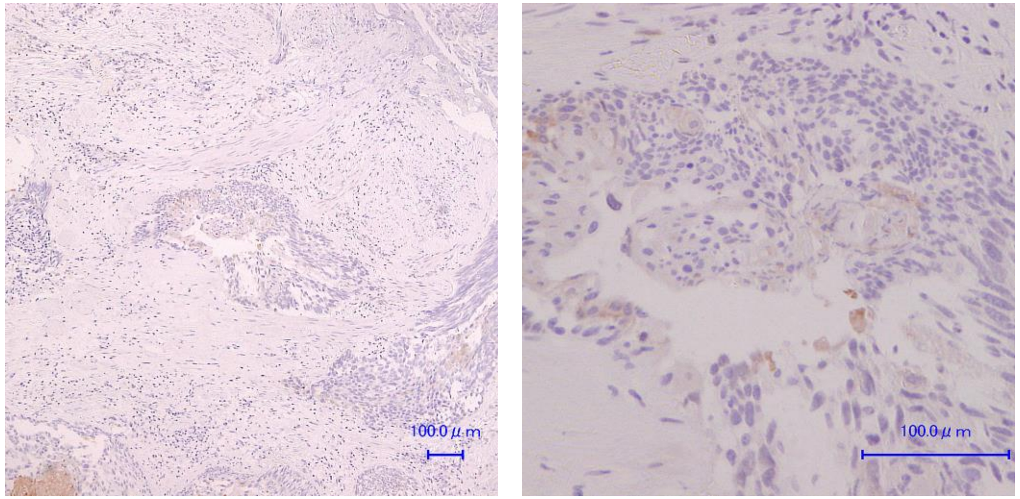

SLPI immunostaining in CRPC state, TS: 3 = IS: 2 + PS: 1.

Figure S4_Case 7

Case 8

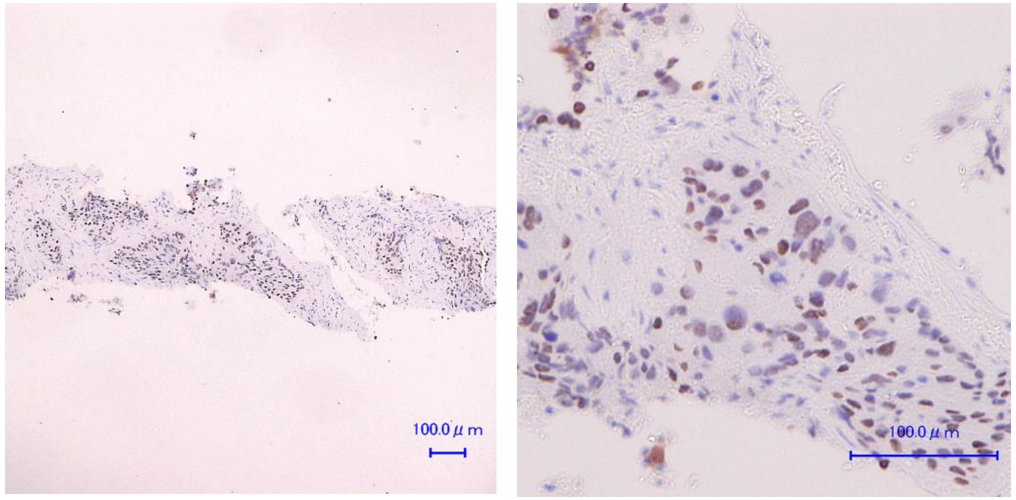

AR immunostaining in HNPC state, TS: 6 = IS: 2 + PS: 4.

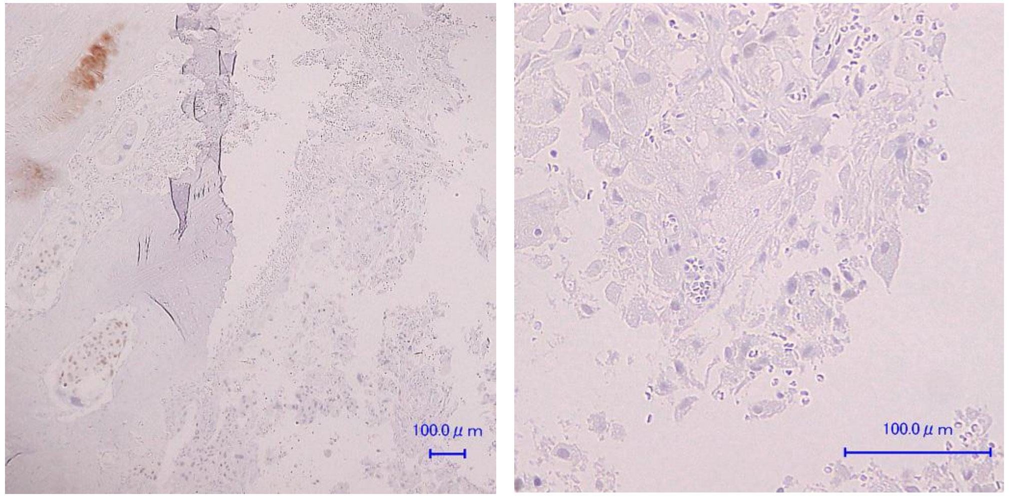

AR immunostaining in CRPC state, TS: 4 = IS: 1 + PS: 3.

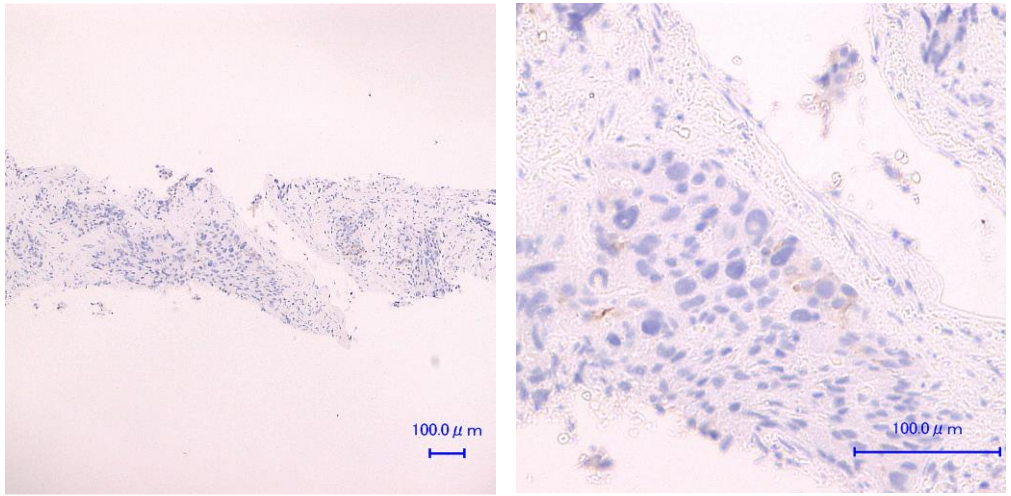

SLPI immunostaining in HNPC state, TS: 0 = IS: 0 + PS: 0.

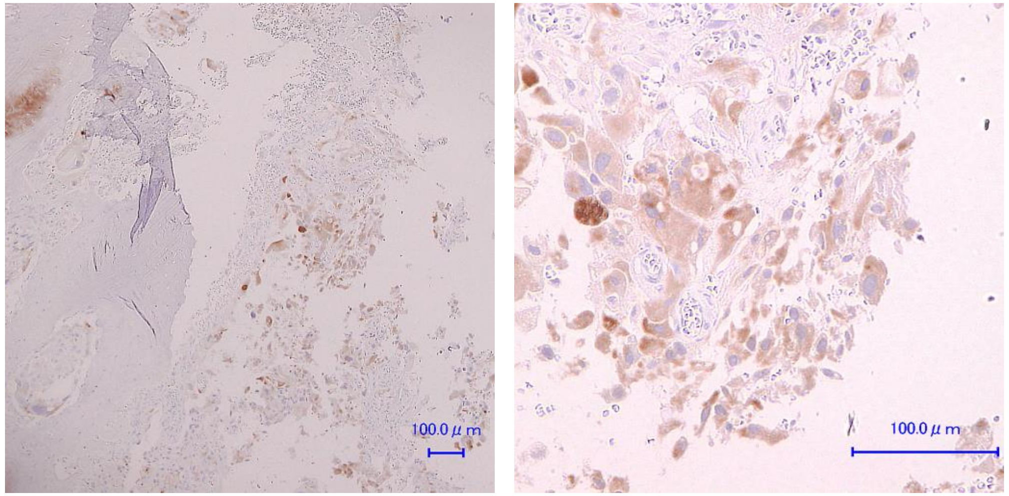

SLPI immunostaining in CRPC state, TS: 5 = IS: 2 + PS: 3.

Figure S4_Case 8

Case 9

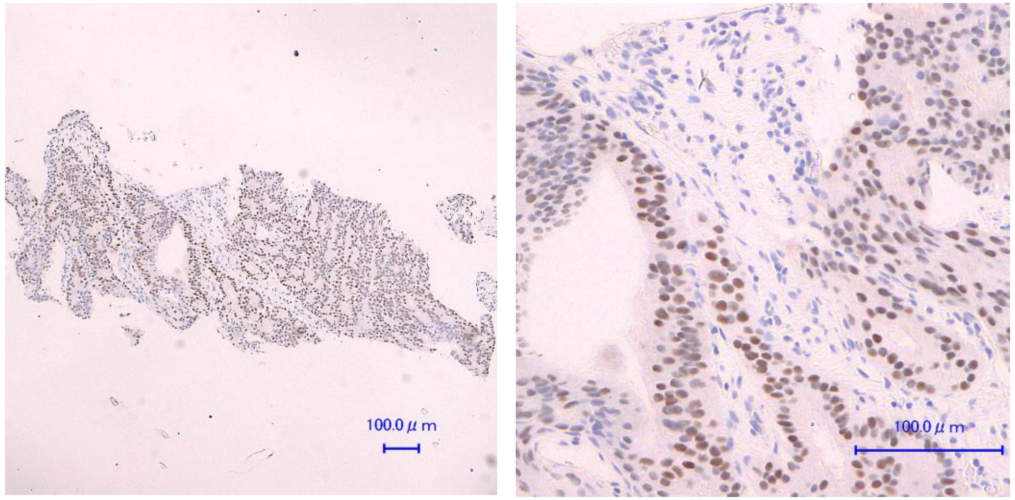

AR immunostaining in HNPC state, TS: 6 = IS: 2 + PS: 4.

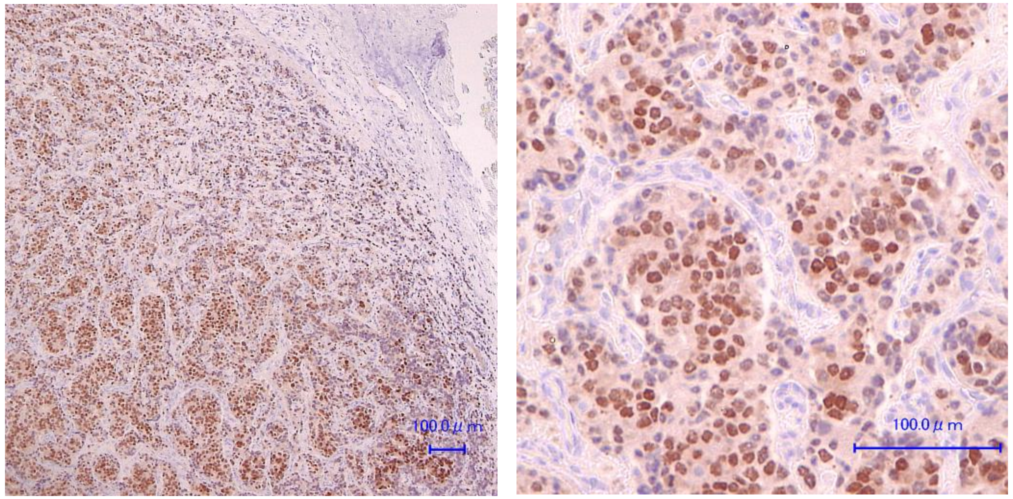

AR immunostaining in CRPC state, TS: 6 = IS: 2 + PS: 4.

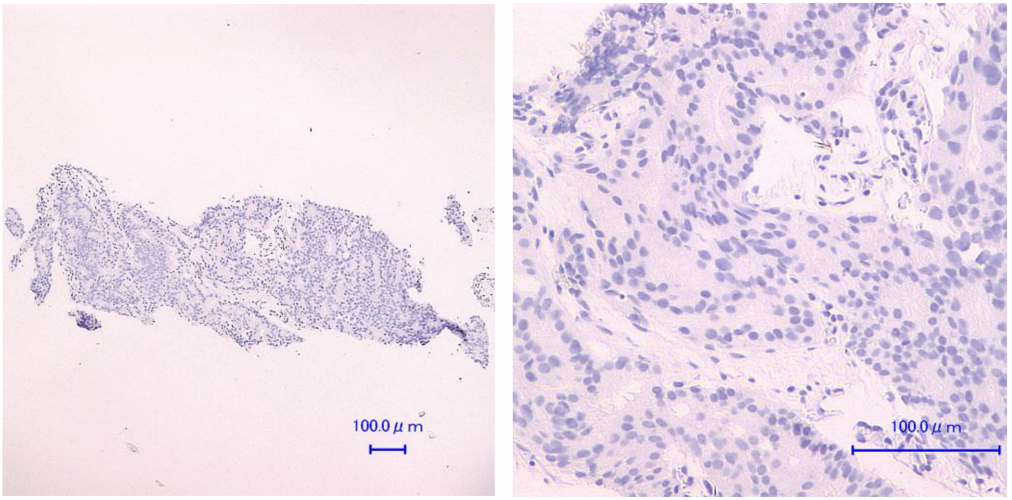

SLPI immunostaining in HNPC state, TS: 0 = IS: 0 + PS: 0.

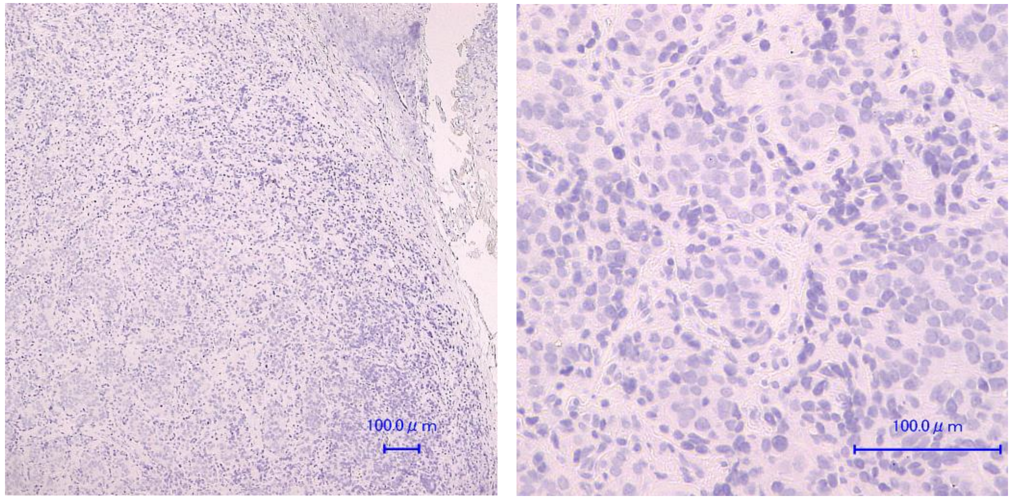

SLPI immunostaining in CRPC state, TS: 0 = IS: 0 + PS: 0.

Figure S4_Case 9

Case 10

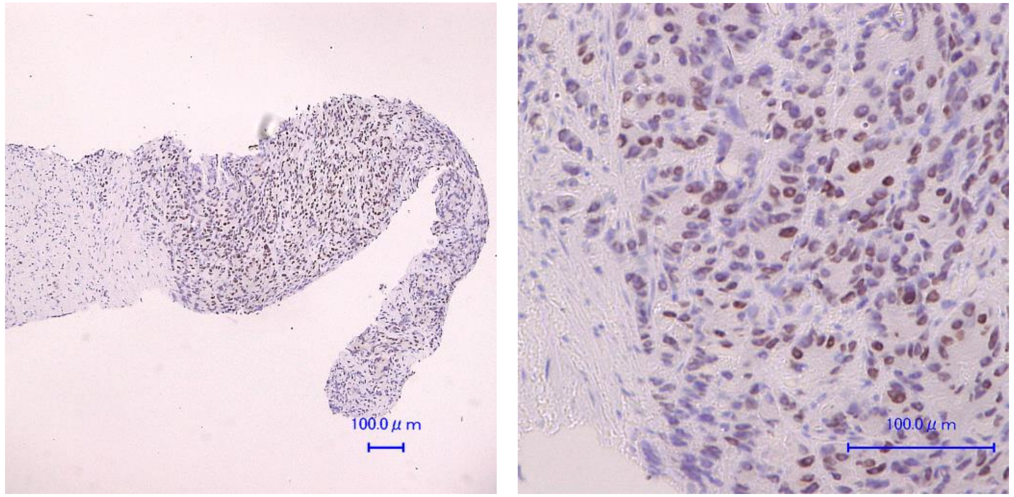

AR immunostaining in HNPC state, TS: 7 = IS: 3 + PS: 4.

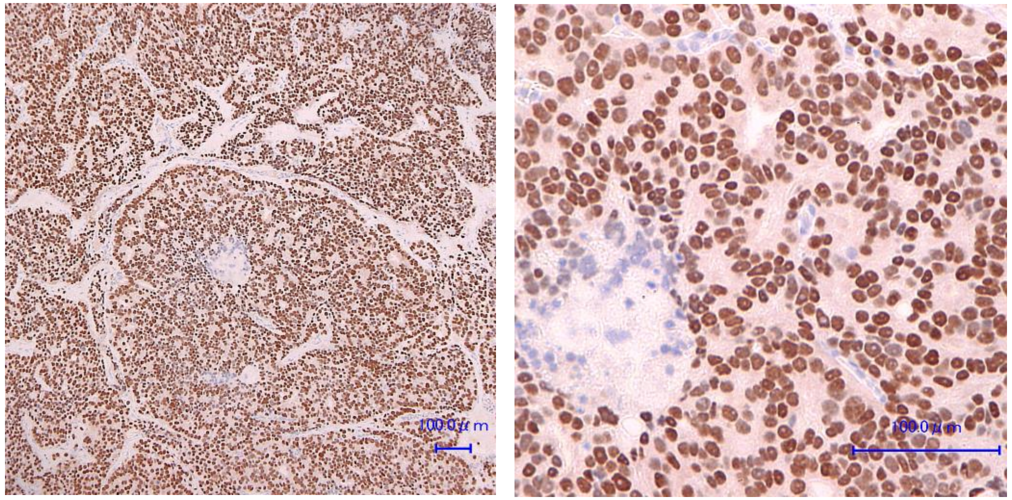

AR immunostaining in CRPC state, TS: 7 = IS: 3 + PS: 4.

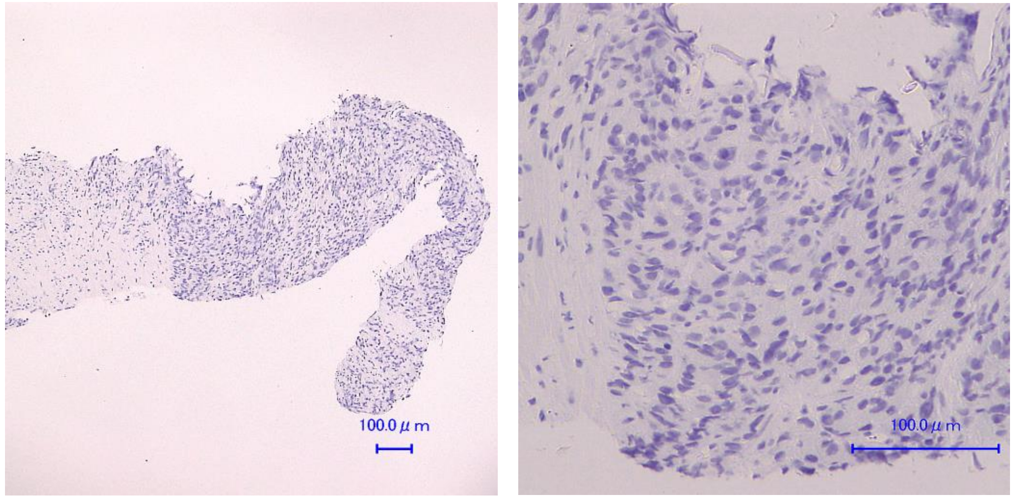

SLPI immunostaining in HNPC state, TS: 0 = IS: 0 + PS: 0.

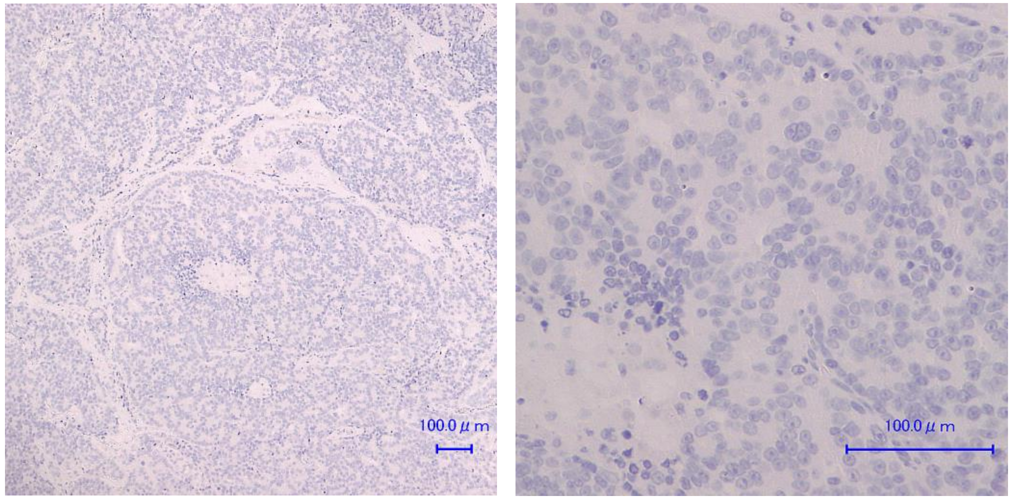

SLPI immunostaining in CRPC state, TS: 0 = IS: 0 + PS: 0.

Figure S4_Case 10

Case 11

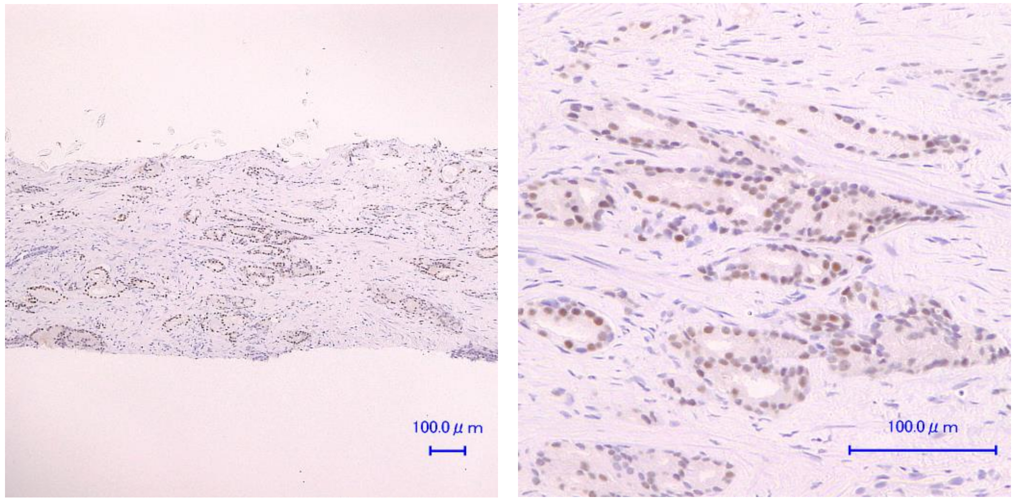

AR immunostaining in HNPC state, TS: 6 = IS: 2 + PS: 4.

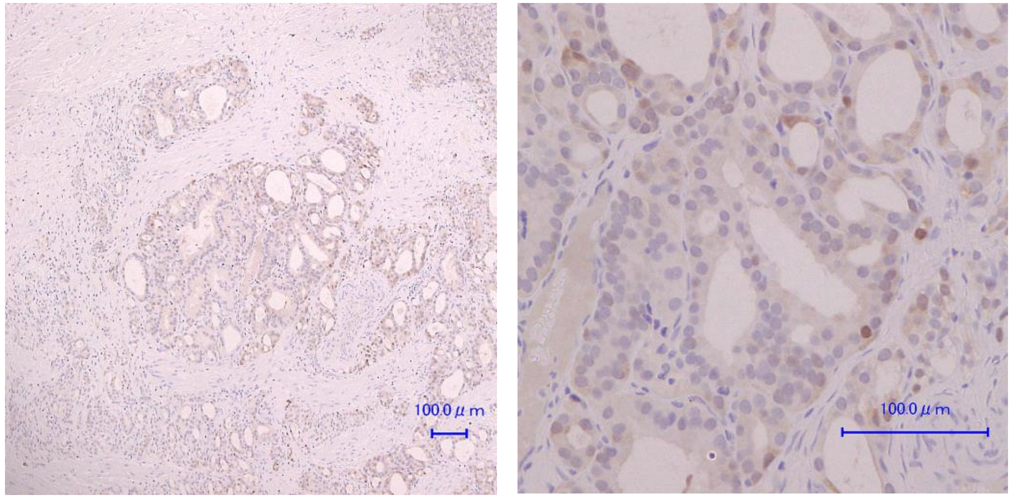

AR immunostaining in CRPC state, TS: 4 = IS: 1 + PS: 3.

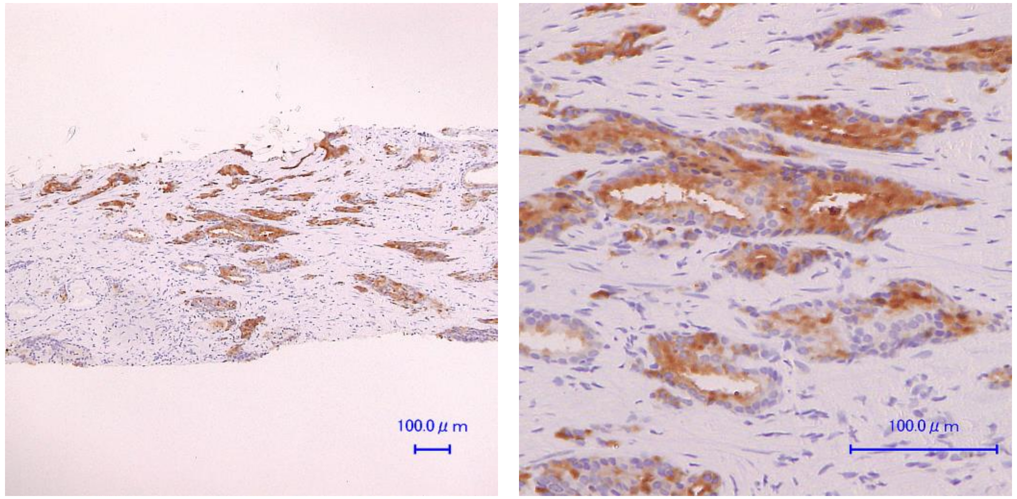

SLPI immunostaining in HNPC state, TS: 6 = IS: 3 + PS: 3.

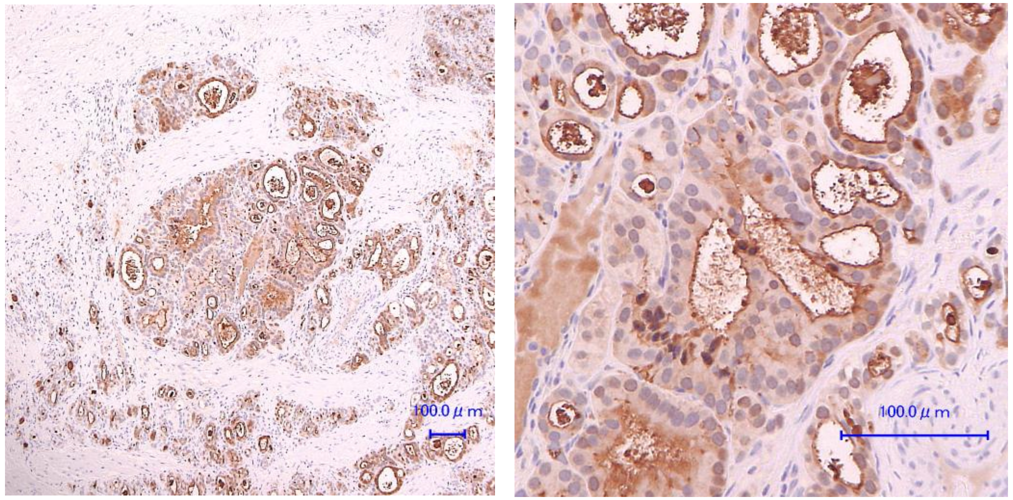

SLPI immunostaining in CRPC state, TS: 7 = IS: 3 + PS: 4.

Figure S4_Case 11

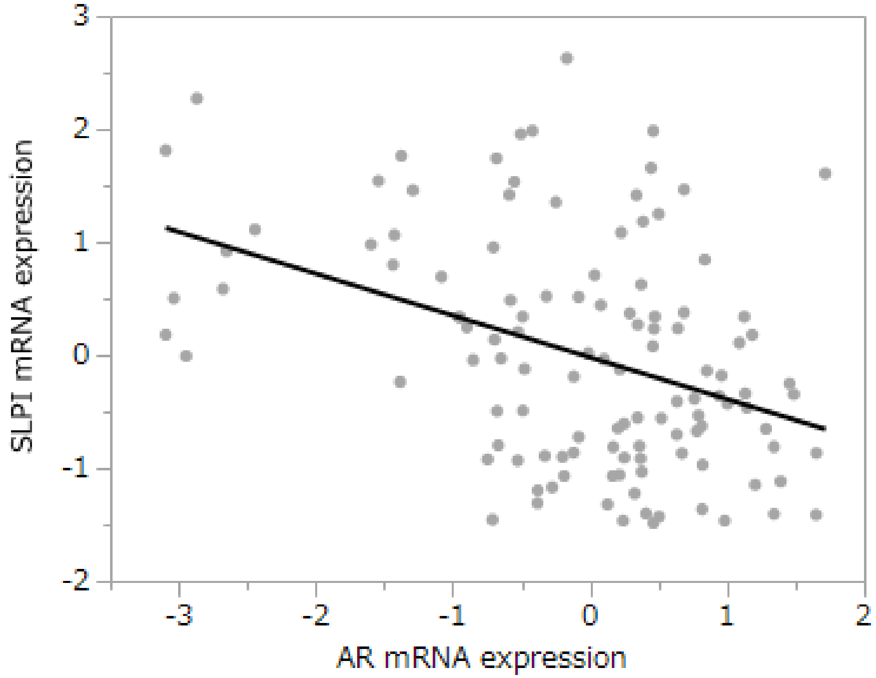

Figure S5
