# Supplementary material for: Up‐regulation of secretory leukocyte protease inhibitor in human samples might have a potential role of predicting prostate cancer recurrence and progression after surgery and hormonal therapy
Source: Cancer Med. 2022 Aug 13;12(3):3328–42. doi: 10.1002/cam4.5134 (PMC9939162; doi:10.1002/cam4.5134)
Supplement: Supplementary file 3 — Table S1 [file CAM4-12-3328-s001.pdf]

Table S1. Results of Tandem Mass Tag (TMT) analysis

Accession	Description	Score	Coverage	# Peptides	AILNCaP14 / LNCaP	AILNCaP14 / LNCaP Count	AILNCaP14 / LNCaP Variability [%]	AILNCaP15 / LNCaP	AILNCaP15 / LNCaP Count	AILNCaP15 / LNCaP Variability [%]	AILNCaP15 / AILNCaP14	AILNCaP15 / AILNCaP14 Count	AILNCaP15 / AILNCaP14 Variability [%]	# AAs	MW [kDa]	calc. pI
P10809	60 kDa heat shock protein, mitochondrial OS=Homo sapiens GN=H	4494.80	62.48	31	0.977	293	16.2	0.870	293	19.1	0.872	293	19.4	573	61.0	5.87
P16104	Histone H2AX OS=Homo sapiens GN=H2AFX PE=1 SV=2 - [H2AX_	4248.95	63.64	7	0.830	2	18.8	0.948	2	97.6	1.142	2	70.0	143	15.1	10.74
Q16777	Histone H2A type 2-C OS=Homo sapiens GN=HIST2H2AC PE=1 SV	4159.54	60.47	7	0.916	72	25.3	0.454	59	157.0	0.409	59	154.4	129	14.0	10.90
Q8IUE6	Histone H2A type 2-B OS=Homo sapiens GN=HIST2H2AB PE=1 SV	4120.78	63.08	7	0.938	6	2.2	0.404	6	190.9	0.413	6	117.8	130	14.0	10.89
P60174	Triosephosphate isomerase OS=Homo sapiens GN=TPI1 PE=1 SV	3143.56	73.43	17	0.677	242	21.0	1.282	243	16.3	1.943	242	32.4	286	30.8	5.92
P06733	Alpha-enolase OS=Homo sapiens GN=ENO1 PE=1 SV=2 - [ENOA_	2901.32	74.19	26	0.985	290	14.1	1.655	290	28.4	1.640	290	21.1	434	47.1	7.39
P08107	Heat shock 70 kDa protein 1A/1B OS=Homo sapiens GN=HSPA1A	2726.26	73.32	34	0.929	243	12.7	2.395	246	45.5	2.565	243	42.1	641	70.0	5.66
P06744	Glucose-6-phosphate isomerase OS=Homo sapiens GN=GPI PE=1	2652.56	70.43	36	0.846	251	15.7	1.392	253	33.2	1.678	251	30.1	558	63.1	8.32
P11142	Heat shock cognate 71 kDa protein OS=Homo sapiens GN=HSPA8	2579.76	74.15	39	0.864	230	11.0	0.872	230	19.3	1.015	230	14.8	646	70.9	5.52
P08238	Heat shock protein HSP 90-beta OS=Homo sapiens GN=HSP90AB1	2471.24	70.86	48	0.820	249	12.8	0.872	248	17.9	1.075	251	16.6	724	83.2	5.03
P04406	Glyceraldehyde-3-phosphate dehydrogenase OS=Homo sapiens GN	2360.81	72.84	21	0.629	261	21.0	0.584	260	30.9	0.922	260	17.5	335	36.0	8.46
P00558	Phosphoglycerate kinase 1 OS=Homo sapiens GN=PGK1 PE=1 SV	2240.57	80.10	29	1.028	260	17.6	1.885	259	41.0	1.852	259	30.6	417	44.6	8.10
P62937	Peptidyl-prolyl cis-trans isomerase A OS=Homo sapiens GN=PPIA I	2169.80	86.67	18	0.812	190	15.2	0.940	191	18.6	1.124	191	14.9	165	18.0	7.81
P12277	Creatine kinase B-type OS=Homo sapiens GN=CKB PE=1 SV=1 - [	2162.24	75.85	23	0.615	250	25.8	1.121	251	11.3	1.876	250	34.5	381	42.6	5.59
P07900	Heat shock protein HSP 90-alpha OS=Homo sapiens GN=HSP90AA	1876.32	60.66	45	0.892	118	11.9	0.651	117	34.6	0.741	117	31.3	732	84.6	5.02
P49327	Fatty acid synthase OS=Homo sapiens GN=FASN PE=1 SV=3 - [FA	1707.38	29.35	52	1.117	168	15.9	0.655	159	36.1	0.594	159	51.9	2511	273.3	6.44
P14618	Pyruvate kinase PKM OS=Homo sapiens GN=PKM PE=1 SV=4 - [K	1589.35	71.00	33	0.755	162	15.5	0.943	164	17.7	1.222	162	14.0	531	57.9	7.84
P68363	Tubulin alpha-1B chain OS=Homo sapiens GN=TUBA1B PE=1 SV=	1578.33	57.21	19										451	50.1	5.06
O60814	Histone H2B type 1-K OS=Homo sapiens GN=HIST1H2BK PE=1 SV	1526.57	68.25	12										126	13.9	10.32
P07737	Profilin-1 OS=Homo sapiens GN=PFN1 PE=1 SV=2 - [PROF1_HUM	1524.21	73.57	10	0.777	167	10.1	0.787	167	13.8	1.019	167	10.2	140	15.0	8.27
Q99880	Histone H2B type 1-L OS=Homo sapiens GN=HIST1H2BL PE=1 SV	1523.57	68.25	12	0.901	2	4.0	0.424	2	23.1	0.470	2	19.0	126	13.9	10.32
P29401	Transketolase OS=Homo sapiens GN=TKT PE=1 SV=3 - [TKT_HU	1501.59	47.19	23	1.021	134	15.0	1.017	134	20.9	0.967	134	20.4	623	67.8	7.66
Q9H773	dCTP pyrophosphatase 1 OS=Homo sapiens GN=DCTPP1 PE=1 SV	1483.03	39.41	4	0.990	42	16.2	1.201	41	22.6	1.212	41	17.4	170	18.7	5.03
P04075	Fructose-bisphosphate aldolase A OS=Homo sapiens GN=ALDOA F	1429.23	79.40	25	0.875	171	18.2	1.472	171	40.5	1.605	171	42.2	364	39.4	8.09
P0C0S5	Histone H2A.Z OS=Homo sapiens GN=H2AFZ PE=1 SV=2 - [H2AZ	1410.19	53.91	5	0.770	78	12.6	0.277	67	115.8	0.354	67	106.0	128	13.5	10.58
P06899	Histone H2B type 1-J OS=Homo sapiens GN=HIST1H2BJ PE=1 SV	1401.39	68.25	12	1.038	15	4.5	0.390	15	80.3	0.399	15	63.9	126	13.9	10.32
Q99574	Neuroserpin OS=Homo sapiens GN=SERPINI1 PE=1 SV=1 - [NEU	1394.86	61.71	22	3.301	221	113.3	5.223	221	135.9	1.534	253	15.0	410	46.4	4.91
P02751	Fibronectin OS=Homo sapiens GN=FN1 PE=1 SV=4 - [FINC_HUM	1393.05	33.03	56	2.022	241	77.1	2.710	241	90.5	1.326	250	13.7	2386	262.5	5.71
Q9BQE3	Tubulin alpha-1C chain OS=Homo sapiens GN=TUBA1C PE=1 SV=	1338.74	48.33	17	0.724	6	7.4	0.483	6	21.7	0.679	6	15.6	449	49.9	5.10
P22626	Heterogeneous nuclear ribonucleoproteins A2/B1 OS=Homo sapien	1240.22	49.01	22	0.965	117	12.5	1.051	118	16.2	1.100	117	10.9	353	37.4	8.95
P60709	Actin, cytoplasmic 1 OS=Homo sapiens GN=ACTB PE=1 SV=1 - [A	1238.54	57.87	18	1.016	56	13.3	0.640	56	36.2	0.589	56	40.0	375	41.7	5.48
P55072	Transitional endoplasmic reticulum ATPase OS=Homo sapiens GN=	1229.63	55.33	31	0.742	142	13.9	0.607	141	24.1	0.847	141	18.6	806	89.3	5.26
P00338	L-lactate dehydrogenase A chain OS=Homo sapiens GN=LDHA PE=	1150.95	66.27	21	0.739	127	14.1	1.522	127	25.0	2.135	127	33.2	332	36.7	8.27
P68366	Tubulin alpha-4A chain OS=Homo sapiens GN=TUBA4A PE=1 SV=	1107.86	55.58	18	1.028	12	9.2	1.118	12	18.5	1.092	12	20.4	448	49.9	5.06
Q06830	Peroxiredoxin-1 OS=Homo sapiens GN=PRDX1 PE=1 SV=1 - [PRD	1107.41	71.86	13	0.758	155	15.9	0.881	157	14.8	1.194	157	11.4	199	22.1	8.13
P07437	Tubulin beta chain OS=Homo sapiens GN=TUBB PE=1 SV=2 - [TE	1054.44	65.99	21	0.724	44	7.4	0.555	44	20.7	0.813	44	18.7	444	49.6	4.89
P14555	Phospholipase A2, membrane associated OS=Homo sapiens GN=P	1047.59	43.06	8	1.215	75	46.2	5.518	67	225.1	6.296	89	175.1	144	16.1	9.23
P06748	Nucleophosmin OS=Homo sapiens GN=NPM1 PE=1 SV=2 - [NPM_	1047.24	43.54	12	1.134	108	21.3	0.782	108	23.5	0.677	108	32.6	294	32.6	4.78
Q13228	Selenium-binding protein 1 OS=Homo sapiens GN=SELENBP1 PE=	1045.04	64.62	24	0.626	133	21.3	1.089	137	13.1	1.764	133	22.1	472	52.4	6.37
P09104	Gamma-enolase OS=Homo sapiens GN=ENO2 PE=1 SV=3 - [ENO	1032.65	44.24	15	0.880	27	14.5	1.968	27	41.8	2.144	27	43.3	434	47.2	5.03
P62805	Histone H4 OS=Homo sapiens GN=HIST1H4A PE=1 SV=2 - [H4_H	1026.63	59.22	8	0.972	123	10.1	0.236	112	97.3	0.237	112	108.7	103	11.4	11.36
P32119	Peroxiredoxin-2 OS=Homo sapiens GN=PRDX2 PE=1 SV=5 - [PRD	1014.21	61.62	12	0.901	102	8.0	1.020	102	9.4	1.142	103	12.2	198	21.9	5.97
P10412	Histone H1.4 OS=Homo sapiens GN=HIST1H1E PE=1 SV=2 - [H1	994.97	33.79	11	0.605	11	14.9	1.566	11	3.8	2.756	11	7.1	219	21.9	11.03
P30041	Peroxiredoxin-6 OS=Homo sapiens GN=PRDX6 PE=1 SV=3 - [PRD	984.89	83.48	17	0.697	112	13.1	0.670	110	20.7	0.979	110	13.3	224	25.0	6.38
P61978	Heterogeneous nuclear ribonucleoprotein K OS=Homo sapiens GN	972.49	36.07	14	0.750	97	12.4	0.933	97	19.4	1.255	97	16.3	463	50.9	5.54
P16403	Histone H1.2 OS=Homo sapiens GN=HIST1H1C PE=1 SV=2 - [H1	971.95	34.74	10	0.997	8	10.7	2.085	8	17.0	2.022	8	9.5	213	21.4	10.93
P01034	Cystatin-C OS=Homo sapiens GN=CST3 PE=1 SV=1 - [CYTC_HUM	958.24	52.74	7	1.414	85	33.9	1.593	85	40.9	1.123	85	11.3	146	15.8	8.75
Q99497	Protein DJ-1 OS=Homo sapiens GN=PARK7 PE=1 SV=2 - [PARK7_	928.64	76.72	12	0.782	84	15.4	1.012	84	16.2	1.271	84	12.4	189	19.9	6.79
P63241	Eukaryotic translation initiation factor 5A-1 OS=Homo sapiens GN=	925.68	50.00	7	0.849	95	18.1	1.221	95	24.2	1.308	95	23.6	154	16.8	5.24
P63104	14-3-3 protein zeta/delta OS=Homo sapiens GN=YWHAZ PE=1 SV	903.13	68.57	19	0.939	90	10.4	1.143	90	11.4	1.227	90	14.6	245	27.7	4.79
P50395	Rab GDP dissociation inhibitor beta OS=Homo sapiens GN=GDI2 F	882.02	57.98	23	0.970	59	12.1	1.044	59	12.5	1.051	59	13.4	445	50.6	6.47
P68032	Actin, alpha cardiac muscle 1 OS=Homo sapiens GN=ACTC1 PE=1	854.26	34.75	12	0.974	1		0.915	1		0.940	1		377	42.0	5.39
P13010	X-ray repair cross-complementing protein 5 OS=Homo sapiens GN	853.73	30.60	17	0.738	88	11.5	0.791	88	16.2	1.100	88	14.6	732	82.7	5.81
P40926	Malate dehydrogenase, mitochondrial OS=Homo sapiens GN=MDH	853.25	63.31	18	1.094	142	16.4	1.242	142	18.8	1.142	142	11.1	338	35.5	8.68
P09651	Heterogeneous nuclear ribonucleoprotein A1 OS=Homo sapiens GI	819.99	46.51	17	0.787	71	9.4	0.950	71	13.5	1.219	71	11.7	372	38.7	9.13
P31946	14-3-3 protein beta/alpha OS=Homo sapiens GN=YWHAB PE=1 SV	790.31	64.63	17	0.839	68	12.8	0.974	68	15.1	1.201	68	13.4	246	28.1	4.83
P62258	14-3-3 protein epsilon OS=Homo sapiens GN=YWHAE PE=1 SV=1	789.09	61.57	18	0.819	117	14.8	0.973	117	15.3	1.216	117	13.8	255	29.2	4.74
P10599	Thioredoxin OS=Homo sapiens GN=TXN PE=1 SV=3 - [THIO_HUN	787.25	65.71	8	0.860	68	11.9	0.947	68	13.6	1.114	68	14.0	105	11.7	4.92
P26599	Polypyrimidine tract-binding protein 1 OS=Homo sapiens GN=PTB	785.42	45.95	15	0.933	72	10.0	1.191	73	15.7	1.259	72	16.1	531	57.2	9.17
P07602	Prosaposin OS=Homo sapiens GN=PSAP PE=1 SV=2 - [SAP_HUM	775.16	20.80	9	1.946	105	25.4	1.832	105	27.2	0.965	108	10.8	524	58.1	5.17
P18669	Phosphoglycerate mutase 1 OS=Homo sapiens GN=PGAM1 PE=1	767.05	67.32	15	0.916	126	14.7	1.493	129	28.0	1.584	127	27.6	254	28.8	7.18
P07237	Protein disulfide-isomerase OS=Homo sapiens GN=P4HB PE=1 SV	764.92	52.56	26	1.515	121	31.4	1.786	121	40.7	1.224	121	13.4	508	57.1	4.87
O94985	Calsyntenin-1 OS=Homo sapiens GN=CLSTN1 PE=1 SV=1 - [CSTN	752.30	25.89	21	1.495	96	30.0	2.386	96	49.1	1.605	98	18.5	981	109.7	4.91
P19338	Nucleolin OS=Homo sapiens GN=NCL PE=1 SV=3 - [NUCL_HUMA	747.30	34.37	29	0.884	148	9.5	0.805	148	18.4	0.918	148	16.0	710	76.6	4.70
P68104	Elongation factor 1-alpha 1 OS=Homo sapiens GN=EEF1A1 PE=1	742.74	46.10	16	0.602	38	14.7	0.865	38	10.7	1.465	38	17.8	462	50.1	9.01
P68371	Tubulin beta-4B chain OS=Homo sapiens GN=TUBB4B PE=1 SV=1	710.20	55.96	19	0.827	12	10.2	0.643	12	15.8	0.777	12	28.3	445	49.8	4.89
P61604	10 kDa heat shock protein, mitochondrial OS=Homo sapiens GN=H	707.73	82.35	12	1.287	134	15.2	1.338	134	15.5	1.079	134	12.2	102	10.9	8.92
P12532	Creatine kinase U-type, mitochondrial OS=Homo sapiens GN=CKM	695.85	45.80	16	0.951	72	25.4	0.926	72	22.3	0.909	72	25.8	417	47.0	8.34
Q99988	Growth/differentiation factor 15 OS=Homo sapiens GN=GDF15 PE	690.20	53.90	12	1.466	74	35.1	2.158	75	40.9	1.425	77	17.8	308	34.1	9.66
Q00610	Clathrin heavy chain 1 OS=Homo sapiens GN=CLTC PE=1 SV=5 -	686.81	24.06	28	0.934	68	10.8	0.740	69	25.2	0.826	68	28.9	1675	191.5	5.69
P31150	Rab GDP dissociation inhibitor alpha OS=Homo sapiens GN=GDI1	681.37	42.06	16	0.794	16	11.9	1.043	16	17.8	1.246	16	16.4	447	50.6	5.14
P11021	78 kDa glucose-regulated protein OS=Homo sapiens GN=HSPA5 P	652.36	49.24	26	1.037	86	14.1	1.360	86	19.7	1.272	86	10.8	654	72.3	5.16
Q12906	Interleukin enhancer-binding factor 3 OS=Homo sapiens GN=ILF3	637.94	21.36	17	0.866	82	12.5	0.930	82	14.8	1.077	82	13.8	894	95.3	8.76
O43707	Alpha-actinin-4 OS=Homo sapiens GN=ACTN4 PE=1 SV=2 - [ACT	621.34	43.91	34	0.933	44	11.4	1.110	44	15.1	1.189	44	9.0	911	104.8	5.44
O75367	Core histone macro-H2A.1 OS=Homo sapiens GN=H2AFY PE=1 SV	609.94	27.96	6	1.136	23	16.2	0.505	18	24.8	0.436	18	27.7	372	39.6	9.79
P27797	Calreticulin OS=Homo sapiens GN=CALR PE=1 SV=1 - [CALR_HUN	607.74	61.87	19	1.137	98	12.6	1.655	98	21.1	1.436	98	14.9	417	48.1	4.44
P07288	Prostate-specific antigen OS=Homo sapiens GN=KLK3 PE=1 SV=2	604.82	52.11	9	1.849	59	99.3	2.533	59	132.2	1.507	62	20.3	261	28.7	7.68
P07910	Heterogeneous nuclear ribonucleoproteins C1/C2 OS=Homo sapier	603.88	35.95	13	0.854	64	11.5	0.966	64	15.3	1.128	64	11.2	306	33.6	5.08
P61981	14-3-3 protein gamma OS=Homo sapiens GN=YWHAG PE=1 SV=	587.79	72.47	18	0.978	82	11.1	1.061	82	14.4	1.059	82	14.2	247	28.3	4.89
O75874	Isocitrate dehydrogenase [NADP] cytoplasmic OS=Homo sapiens C	574.16	61.11	21	1.259	96	10.9	0.790	96	15.8	0.658	96	25.9	414	46.6	7.01
Q05639	Elongation factor 1-alpha 2 OS=Homo sapiens GN=EEF1A2 PE=1	566.65	29.16	10	0.954	5	14.6	0.833	5	14.7	0.790	5	25.7	463	50.4	9.03
P49588	Alanine--tRNA ligase, cytoplasmic OS=Homo sapiens GN=AARS PE	546.75	28.82	19	0.732	63	12.3	0.770	63	21.7	1.018	63	14.6	968	106.7	5.53
P54652	Heat shock-related 70 kDa protein 2 OS=Homo sapiens GN=HSPA	544.31	24.73	14	0.829	7	2.0	1.048	7	11.6	1.179	7	14.8	639	70.0	5.74
P31949	Protein S100-A11 OS=Homo sapiens GN=S100A11 PE=1 SV=2 - [	528.98	71.43	5	0.639	49	11.8	0.926	49	12.6	1.493	49	8.8	105	11.7	7.12
P13639	Elongation factor 2 OS=Homo sapiens GN=EEF2 PE=1 SV=4 - [EF	522.92	39.04	27	0.811	87	10.0	0.838	87	19.5	1.029	87	18.0	858	95.3	6.83
Q06323	Proteasome activator complex subunit 1 OS=Homo sapiens GN=P	518.58	50.60	12	0.747	42	31.7	0.917	42	19.9	1.379	42	21.3	249	28.7	6.02
P05783	Keratin, type I cytoskeletal 18 OS=Homo sapiens GN=KRT18 PE=1	507.04	59.53	23	0.628	91	17.5	0.671	91	21.1	1.089	91	12.7	430	48.0	5.45
P40227	T-complex protein 1 subunit zeta OS=Homo sapiens GN=CCT6A P	504.70	11.30	5	0.597	28	14.5	0.505	28	26.3	0.786	28	14.9	531	58.0	6.68
P99999	Cytochrome c OS=Homo sapiens GN=CYCS PE=1 SV=2 - [CYC_HU	474.30	60.95	8	0.981	57	15.2	1.272	57	23.5	1.301	57	14.3	105	11.7	9.57
P23528	Cofilin-1 OS=Homo sapiens GN=CFL1 PE=1 SV=3 - [COF1_HUMA	445.74	62.05	10	0.672	86	23.0	0.883	86	19.3	1.309	86	14.3	166	18.5	8.09
P02545	Prelamin-A/C OS=Homo sapiens GN=LMNA PE=1 SV=1 - [LMNA_H	435.78	42.62	24	1.005	78	11.5	0.990	78	21.0	1.013	78	15.3	664	74.1	7.02
P23284	Peptidyl-prolyl cis-trans isomerase B OS=Homo sapiens GN=PPIB I	435.21	51.39	13	1.030	85	12.7	1.266	85	25.3	1.221	85	19.4	216	23.7	9.41
P00390	Glutathione reductase, mitochondrial OS=Homo sapiens GN=GSR	428.30	27.39	9	0.767	29	11.6	0.914	29	14.2	1.181	29	11.6	522	56.2	8.50
P54819	Adenylate kinase 2, mitochondrial OS=Homo sapiens GN=AK2 PE=	411.15	37.66	6	0.860	28	7.9	1.092	28	12.2	1.299	28	11.3	239	26.5	7.81
P30101	Protein disulfide-isomerase A3 OS=Homo sapiens GN=PDIA3 PE=1	407.80	50.30	20	1.196	75	17.7	1.783	75	27.3	1.508	75	16.2	505	56.7	6.35
P52815	39S ribosomal protein L12, mitochondrial OS=Homo sapiens GN=I	404.69	27.27	5	1.273	23	25.3	1.281	23	24.3	0.990	23	18.5	198	21.3	8.87
Q12905	Interleukin enhancer-binding factor 2 OS=Homo sapiens GN=ILF2	399.43	47.95	11	0.853	61	12.2	0.888	61	21.8	1.087	61	17.7	390	43.0	5.26
P07195	L-lactate dehydrogenase B chain OS=Homo sapiens GN=LDHB PE=	399.14	52.40	15	0.183	51	81.5	0.351	52	40.7	1.638	51	32.7	334	36.6	6.05
P09972	Fructose-bisphosphate aldolase C OS=Homo sapiens GN=ALDOC F	392.43	36.26	12	0.934	30	16.0	1.518	30	27.9	1.538	30	37.4	364	39.4	6.87
P84243	Histone H3.3 OS=Homo sapiens GN=H3F3A PE=1 SV=2 - [H33_H	391.74	54.41	8	1.192	36	14.1	0.269	16	63.4	0.211	16	78.0	136	15.3	11.27

P20618	Proteasome subunit beta type-1 OS=Homo sapiens GN=PSMB1 PE	379.35	49.38	7	0.756	24	9.5	0.908	24	11.8	1.176	24	11.7	241	26.5	8.13
Q04917	14-3-3 protein eta OS=Homo sapiens GN=YWHAH PE=1 SV=4 - [	376.09	23.98	7	0.765	5	9.6	0.881	5	6.1	1.085	5	7.2	246	28.2	4.84
P27348	14-3-3 protein theta OS=Homo sapiens GN=YWHAQ PE=1 SV=1 -	372.57	56.33	14	0.688	21	13.4	0.845	21	14.6	1.259	21	8.1	245	27.7	4.78
P30086	Phosphatidylethanolamine-binding protein 1 OS=Homo sapiens GN	372.52	83.42	13	0.720	56	22.4	0.908	56	17.7	1.237	56	18.4	187	21.0	7.53
P04792	Heat shock protein beta-1 OS=Homo sapiens GN=HSPB1 PE=1 SV	371.12	85.37	13	0.872	80	9.2	0.813	80	15.6	0.919	80	15.4	205	22.8	6.40
Q9Y617	Phosphoserine aminotransferase OS=Homo sapiens GN=PSAT1 PE	363.96	28.92	9	0.876	45	8.9	1.009	45	9.9	1.145	45	9.9	370	40.4	7.66
P17066	Heat shock 70 kDa protein 6 OS=Homo sapiens GN=HSPA6 PE=1	360.97	21.77	10	0.985	1		1.248	1		1.267	1		643	71.0	6.14
P31948	Stress-induced-phosphoprotein 1 OS=Homo sapiens GN=STIP1 PE	360.58	28.36	15	0.770	51	7.9	0.943	51	13.7	1.255	51	10.4	543	62.6	6.80
P22392	Nucleoside diphosphate kinase B OS=Homo sapiens GN=NME2 PE	360.10	71.71	11	0.871	54	13.2	0.927	54	13.9	1.122	54	12.0	152	17.3	8.41
P05455	Lupus La protein OS=Homo sapiens GN=SSB PE=1 SV=2 - [LA_HU	359.39	35.05	14	0.764	69	11.1	1.112	69	15.4	1.464	69	14.7	408	46.8	7.12
P61457	Pterin-4-alpha-carbinolamine dehydratase OS=Homo sapiens GN=	356.65	39.42	4	0.978	28	18.9	1.190	29	35.2	1.244	29	30.9	104	12.0	6.80
P05787	Keratin, type II cytoskeletal 8 OS=Homo sapiens GN=KRT8 PE=1	354.07	48.65	23	**0.608**	95	18.6	0.699	95	23.1	1.141	95	11.0	483	53.7	5.59
P38159	RNA-binding motif protein, X chromosome OS=Homo sapiens GN=	338.26	21.99	10	0.962	32	8.0	0.968	32	14.3	1.043	32	19.4	391	42.3	10.05
P30048	Thioredoxin-dependent peroxide reductase, mitochondrial OS=Hor	337.56	40.63	8	0.949	53	13.1	1.181	53	21.8	1.253	53	17.0	256	27.7	7.78
P08263	Glutathione S-transferase A1 OS=Homo sapiens GN=GSTA1 PE=1	334.87	27.48	5	1.109	18	14.2	**0.561**	18	23.6	**0.530**	18	22.1	222	25.6	8.88
P08758	Annexin A5 OS=Homo sapiens GN=ANXA5 PE=1 SV=2 - [ANXA5_	330.50	56.88	16	0.743	51	11.2	0.755	51	14.2	0.995	51	9.0	320	35.9	5.05
P37802	Transgelin-2 OS=Homo sapiens GN=TAGLN2 PE=1 SV=3 - [TAGL	327.85	68.34	11	0.725	43	9.6	0.841	43	10.8	1.160	43	11.2	199	22.4	8.25
O75369	Filamin-B OS=Homo sapiens GN=FLNB PE=1 SV=2 - [FLNB_HUMA	327.43	11.64	20	0.996	48	10.3	0.747	48	30.8	0.791	48	27.4	2602	278.0	5.73
P00505	Aspartate aminotransferase, mitochondrial OS=Homo sapiens GN=	325.92	44.65	17	1.187	62	16.2	1.012	62	13.4	0.870	62	12.2	430	47.5	9.01
Q13200	26S proteasome non-ATPase regulatory subunit 2 OS=Homo sapie	324.89	7.49	4	0.798	17	16.9	0.953	17	18.5	1.291	17	19.4	908	100.1	5.20
P18206	Vinculin OS=Homo sapiens GN=VCL PE=1 SV=4 - [VINC_HUMAN]	321.20	22.49	22	0.793	47	15.9	0.933	47	17.6	1.180	47	14.5	1134	123.7	5.66
P05388	60S acidic ribosomal protein P0 OS=Homo sapiens GN=RPLP0 PE=	320.32	45.11	10	0.789	57	8.7	0.674	57	17.3	0.858	57	19.5	317	34.3	5.97
P00441	Superoxide dismutase [Cu-Zn] OS=Homo sapiens GN=SOD1 PE=1	317.54	66.23	10	0.866	43	14.3	0.898	43	17.5	1.031	43	16.0	154	15.9	6.13
P12814	Alpha-actinin-1 OS=Homo sapiens GN=ACTN1 PE=1 SV=2 - [ACTI	317.02	21.64	17	0.794	15	13.9	1.227	15	11.6	**1.506**	15	27.8	892	103.0	5.41
Q15181	Inorganic pyrophosphatase OS=Homo sapiens GN=PPA1 PE=1 SV	316.59	59.52	12	0.878	33	9.5	0.773	33	13.2	0.875	33	13.7	289	32.6	5.86
P52209	6-phosphogluconate dehydrogenase, decarboxylating OS=Homo sa	313.76	41.82	15	1.029	51	14.2	0.964	51	22.1	0.929	52	27.5	483	53.1	7.23
P22314	Ubiquitin-like modifier-activating enzyme 1 OS=Homo sapiens GN=	310.91	26.47	20	0.721	57	26.1	0.763	57	36.5	1.035	57	14.8	1058	117.8	5.76
P37837	Transaldolase OS=Homo sapiens GN=TALDO1 PE=1 SV=2 - [TALD	308.76	36.80	13	0.764	60	15.3	0.745	60	23.4	1.006	60	14.4	337	37.5	6.81
P31943	Heterogeneous nuclear ribonucleoprotein H OS=Homo sapiens GN	308.70	42.76	15	0.852	18	11.8	1.082	18	15.8	1.238	18	15.3	449	49.2	6.30
O60506	Heterogeneous nuclear ribonucleoprotein Q OS=Homo sapiens GN	303.72	32.10	20	0.868	50	8.5	1.036	50	15.3	1.169	50	7.9	623	69.6	8.59
P84090	Enhancer of rudimentary homolog OS=Homo sapiens GN=ERH PE	303.20	69.23	5	1.081	26	14.8	0.885	26	26.3	0.839	26	22.2	104	12.3	5.92
P12956	X-ray repair cross-complementing protein 6 OS=Homo sapiens GN	302.91	27.09	13	0.748	52	14.2	0.802	52	21.3	1.108	52	13.4	609	69.8	6.64
P42126	Enoyl-CoA delta isomerase 1, mitochondrial OS=Homo sapiens GN	301.96	37.75	8	1.097	33	11.3	1.437	33	16.2	1.251	33	12.9	302	32.8	8.54
P18065	Insulin-like growth factor-binding protein 2 OS=Homo sapiens GN=	297.95	46.15	12	1.128	61	30.2	**2.184**	61	49.5	**2.099**	62	35.5	325	34.8	7.50
P0CG48	Polyubiquitin-C OS=Homo sapiens GN=UBC PE=1 SV=3 - [UBC_HI	295.82	85.40	8	0.916	56	16.5	1.209	56	14.8	1.392	56	15.2	685	77.0	7.66
P07954	Fumarate hydratase, mitochondrial OS=Homo sapiens GN=FH PE=	290.11	48.63	16	0.951	51	12.7	0.962	51	13.1	1.063	51	16.5	510	54.6	8.76
O00410	Importin-5 OS=Homo sapiens GN=IPO5 PE=1 SV=4 - [IPO5_HUM	289.96	6.38	5	0.945	17	12.7	0.972	17	8.5	1.076	17	14.1	1097	123.5	4.94
Q9C005	Protein dpy-30 homolog OS=Homo sapiens GN=DPY30 PE=1 SV=	285.97	42.42	3	0.701	25	11.5	0.916	25	9.8	1.269	25	10.4	99	11.2	4.88
O00299	Chloride intracellular channel protein 1 OS=Homo sapiens GN=CLI	282.59	56.02	9	1.003	34	12.5	0.722	34	15.1	0.769	34	21.3	241	26.9	5.17
Q7Z4W1	L-xylulose reductase OS=Homo sapiens GN=DCXR PE=1 SV=2 - [I	282.01	18.85	2	0.883	34	15.9	1.191	34	16.3	1.362	34	11.8	244	25.9	8.10
P07814	Bifunctional glutamate/proline--tRNA ligase OS=Homo sapiens GN=	281.72	3.51	5	0.783	13	33.9	0.749	12	17.3	0.886	12	22.2	1512	170.5	7.33
Q13011	Delta(3,5)-Delta(2,4)-dienoyl-CoA isomerase, mitochondrial OS=H	267.57	32.62	9	1.092	42	14.8	0.836	42	12.0	0.755	42	11.2	328	35.8	8.00
Q71DI3	Histone H3.2 OS=Homo sapiens GN=HIST2H3A PE=1 SV=3 - [H3	267.29	54.41	8	1.034	42	22.9	**0.267**	21	102.7	**0.298**	21	105.0	136	15.4	11.27
Q14974	Importin subunit beta-1 OS=Homo sapiens GN=KPNB1 PE=1 SV=	267.10	18.95	9	0.776	28	11.2	0.732	28	19.4	0.994	28	12.4	876	97.1	4.78
Q02790	Peptidyl-prolyl cis-trans isomerase FKBP4 OS=Homo sapiens GN=F	263.93	47.28	16	0.821	42	12.6	0.870	42	12.9	1.083	42	11.2	459	51.8	5.43
P26639	Threonine--tRNA ligase, cytoplasmic OS=Homo sapiens GN=TARS	263.03	23.93	16	0.872	34	14.4	0.835	34	14.7	0.955	34	16.4	723	83.4	6.67
Q00796	Sorbitol dehydrogenase OS=Homo sapiens GN=SORD PE=1 SV=4	255.90	31.09	9	0.756	35	15.1	0.811	35	34.3	0.990	35	22.6	357	38.3	7.97
P05386	60S acidic ribosomal protein P1 OS=Homo sapiens GN=RPLP1 PE=	255.15	34.21	3	0.775	10	11.1	0.675	10	15.2	0.870	10	4.4	114	11.5	4.32
P05387	60S acidic ribosomal protein P2 OS=Homo sapiens GN=RPLP2 PE=	254.31	91.30	6	0.861	38	9.1	0.849	38	10.1	1.001	38	9.6	115	11.7	4.54
O76038	Secretagogin OS=Homo sapiens GN=SCGN PE=1 SV=2 - [SEGN_H	249.01	42.03	10	**2.150**	35	64.7	**2.868**	35	67.7	1.303	38	9.3	276	32.0	5.41
O60936	Nucleolar protein 3 OS=Homo sapiens GN=NOL3 PE=1 SV=1 - [N	243.89	8.68	1	0.833	7	27.7	1.139	7	16.9	1.375	7	28.7	219	24.3	11.33
P40925	Malate dehydrogenase, cytoplasmic OS=Homo sapiens GN=MDH1	240.37	55.69	13	**0.664**	52	12.5	0.782	52	13.5	1.164	52	16.2	334	36.4	7.36
Q01518	Adenylyl cyclase-associated protein 1 OS=Homo sapiens GN=CAP1	239.27	11.58	4	0.694	26	13.2	0.938	26	12.8	1.326	26	14.4	475	51.9	8.06
P20962	Parathymosin OS=Homo sapiens GN=PTMS PE=1 SV=2 - [PTMS_H	234.97	10.78	1	0.743	16	15.2	1.399	16	11.4	**1.755**	16	23.4	102	11.5	4.16
O00391	Sulfhydryl oxidase 1 OS=Homo sapiens GN=QSOX1 PE=1 SV=3 -	232.61	16.73	11	0.950	44	14.6	**1.800**	44	50.6	**1.995**	44	37.2	747	82.5	8.92
P49720	Proteasome subunit beta type-3 OS=Homo sapiens GN=PSMB3 PE	222.04	47.80	7	0.798	16	15.5	0.975	16	38.4	1.163	16	40.3	205	22.9	6.55
P48643	T-complex protein 1 subunit epsilon OS=Homo sapiens GN=CCT5	221.04	16.45	6	0.751	14	37.5	0.842	15	39.0	0.997	14	40.6	541	59.6	5.66
P07339	Cathepsin D OS=Homo sapiens GN=CTSD PE=1 SV=1 - [CATD_HU	220.48	11.41	4	1.195	18	8.4	0.824	18	54.6	0.684	18	37.1	412	44.5	6.54
P03973	Antileukoproteinase OS=Homo sapiens GN=SLPI PE=1 SV=2 - [SL	219.07	43.18	8	**4.744**	40	101.2	**6.723**	40	103.3	**1.533**	50	8.0	132	14.3	8.75
P39687	Acidic leucine-rich nuclear phosphoprotein 32 family member A OS	218.33	51.81	12	0.746	37	16.8	1.091	37	14.5	**1.538**	37	15.9	249	28.6	4.09
P34932	Heat shock 70 kDa protein 4 OS=Homo sapiens GN=HSPA4 PE=1	217.88	28.57	19	0.897	38	11.3	0.938	38	10.5	1.050	38	9.5	840	94.3	5.19
P48163	NADP-dependent malic enzyme OS=Homo sapiens GN=ME1 PE=1	217.58	24.65	7	0.915	22	11.6	**0.557**	22	67.9	0.683	22	63.0	572	64.1	6.13
P60660	Myosin light polypeptide 6 OS=Homo sapiens GN=MYL6 PE=1 SV=	217.46	46.36	6	0.797	24	7.7	0.925	24	16.9	1.148	24	11.3	151	16.9	4.65
P52597	Heterogeneous nuclear ribonucleoprotein F OS=Homo sapiens GN	217.25	30.36	9	0.865	24	9.5	1.214	24	14.8	1.426	24	13.9	415	45.6	5.58
Q12931	Heat shock protein 75 kDa, mitochondrial OS=Homo sapiens GN=	215.32	6.96	3	1.165	6	27.4	0.977	6	17.4	0.851	6	29.3	704	80.1	8.21
P27695	DNA-(apurinic or apyrimidinic site) lyase OS=Homo sapiens GN=A	214.64	29.87	6	0.770	19	15.0	0.917	19	15.9	1.100	19	9.1	318	35.5	8.12
Q9P0M6	Core histone macro-H2A.2 OS=Homo sapiens GN=H2AFY2 PE=1 S	213.40	7.80	1	0.841	4	31.3	**0.463**	4	14.3	**0.554**	4	72.4	372	40.0	9.69
P80303	Nucleobindin-2 OS=Homo sapiens GN=NUCB2 PE=1 SV=2 - [NUC	212.98	41.19	17	1.100	43	15.9	1.318	43	17.7	1.199	43	9.7	420	50.2	5.12
Q04637	Eukaryotic translation initiation factor 4 gamma 1 OS=Homo sapie	209.18	3.63	3	0.893	13	14.6	0.984	13	15.6	1.151	13	19.3	1599	175.4	5.33
P60842	Eukaryotic initiation factor 4A-I OS=Homo sapiens GN=EIF4A1 PE	205.30	22.91	7	0.891	23	6.7	0.689	23	11.5	0.807	23	17.7	406	46.1	5.48
P04080	Cystatin-B OS=Homo sapiens GN=CSTB PE=1 SV=2 - [CYTB_HUM	204.44	61.22	5	0.881	21	25.7	1.337	21	39.6	1.245	21	20.3	98	11.1	7.56
P61204	ADP-ribosylation factor 3 OS=Homo sapiens GN=ARF3 PE=1 SV=	203.35	39.23	8	**0.445**	31	38.3	**0.584**	31	28.8	1.201	31	14.7	181	20.6	7.43
P09874	Poly [ADP-ribose] polymerase 1 OS=Homo sapiens GN=PARP1 PE	201.41	9.66	9	0.756	17	9.4	0.748	17	17.0	1.040	17	13.4	1014	113.0	8.88
Q96E39	RNA binding motif protein, X-linked-like-1 OS=Homo sapiens GN=	199.85	15.90	7	0.942	3	11.3	1.088	3	5.8	1.229	3	2.0	390	42.1	9.89
P24534	Elongation factor 1-beta OS=Homo sapiens GN=EEF1B2 PE=1 SV=	199.78	26.22	5	0.709	26	8.5	0.721	26	10.2	1.015	26	6.2	225	24.7	4.67
Q9UNM6	26S proteasome non-ATPase regulatory subunit 13 OS=Homo sapi	198.66	11.97	2	0.730	15	62.7	0.860	14	23.3	1.259	14	40.4	376	42.9	5.81
Q14978	Nucleolar and coiled-body phosphoprotein 1 OS=Homo sapiens GN	195.82	12.30	8	1.156	21	17.0	1.034	21	21.8	0.864	21	24.5	699	73.6	9.47
O43175	D-3-phosphoglycerate dehydrogenase OS=Homo sapiens GN=PHG	194.84	23.45	9	0.733	25	12.2	0.848	25	11.6	1.221	25	10.2	533	56.6	6.71
Q9UL46	Proteasome activator complex subunit 2 OS=Homo sapiens GN=PS	192.94	30.13	6	0.681	12	9.7	0.922	12	16.4	1.327	12	9.1	239	27.4	5.73
P30084	Enoyl-CoA hydratase, mitochondrial OS=Homo sapiens GN=ECHS1	192.02	19.31	3	1.301	21	16.5	1.011	21	19.5	0.781	21	16.1	290	31.4	8.07
P17174	Aspartate aminotransferase, cytoplasmic OS=Homo sapiens GN=G	191.38	24.70	8	0.839	29	4.7	0.907	29	9.6	1.079	29	11.2	413	46.2	7.01
Q96KP4	Cytosolic non-specific dipeptidase OS=Homo sapiens GN=CNDP2 F	191.02	47.16	16	0.726	39	12.2	**0.602**	39	13.9	0.826	39	12.0	475	52.8	5.97
P25787	Proteasome subunit alpha type-2 OS=Homo sapiens GN=PSMA2 P	190.52	28.63	5	0.703	19	12.3	0.866	19	23.3	1.193	19	12.0	234	25.9	7.43
Q58FG0	Putative heat shock protein HSP 90-alpha A5 OS=Homo sapiens GI	189.28	17.07	6	0.869	1		1.105	1		1.272	1		334	38.7	6.57
Q7KZF4	Staphylococcal nuclease domain-containing protein 1 OS=Homo sa	188.02	9.56	7	0.800	19	18.5	0.717	19	17.4	0.913	19	28.5	910	101.9	7.17
O94808	Glutamine--fructose-6-phosphate aminotransferase [isomerizing] 2	187.67	5.28	2	**0.649**	11	15.1	0.875	11	8.6	1.330	11	7.4	682	76.9	7.37
O76061	Stanniocalcin-2 OS=Homo sapiens GN=STC2 PE=1 SV=1 - [STC2_	187.62	26.82	6	1.290	32	26.8	**2.053**	32	41.7	**1.601**	32	21.3	302	33.2	7.30
Q9UHL4	Dipeptidyl peptidase 2 OS=Homo sapiens GN=DPP7 PE=1 SV=3 -	185.84	12.20	4	0.948	11	8.6	1.108	11	11.0	1.169	11	17.5	492	54.3	6.32
P55795	Heterogeneous nuclear ribonucleoprotein H2 OS=Homo sapiens GI	184.54	28.29	11	0.903	1		0.980	1		1.086	1		449	49.2	6.30
P62906	60S ribosomal protein L10a OS=Homo sapiens GN=RPL10A PE=1	183.89	25.81	6	0.986	25	9.5	0.839	25	8.1	0.899	25	21.8	217	24.8	9.94
P55060	Exportin-2 OS=Homo sapiens GN=CSE1L PE=1 SV=3 - [XPO2_HU	181.21	12.98	7	0.709	53	12.2	**0.436**	53	59.1	**0.611**	53	49.5	971	110.3	5.77
Q9NTK5	Obg-like ATPase 1 OS=Homo sapiens GN=OLA1 PE=1 SV=2 - [OL	179.52	9.09	3	0.697	13	15.5	0.699	13	15.0	1.007	13	5.9	396	44.7	7.81
Q9H2U2	Inorganic pyrophosphatase 2, mitochondrial OS=Homo sapiens GN	176.00	24.55	8	1.080	14	7.8	0.982	14	15.5	0.900	14	6.8	334	37.9	7.39
P61088	Ubiquitin-conjugating enzyme E2 N OS=Homo sapiens GN=UBE2N	174.31	71.05	8	0.762	30	17.5	0.976	30	22.9	1.324	30	14.7	152	17.1	6.57
Q14103	Heterogeneous nuclear ribonucleoprotein D0 OS=Homo sapiens GI	172.40	30.14	12	0.901	19	8.1	1.140	19	14.2	1.262	19	5.6	355	38.4	7.81
Q15233	Non-POU domain-containing octamer-binding protein OS=Homo sa	171.34	16.99	7	0.881	18	14.3	0.817	18	25.4	0.895	18	28.8	471	54.2	8.95
P06753	Tropomyosin alpha-3 chain OS=Homo sapiens GN=TPM3 PE=1 SV	169.02	26.32	10	0.877	8	11.9	0.905	8	10.4	1.044	8	8.7	285	32.9	4.72
Q13838	Spliceosome RNA helicase DDX39B OS=Homo sapiens GN=DDX39	168.44	21.03	7	0.840	21	12.0	1.043	21	19.3	1.236	21	14.9	428	49.0	5.67
P35221	Catenin alpha-1 OS=Homo sapiens GN=CTNNA1 PE=1 SV=1 - [CT	168.36	16.78	11	0.876	16	7.6	0.843	16	6.6	0.977	16	4.3	906	100.0	6.29
P30153	Serine/threonine-protein phosphatase 2A 65 kDa regulatory subuni	166.33	16.13	6	0.698	17	21.9	**0.658**	17	19.5	1.043	17	14.9	589	65.3	5.11
P14550	Alcohol dehydrogenase [NADP(+)] OS=Homo sapiens GN=AKR1A	164.23	48.00	12	0.838	41	9.6	0.981	41	13.4	1.206	41	11.3	325	36.5	6.79
Q92688	Acidic leucine-rich nuclear phosphoprotein 32 family member B OS	163.10	33.07	7	0.835	19	13.5	0.964	19	16.1	1.157	19	20.8	251	28.8	4.06
Q9H254	Spectrin beta chain, non-erythrocytic 4 OS=Homo sapiens GN=SP	162.39	0.70	2	0.854	2	8.4	**1.841**	2	5.2	**2.155**	2	13.6	2564	288.8	6.01

Q3LXA3	Bifunctional ATP-dependent dihydroxyacetone kinase/FAD-AMP lya	162.26	5.22	2	0.643	6	10.3	0.580	6	20.4	1.002	6	12.0	575	58.9	7.49
P22234	Multifunctional protein ADE2 OS=Homo sapiens GN=PAICS PE=1 S	160.99	28.71	8	0.769	33	7.0	0.888	33	16.6	1.142	33	10.0	425	47.0	7.23
Q9BUD6	Spondin-2 OS=Homo sapiens GN=SPON2 PE=1 SV=3 - [SPON2_H	159.80	17.22	5	0.819	20	6.4	0.786	20	38.6	0.922	20	26.6	331	35.8	5.52
Q9H1E3	Nuclear ubiquitous casein and cyclin-dependent kinase substrate 1	159.06	18.52	4	0.605	23	21.8	0.760	23	13.4	1.197	23	15.3	243	27.3	5.08
P21266	Glutathione S-transferase Mu 3 OS=Homo sapiens GN=GSTM3 PE	158.69	17.78	3	0.743	10	7.8	0.913	10	16.0	1.199	10	11.5	225	26.5	5.54
P01833	Polymeric immunoglobulin receptor OS=Homo sapiens GN=PIGR F	155.62	3.53	2	0.899	8	16.4	0.976	8	30.3	1.131	8	6.5	764	83.2	5.74
Q9UHI8	A disintegrin and metalloproteinase with thrombospondin motifs 1	153.34	5.58	4	1.421	8	20.4	1.594	8	21.3	1.236	8	32.1	967	105.3	6.83
P15531	Nucleoside diphosphate kinase A OS=Homo sapiens GN=NME1 PE	153.14	57.89	9	0.897	20	12.2	1.010	20	31.4	1.111	20	20.9	152	17.1	6.19
P63208	S-phase kinase-associated protein 1 OS=Homo sapiens GN=SKP1 I	152.60	15.34	2	0.781	13	15.0	1.139	13	10.4	1.361	13	12.3	163	18.6	4.54
P27816	Microtubule-associated protein 4 OS=Homo sapiens GN=MAP4 PE	150.93	8.51	8	0.954	15	5.2	1.249	15	19.7	1.302	15	11.3	1152	120.9	5.43
O00629	Importin subunit alpha-3 OS=Homo sapiens GN=KPNA4 PE=1 SV=	149.26	3.26	1	0.778	8	5.4	0.716	8	5.1	0.891	8	5.6	521	57.9	4.96
P14174	Macrophage migration inhibitory factor OS=Homo sapiens GN=MIF	148.28	46.09	5	0.852	38	21.6	1.097	38	29.0	1.236	38	36.2	115	12.5	7.88
P23526	Adenosylhomocysteinase OS=Homo sapiens GN=AHCY PE=1 SV=4	148.28	26.39	10	0.784	29	8.4	0.582	29	20.5	0.729	29	14.0	432	47.7	6.34
P25789	Proteasome subunit alpha type-4 OS=Homo sapiens GN=PSMA4 P	147.26	41.76	7	0.704	18	11.5	0.809	18	18.3	1.179	18	12.2	261	29.5	7.72
Q04837	Single-stranded DNA-binding protein, mitochondrial OS=Homo sap	144.21	55.41	8	1.226	22	25.5	1.156	22	19.3	0.913	22	4.9	148	17.2	9.60
P30044	Peroxiredoxin-5, mitochondrial OS=Homo sapiens GN=PRDX5 PE=	144.13	40.65	6	0.744	27	20.0	0.947	27	35.8	1.208	27	17.9	214	22.1	8.70
Q13098	COP9 signalosome complex subunit 1 OS=Homo sapiens GN=GPS	143.52	7.94	3	0.967	9	26.9	0.910	9	60.9	1.056	9	92.8	491	55.5	6.74
Q9UQ80	Proliferation-associated protein 2G4 OS=Homo sapiens GN=PA2G4	142.65	32.99	9	0.748	23	11.1	0.866	23	8.9	1.173	23	6.7	394	43.8	6.55
P00167	Cytochrome b5 OS=Homo sapiens GN=CYB5A PE=1 SV=2 - [CYB5	142.43	33.58	4	1.142	18	26.7	1.490	18	21.4	1.297	18	8.5	134	15.3	4.96
P28074	Proteasome subunit beta type-5 OS=Homo sapiens GN=PSMB5 PE	140.80	26.24	6	0.669	17	11.6	0.833	17	11.1	1.243	17	14.2	263	28.5	6.92
P55786	Puromycin-sensitive aminopeptidase OS=Homo sapiens GN=NPEPI	139.73	11.10	6	0.762	12	5.7	0.870	12	34.6	1.010	12	16.7	919	103.2	5.72
Q9Y224	UPF0568 protein C14orf166 OS=Homo sapiens GN=C14orf166 PE	139.10	27.46	5	0.718	16	12.9	0.839	16	7.4	1.229	16	25.0	244	28.1	6.65
P68431	Histone H3.1 OS=Homo sapiens GN=HIST1H3A PE=1 SV=2 - [H3	139.00	54.41	8	0.696	19	18.4	0.185	6	66.3	0.292	6	78.6	136	15.4	11.12
P23246	Splicing factor, proline- and glutamine-rich OS=Homo sapiens GN=	138.71	12.87	7	0.981	25	11.8	1.045	25	16.0	1.087	26	21.6	707	76.1	9.44
Q08380	Galectin-3-binding protein OS=Homo sapiens GN=LGALS3BP PE=1	138.24	19.49	9	1.110	28	20.5	1.925	28	47.3	1.754	28	23.7	585	65.3	5.27
P06576	ATP synthase subunit beta, mitochondrial OS=Homo sapiens GN=A	137.09	17.01	5	0.926	15	18.1	0.834	15	31.3	0.991	15	39.4	529	56.5	5.40
P51858	Hepatoma-derived growth factor OS=Homo sapiens GN=HDGF PE	136.36	39.58	9	0.809	21	9.3	1.149	21	10.3	1.437	21	15.2	240	26.8	4.73
P08123	Collagen alpha-2(I) chain OS=Homo sapiens GN=COL1A2 PE=1 SV	136.30	2.71	4	0.912	19	18.8	0.788	19	29.9	0.970	19	21.7	1366	129.2	8.95
Q13765	Nascent polypeptide-associated complex subunit alpha OS=Homo	135.36	28.84	5	0.843	16	5.4	1.034	16	8.2	1.245	16	12.9	215	23.4	4.56
Q86V81	THO complex subunit 4 OS=Homo sapiens GN=ALYREF PE=1 SV=	134.19	27.63	6	0.864	28	17.4	1.257	28	17.1	1.394	28	17.8	257	26.9	11.15
P09012	U1 small nuclear ribonucleoprotein A OS=Homo sapiens GN=SNRF	134.14	21.28	5	0.790	13	10.8	1.207	13	20.5	1.350	13	6.8	282	31.3	9.83
P30043	Flavin reductase (NADPH) OS=Homo sapiens GN=BLVRB PE=1 SV	133.25	65.05	8	0.799	17	16.8	0.800	17	14.9	1.076	17	21.7	206	22.1	7.65
P14625	Endoplasmin OS=Homo sapiens GN=HSP90B1 PE=1 SV=1 - [ENPI	131.58	10.96	9	1.015	18	9.1	1.405	18	21.8	1.433	18	10.3	803	92.4	4.84
P16401	Histone H1.5 OS=Homo sapiens GN=HIST1H1B PE=1 SV=3 - [H1	131.41	24.34	8	0.404	22	51.7	0.956	22	13.0	2.332	22	32.6	226	22.6	10.92
Q13185	Chromobox protein homolog 3 OS=Homo sapiens GN=CBX3 PE=1	130.98	33.33	5	0.806	18	7.8	0.947	18	11.7	1.135	18	11.1	183	20.8	5.33
P07919	Cytochrome b-c1 complex subunit 6, mitochondrial OS=Homo sap	130.62	28.57	2	1.064	7	10.0	0.968	7	17.7	0.986	7	7.1	91	10.7	4.44
O75390	Citrate synthase, mitochondrial OS=Homo sapiens GN=CS PE=1 S	128.52	19.31	7	1.012	20	10.1	0.752	20	12.1	0.744	20	13.8	466	51.7	8.32
Q99598	Translin-associated protein X OS=Homo sapiens GN=TSNAX PE=1	128.49	6.55	1	0.855	5	2.7	0.948	5	21.4	1.089	5	20.4	290	33.1	6.55
P55327	Tumor protein D52 OS=Homo sapiens GN=TPD52 PE=1 SV=2 - [T	128.45	55.80	8	0.725	25	17.2	1.903	25	13.8	2.665	25	23.4	224	24.3	4.83
P21333	Filamin-A OS=Homo sapiens GN=FLNA PE=1 SV=4 - [FLNA_HUMA	127.33	5.18	10	0.805	8	8.9	0.742	8	13.7	0.893	8	13.3	2647	280.6	6.06
Q96C90	Protein phosphatase 1 regulatory subunit 14B OS=Homo sapiens G	127.30	8.84	1	0.876	7	20.7	1.018	7	5.6	1.185	7	15.2	147	15.9	4.86
Q15365	Poly(rC)-binding protein 1 OS=Homo sapiens GN=PCBP1 PE=1 SV	127.30	32.30	10	0.701	15	18.8	0.922	15	12.8	1.343	15	11.6	356	37.5	7.09
P41567	Eukaryotic translation initiation factor 1 OS=Homo sapiens GN=EIF	125.51	11.50	1	0.845	5	10.0	0.904	5	5.3	1.238	5	15.3	113	12.7	7.44
P30085	UMP-CMP kinase OS=Homo sapiens GN=CMPK1 PE=1 SV=3 - [KC	125.39	29.59	5	1.048	22	11.7	1.142	22	9.0	1.103	22	7.0	196	22.2	5.57
P28066	Proteasome subunit alpha type-5 OS=Homo sapiens GN=PSMA5 P	123.69	20.33	4	0.756	15	15.9	0.946	15	12.3	1.248	15	7.1	241	26.4	4.79
P25398	40S ribosomal protein S12 OS=Homo sapiens GN=RPS12 PE=1 SV	123.61	40.91	4	0.817	11	12.7	0.887	11	17.9	1.015	11	10.3	132	14.5	7.21
Q8NC51	Plasminogen activator inhibitor 1 RNA-binding protein OS=Homo s	123.21	21.08	8	0.861	15	8.7	1.159	15	11.6	1.302	15	12.0	408	44.9	8.65
P12081	Histidine--tRNA ligase, cytoplasmic OS=Homo sapiens GN=HARS F	123.05	17.29	6	0.865	15	8.1	0.994	15	9.8	1.158	15	8.9	509	57.4	5.88
P60981	Destrin OS=Homo sapiens GN=DSTN PE=1 SV=3 - [DEST_HUMAN	120.15	34.55	4	0.958	14	13.3	1.132	14	33.0	1.244	14	11.4	165	18.5	7.85
O43765	Small glutamine-rich tetratricopeptide repeat-containing protein al	119.85	7.67	2	0.868	5	8.8	1.035	5	11.9	1.153	5	36.5	313	34.0	4.87
Q8NCW5	NAD(P)H-hydrate epimerase OS=Homo sapiens GN=APOA1BP PE=	119.32	36.81	6	0.816	22	10.5	0.920	22	12.0	1.133	22	15.7	288	31.7	7.66
P18463	HLA class I histocompatibility antigen, B-37 alpha chain OS=Homo	119.31	20.17	6	1.064	11	22.1	2.495	11	33.7	1.988	11	26.8	362	40.4	6.15
P09960	Leukotriene A-4 hydrolase OS=Homo sapiens GN=LTA4H PE=1 SV	117.53	2.95	1	0.585	5	95.2	0.771	5	74.3	1.296	5	74.5	611	69.2	6.18
P26641	Elongation factor 1-gamma OS=Homo sapiens GN=EEF1G PE=1 S	117.34	30.43	8	0.714	19	14.7	0.616	19	39.9	0.843	19	30.7	437	50.1	6.67
P29692	Elongation factor 1-delta OS=Homo sapiens GN=EEF1D PE=1 SV=	117.04	31.67	6	0.846	23	11.8	0.818	23	9.4	1.007	23	10.7	281	31.1	5.01
O14818	Proteasome subunit alpha type-7 OS=Homo sapiens GN=PSMA7 P	116.98	35.48	8	0.778	19	6.2	0.908	19	15.0	1.190	19	12.5	248	27.9	8.46
Q8WUM4	Programmed cell death 6-interacting protein OS=Homo sapiens GN	116.48	2.76	2	0.860	7	11.0	0.685	7	10.7	0.752	7	12.5	868	96.0	6.52
Q15366	Poly(rC)-binding protein 2 OS=Homo sapiens GN=PCBP2 PE=1 SV	116.07	14.79	5	0.885	11	11.6	1.165	11	20.1	1.275	11	10.8	365	38.6	6.79
P61769	Beta-2-microglobulin OS=Homo sapiens GN=B2M PE=1 SV=1 - [B	115.94	65.55	8	1.167	52	26.7	2.868	52	64.9	2.523	52	27.2	119	13.7	6.52
P78371	T-complex protein 1 subunit beta OS=Homo sapiens GN=CCT2 PE	113.56	14.21	6	0.678	17	34.6	0.496	17	62.8	0.854	17	32.7	535	57.5	6.46
Q9Y2B0	Protein canopy homolog 2 OS=Homo sapiens GN=CNPY2 PE=1 SV	113.14	34.07	4	1.044	12	16.8	1.172	12	14.8	1.184	12	16.0	182	20.6	4.92
P14854	Cytochrome c oxidase subunit 6B1 OS=Homo sapiens GN=COX6B1	112.17	55.81	4	1.074	16	22.2	1.157	16	21.9	1.057	16	9.5	86	10.2	7.05
P67936	Tropomyosin alpha-4 chain OS=Homo sapiens GN=TPM4 PE=1 SV	111.39	25.40	8	0.896	7	11.5	1.018	7	9.5	1.143	7	11.7	248	28.5	4.69
Q16881	Thioredoxin reductase 1, cytoplasmic OS=Homo sapiens GN=TXNI	110.65	23.11	8	0.836	14	10.3	0.855	14	13.0	1.123	14	17.6	649	70.9	7.39
P54725	UV excision repair protein RAD23 homolog A OS=Homo sapiens G	110.64	8.54	4	0.930	6	19.1	1.726	6	51.3	2.048	6	66.2	363	39.6	4.58
O00303	Eukaryotic translation initiation factor 3 subunit F OS=Homo sapier	110.30	9.52	2	0.626	14	19.7	0.798	14	26.5	1.327	14	19.6	357	37.5	5.45
P43490	Nicotinamide phosphoribosyltransferase OS=Homo sapiens GN=N/	109.68	19.55	6	0.955	15	7.6	1.558	15	9.5	1.588	15	12.5	491	55.5	7.15
Q13501	Sequestosome-1 OS=Homo sapiens GN=SQSTM1 PE=1 SV=1 - [S	109.25	8.41	2	0.810	9	14.5	0.875	9	26.7	1.069	9	11.2	440	47.7	5.22
P09493	Tropomyosin alpha-1 chain OS=Homo sapiens GN=TPM1 PE=1 SV	106.60	18.31	6	1.048	3	6.3	1.069	3	6.3	1.171	3	1.4	284	32.7	4.74
Q99538	Legumain OS=Homo sapiens GN=LGMN PE=1 SV=1 - [LGMN_HUI	106.21	4.39	1	1.151	4	8.6	1.425	4	16.2	1.238	4	7.5	433	49.4	6.55
P62316	Small nuclear ribonucleoprotein Sm D2 OS=Homo sapiens GN=SN	105.61	38.98	5	0.903	18	7.4	0.954	18	10.2	1.073	18	10.5	118	13.5	9.91
P50502	Hsc70-interacting protein OS=Homo sapiens GN=ST13 PE=1 SV=2	105.06	26.56	10	0.822	33	10.4	0.882	33	18.4	1.072	33	9.7	369	41.3	5.27
Q92597	Protein NDRG1 OS=Homo sapiens GN=NDRG1 PE=1 SV=1 - [NDR	104.49	20.30	5	0.962	9	19.9	3.183	9	49.0	2.899	9	34.6	394	42.8	5.82
Q15084	Protein disulfide-isomerase A6 OS=Homo sapiens GN=PDIA6 PE=1	104.20	17.95	5	0.888	21	11.7	1.261	21	16.8	1.416	21	16.0	440	48.1	5.08
P13667	Protein disulfide-isomerase A4 OS=Homo sapiens GN=PDIA4 PE=1	103.70	12.87	6	1.157	10	14.4	1.241	10	50.4	1.145	10	24.7	645	72.9	5.07
Q6PKG0	La-related protein 1 OS=Homo sapiens GN=LARP1 PE=1 SV=2 - [I	103.18	3.65	4	0.881	8	12.9	1.039	8	21.2	1.130	8	12.0	1096	123.4	8.82
O00232	26S proteasome non-ATPase regulatory subunit 12 OS=Homo sapi	102.86	7.89	2	0.731	8	4.6	0.485	8	22.9	0.678	8	13.0	456	52.9	7.65
O75787	Renin receptor OS=Homo sapiens GN=ATP6AP2 PE=1 SV=2 - [RE	102.16	16.57	3	1.024	8	13.9	1.400	8	10.7	1.259	8	17.3	350	39.0	6.10
Q04760	Lactoylglutathione lyase OS=Homo sapiens GN=GLO1 PE=1 SV=4	101.63	42.39	7	1.016	22	13.6	1.032	22	17.8	1.035	22	14.7	184	20.8	5.31
P07108	Acyl-CoA-binding protein OS=Homo sapiens GN=DBI PE=1 SV=2 -	101.58	65.52	6	0.949	18	12.0	1.336	18	12.2	1.422	18	7.1	87	10.0	6.57
P30050	60S ribosomal protein L12 OS=Homo sapiens GN=RPL12 PE=1 SV	101.44	40.00	4	0.879	16	6.4	0.745	16	40.0	0.827	16	25.1	165	17.8	9.42
Q03154	Aminoacylase-1 OS=Homo sapiens GN=ACY1 PE=1 SV=1 - [ACY1_	101.05	12.50	4	0.961	10	19.0	1.223	10	38.5	1.087	10	37.1	408	45.9	6.18
Q96HY6	DDRGK domain-containing protein 1 OS=Homo sapiens GN=DDRG	100.82	5.41	1	1.039	6	9.0	0.977	6	8.1	0.972	6	11.1	314	35.6	5.12
Q14118	Dystroglycan OS=Homo sapiens GN=DAG1 PE=1 SV=2 - [DAG1_H	100.39	9.94	6	1.181	19	20.1	1.661	19	20.3	1.446	19	16.8	895	97.4	8.56
P20073	Annexin A7 OS=Homo sapiens GN=ANXA7 PE=1 SV=3 - [ANXA7_	100.23	7.38	4	0.981	7	29.1	0.984	7	7.4	1.062	7	12.8	488	52.7	5.68
P50990	T-complex protein 1 subunit theta OS=Homo sapiens GN=CCT8 PE	100.13	26.82	13	0.723	24	9.9	0.736	24	21.6	1.009	24	13.6	548	59.6	5.60
A6NDG6	Phosphoglycolate phosphatase OS=Homo sapiens GN=PGP PE=1 S	99.69	6.54	1	0.808	4	11.3	1.275	4	10.8	1.654	4	7.7	321	34.0	6.14
P06132	Uroporphyrinogen decarboxylase OS=Homo sapiens GN=UROD PE	97.93	5.99	1	0.813	4	3.0	0.782	4	2.8	0.949	4	3.9	367	40.8	6.14
P12109	Collagen alpha-1(VI) chain OS=Homo sapiens GN=COL6A1 PE=1 S	97.83	3.89	3	1.160	14	15.9	2.097	14	31.3	1.801	14	18.2	1028	108.5	5.43
P16949	Stathmin OS=Homo sapiens GN=STMN1 PE=1 SV=3 - [STMN1_HU	97.40	28.86	4	0.980	12	18.6	1.161	12	32.8	1.417	12	29.9	149	17.3	5.97
O60256	Phosphoribosyl pyrophosphate synthase-associated protein 2 OS=I	95.09	6.23	1	0.803	5	15.7	0.407	5	6.1	0.471	5	11.0	369	40.9	7.44
P61970	Nuclear transport factor 2 OS=Homo sapiens GN=NUTF2 PE=1 SV	94.46	74.80	6	0.883	14	10.9	1.087	14	5.6	1.204	14	12.5	127	14.5	5.38
P28070	Proteasome subunit beta type-4 OS=Homo sapiens GN=PSMB4 PE	93.93	31.06	6	0.742	16	9.7	0.837	16	17.5	1.082	16	9.5	264	29.2	5.97
O95831	Apoptosis-inducing factor 1, mitochondrial OS=Homo sapiens GN=	93.65	24.14	12	0.906	32	13.0	0.877	33	13.0	0.995	32	13.1	613	66.9	8.95
Q07666	KH domain-containing, RNA-binding, signal transduction-associate	93.15	7.67	4	0.855	8	17.3	0.854	8	15.0	1.114	8	31.3	443	48.2	8.66
Q92804	TATA-binding protein-associated factor 2N OS=Homo sapiens GN=	92.25	19.43	7	1.039	17	11.9	1.532	17	14.4	1.431	17	21.6	592	61.8	8.02
P11940	Polyadenylate-binding protein 1 OS=Homo sapiens GN=PABPC1 PI	92.15	19.97	10	0.873	14	8.7	1.066	14	14.8	1.249	14	5.7	636	70.6	9.50
Q15121	Astrocytic phosphoprotein PEA-15 OS=Homo sapiens GN=PEA15 F	91.10	30.00	3	0.891	8	7.2	0.980	8	5.5	1.116	8	7.2	130	15.0	5.02
P09622	Dihydrolipoyl dehydrogenase, mitochondrial OS=Homo sapiens GN	90.02	15.72	7	1.172	19	32.3	1.383	19	27.9	1.182	19	9.7	509	54.1	7.85
Q15631	Translin OS=Homo sapiens GN=TSN PE=1 SV=1 - [TSN_HUMAN]	89.84	17.98	3	0.773	10	18.2	0.978	10	14.9	1.210	10	6.6	228	26.2	6.44
P38646	Stress-70 protein, mitochondrial OS=Homo sapiens GN=HSPA9 PE	89.59	7.22	5	1.024	5	13.5	1.066	5	2.8	0.976	5	5.4	679	73.6	6.16
Q9UII2	ATPase inhibitor, mitochondrial OS=Homo sapiens GN=ATPIF1 PE	89.57	23.58	4	1.065	14	9.7	1.510	14	23.8	1.410	14	12.6	106	12.2	9.35
Q9BRA2	Thioredoxin domain-containing protein 17 OS=Homo sapiens GN=	89.56	38.21	4	0.989	18	11.4	0.981	18	8.0	1.021	18	11.9	123	13.9	5.52

P41250	Glycine--tRNA ligase OS=Homo sapiens GN=GARS PE=1 SV=3 - [S	88.46	20.57	12	0.796	20	10.5	0.700	20	31.2	0.911	20	25.8	739	83.1	7.03
O75223	Gamma-glutamylcyclotransferase OS=Homo sapiens GN=GGCT PE	88.18	32.98	7	1.088	20	5.8	1.154	20	6.2	1.050	20	7.0	188	21.0	5.14
P50991	T-complex protein 1 subunit delta OS=Homo sapiens GN=CCT4 PE	87.99	20.22	8	**0.615**	12	27.4	**0.546**	11	45.1	1.051	11	10.6	539	57.9	7.83
Q99471	Prefoldin subunit 5 OS=Homo sapiens GN=PFDN5 PE=1 SV=2 - [F	87.30	11.69	1	0.739	6	3.6	0.835	6	3.3	1.118	6	3.3	154	17.3	6.33
P35222	Catenin beta-1 OS=Homo sapiens GN=CTNNB1 PE=1 SV=1 - [CTN	87.13	18.44	10	0.769	19	13.1	0.903	19	26.7	1.014	19	27.1	781	85.4	5.86
Q9BWD1	Acetyl-CoA acetyltransferase, cytosolic OS=Homo sapiens GN=ACA	85.81	6.80	3	**0.426**	4	63.7	**0.321**	4	98.2	0.902	4	33.9	397	41.3	6.92
Q16531	DNA damage-binding protein 1 OS=Homo sapiens GN=DDB1 PE=1	85.79	3.16	4	0.795	9	9.9	0.781	9	8.6	1.035	9	5.6	1140	126.9	5.26
Q9UPQ0	LIM and calponin homology domains-containing protein 1 OS=Hon	85.57	2.68	3	0.906	3	7.1	0.996	3	7.0	1.117	3	9.4	1083	121.8	6.47
Q07021	Complement component 1 Q subcomponent-binding protein, mitoc	82.99	46.10	6	1.111	28	6.9	0.776	28	13.1	0.689	28	20.4	282	31.3	4.84
P47756	F-actin-capping protein subunit beta OS=Homo sapiens GN=CAPZI	82.78	14.08	2	0.970	13	3.5	1.167	13	10.9	1.222	13	10.9	277	31.3	5.59
Q86VP6	Cullin-associated NEDD8-dissociated protein 1 OS=Homo sapiens (	81.33	9.35	7	0.699	15	18.6	0.687	15	27.8	0.983	15	11.7	1230	136.3	5.78
Q9Y5L4	Mitochondrial import inner membrane translocase subunit Tim13 C	80.29	26.32	2	0.827	6	10.4	0.871	6	13.4	1.100	6	9.6	95	10.5	8.18
P48637	Glutathione synthetase OS=Homo sapiens GN=GSS PE=1 SV=1 - [	80.10	16.67	9	0.706	25	13.6	0.902	25	26.5	1.231	25	21.5	474	52.4	5.92
Q12874	Splicing factor 3A subunit 3 OS=Homo sapiens GN=SF3A3 PE=1 S	79.87	9.58	4	1.011	14	14.6	0.907	14	11.4	0.953	14	6.6	501	58.8	5.38
P53999	Activated RNA polymerase II transcriptional coactivator p15 OS=H	79.47	26.77	5	0.911	14	12.2	1.228	14	13.9	1.339	14	4.7	127	14.4	9.60
P68036	Ubiquitin-conjugating enzyme E2 L3 OS=Homo sapiens GN=UBE2L	78.91	24.03	2	0.784	13	8.8	1.019	13	10.7	1.217	13	11.7	154	17.9	8.51
Q9Y2V2	Calcium-regulated heat stable protein 1 OS=Homo sapiens GN=CA	78.82	10.88	1	0.804	5	1.8	1.016	5	14.0	1.279	5	7.0	147	15.9	8.21
P54727	UV excision repair protein RAD23 homolog B OS=Homo sapiens GI	78.29	32.03	12	0.828	18	9.6	0.988	18	10.0	1.202	18	12.1	409	43.1	4.84
Q01105	Protein SET OS=Homo sapiens GN=SET PE=1 SV=3 - [SET_HUMA	78.27	35.86	7	0.766	25	13.0	0.817	25	18.0	1.073	25	15.3	290	33.5	4.32
P09496	Clathrin light chain A OS=Homo sapiens GN=CLTA PE=1 SV=1 - [(	77.87	2.42	1	0.936	4	4.7	1.319	4	12.1	1.454	4	2.7	248	27.1	4.51
P25788	Proteasome subunit alpha type-3 OS=Homo sapiens GN=PSMA3 P	77.71	26.27	7	0.855	16	17.8	1.077	16	17.3	1.273	16	7.8	255	28.4	5.33
P07741	Adenine phosphoribosyltransferase OS=Homo sapiens GN=APRT P	77.70	11.11	1	0.857	5	11.0	0.839	5	6.0	0.989	5	12.0	180	19.6	6.02
P49368	T-complex protein 1 subunit gamma OS=Homo sapiens GN=CCT3	76.36	7.34	4	0.676	11	13.9	0.738	11	25.2	1.112	11	18.2	545	60.5	6.49
P62942	Peptidyl-prolyl cis-trans isomerase FKBP1A OS=Homo sapiens GN=	76.22	29.63	3	**1.631**	14	27.0	**2.767**	14	51.8	**1.610**	14	24.7	108	11.9	8.16
P61158	Actin-related protein 3 OS=Homo sapiens GN=ACTR3 PE=1 SV=3	75.94	20.57	5	1.032	15	8.4	0.872	15	18.1	0.864	15	14.1	418	47.3	5.88
P36543	V-type proton ATPase subunit E 1 OS=Homo sapiens GN=ATP6V1I	74.86	8.85	1	0.946	3	4.7	1.293	3	25.2	1.417	3	27.5	226	26.1	8.00
Q9Y696	Chloride intracellular channel protein 4 OS=Homo sapiens GN=CLI	74.82	9.49	2	0.968	6	12.9	1.147	6	2.3	1.215	6	10.5	253	28.8	5.59
Q14011	Cold-inducible RNA-binding protein OS=Homo sapiens GN=CIRBP	74.69	36.63	5	0.838	11	17.3	1.194	11	19.5	1.404	11	11.4	172	18.6	9.51
P30405	Peptidyl-prolyl cis-trans isomerase F, mitochondrial OS=Homo sapi	74.26	24.15	7	1.181	11	30.6	1.099	11	18.1	0.942	11	12.9	207	22.0	9.38
Q7L2H7	Eukaryotic translation initiation factor 3 subunit M OS=Homo sapie	72.97	17.38	3	0.684	11	13.1	1.011	11	18.2	1.393	11	14.1	374	42.5	5.63
P20700	Lamin-B1 OS=Homo sapiens GN=LMNB1 PE=1 SV=2 - [LMNB1_HI	72.70	33.11	18	0.933	44	13.3	1.164	44	19.6	1.228	44	11.9	586	66.4	5.16
Q92499	ATP-dependent RNA helicase DDX1 OS=Homo sapiens GN=DDX1	72.08	7.97	4	**0.657**	6	45.1	0.862	6	62.8	1.079	6	12.6	740	82.4	7.23
P09429	High mobility group protein B1 OS=Homo sapiens GN=HMGB1 PE=	71.42	33.02	7	**0.649**	15	13.4	1.185	15	8.0	**1.797**	15	20.1	215	24.9	5.74
P53396	ATP-citrate synthase OS=Homo sapiens GN=ACLY PE=1 SV=3 - [A	71.06	10.90	9	0.936	17	11.5	0.943	17	17.3	1.000	17	6.9	1101	120.8	7.33
O60888	Protein CutA OS=Homo sapiens GN=CUTA PE=1 SV=2 - [CUTA_H	70.75	40.78	4	0.922	18	12.1	0.997	18	17.0	1.113	18	9.6	179	19.1	5.50
Q16630	Cleavage and polyadenylation specificity factor subunit 6 OS=Hom	70.34	6.17	2	0.817	5	3.7	1.001	5	1.8	1.219	5	10.1	551	59.2	7.15
Q96TC7	Regulator of microtubule dynamics protein 3 OS=Homo sapiens GI	69.78	4.89	1	0.956	3	7.0	0.925	3	3.4	0.923	3	0.0	470	52.1	5.10
Q9UHK6	Alpha-methylacyl-CoA racemase OS=Homo sapiens GN=AMACR PE	69.77	7.59	3	1.008	4	4.0	1.118	4	5.7	1.118	4	2.9	382	42.4	6.44
Q96QK1	Vacuolar protein sorting-associated protein 35 OS=Homo sapiens (	69.33	8.04	5	0.845	9	11.3	0.696	9	61.3	0.774	9	44.5	796	91.6	5.49
Q9Y266	Nuclear migration protein nudC OS=Homo sapiens GN=NUDC PE=	68.61	17.22	6	0.989	17	7.5	1.081	17	7.8	1.129	17	22.8	331	38.2	5.38
P16930	Fumarylacetoacetase OS=Homo sapiens GN=FAH PE=1 SV=2 - [F/	68.33	10.26	4	0.977	7	5.2	0.986	7	1.6	0.925	7	14.2	419	46.3	6.95
P14324	Farnesyl pyrophosphate synthase OS=Homo sapiens GN=FDPS PE	68.31	20.53	6	0.997	4	9.5	1.034	4	3.9	1.083	4	4.5	419	48.2	6.15
P62314	Small nuclear ribonucleoprotein Sm D1 OS=Homo sapiens GN=SN	68.16	16.81	1	0.863	13	5.6	0.920	13	17.1	1.077	13	10.6	119	13.3	11.56
P68402	Platelet-activating factor acetylhydrolase IB subunit beta OS=Hom	67.77	16.59	3	**0.597**	8	14.3	**0.626**	8	31.2	1.019	8	9.9	229	25.6	5.92
Q9Y5S9	RNA-binding protein 8A OS=Homo sapiens GN=RBM8A PE=1 SV=	67.57	16.67	4	0.955	18	20.9	1.166	18	24.9	1.218	18	14.2	174	19.9	5.72
P02452	Collagen alpha-1(I) chain OS=Homo sapiens GN=COL1A1 PE=1 S\	67.39	3.83	6	0.801	18	15.0	0.706	18	21.3	0.840	18	9.1	1464	138.9	5.80
P50851	Lipopolysaccharide-responsive and beige-like anchor protein OS=H	66.63	1.71	3	1.045	6	52.3	0.776	6	28.4	0.731	6	70.8	2863	318.9	5.60
P06454	Prothymosin alpha OS=Homo sapiens GN=PTMA PE=1 SV=2 - [PT	66.61	27.93	3	0.942	8	14.3	**2.139**	8	10.7	**2.169**	8	7.9	111	12.2	3.78
P00491	Purine nucleoside phosphorylase OS=Homo sapiens GN=PNP PE=1	66.57	32.53	8	0.739	20	10.5	0.839	20	15.0	1.112	20	15.8	289	32.1	6.95
O15144	Actin-related protein 2/3 complex subunit 2 OS=Homo sapiens GN	66.56	17.00	5	0.838	11	6.9	1.011	11	25.1	1.185	11	23.0	300	34.3	7.36
Q96CN7	Isochorismatase domain-containing protein 1 OS=Homo sapiens G	66.46	6.38	1	0.958	3	3.7	1.073	3	13.2	1.148	3	5.7	298	32.2	7.39
Q99729	Heterogeneous nuclear ribonucleoprotein A/B OS=Homo sapiens (	64.76	16.57	7	0.944	11	11.9	1.204	11	10.1	1.268	11	8.5	332	36.2	8.21
P23381	Tryptophan--tRNA ligase, cytoplasmic OS=Homo sapiens GN=WAF	64.72	6.37	3	0.871	8	14.0	0.930	8	14.9	1.176	8	7.0	471	53.1	6.23
P13473	Lysosome-associated membrane glycoprotein 2 OS=Homo sapiens	64.71	5.12	2	0.876	6	14.7	0.742	6	18.1	0.873	6	5.2	410	44.9	5.63
Q15185	Prostaglandin E synthase 3 OS=Homo sapiens GN=PTGES3 PE=1 !	64.68	18.13	2	0.689	8	17.4	**0.610**	8	33.4	0.920	8	11.9	160	18.7	4.54
Q9BT78	COP9 signalosome complex subunit 4 OS=Homo sapiens GN=COP	64.62	4.93	1	0.776	4	2.3	0.880	4	3.0	1.131	4	3.0	406	46.2	5.83
P52272	Heterogeneous nuclear ribonucleoprotein M OS=Homo sapiens GN	64.30	4.79	3	1.022	8	5.7	0.924	8	7.6	0.934	8	4.7	730	77.5	8.70
P14866	Heterogeneous nuclear ribonucleoprotein L OS=Homo sapiens GN=	64.06	11.71	5	0.741	11	5.0	0.730	11	9.3	0.990	11	5.4	589	64.1	8.22
P14923	Junction plakoglobin OS=Homo sapiens GN=JUP PE=1 SV=3 - [PL	64.00	2.68	1	0.813	4	7.9	0.894	4	8.9	1.078	4	9.6	745	81.7	6.14
O00244	Copper transport protein ATOX1 OS=Homo sapiens GN=ATOX1 PE	63.91	36.76	2	0.845	6	14.7	0.844	6	23.4	1.055	6	2.0	68	7.4	7.24
Q9Y3B8	Oligoribonuclease, mitochondrial OS=Homo sapiens GN=REXO2 P	63.47	13.92	3	0.779	11	27.5	0.834	11	50.6	1.147	11	37.4	237	26.8	6.87
Q5VW32	BRO1 domain-containing protein BROX OS=Homo sapiens GN=BR	63.41	16.06	6	0.819	12	6.2	0.909	12	4.8	1.126	12	6.3	411	46.4	7.65
Q13813	Spectrin alpha chain, non-erythrocytic 1 OS=Homo sapiens GN=SF	63.34	1.62	2	0.733	3	14.2	0.859	3	1.5	1.172	3	18.2	2472	284.4	5.35
Q15393	Splicing factor 3B subunit 3 OS=Homo sapiens GN=SF3B3 PE=1 S	62.37	0.99	2	0.929	4	1.1	**0.615**	4	16.8	**0.667**	4	17.9	1217	135.5	5.26
Q92598	Heat shock protein 105 kDa OS=Homo sapiens GN=HSPH1 PE=1 !	62.25	5.13	3	0.945	4	4.2	1.148	4	11.2	1.216	4	6.4	858	96.8	5.39
Q14980	Nuclear mitotic apparatus protein 1 OS=Homo sapiens GN=NUMA1	62.24	2.13	5	0.821	3	3.4	1.029	3	18.7	1.253	3	22.2	2115	238.1	5.78
P16188	HLA class I histocompatibility antigen, A-30 alpha chain OS=Homo	62.10	13.15	4	0.991	2	11.5	**1.559**	2	6.4	**1.573**	2	5.0	365	40.9	6.10
P49721	Proteasome subunit beta type-2 OS=Homo sapiens GN=PSMB2 PE	61.71	36.32	4	0.799	7	8.5	0.933	7	14.6	1.219	7	8.3	201	22.8	7.02
O15067	Phosphoribosylformylglycinamidine synthase OS=Homo sapiens GI	61.69	1.42	1	**0.565**	2	66.2	1.077	2	57.8	**1.905**	2	163.3	1338	144.6	5.76
Q13740	CD166 antigen OS=Homo sapiens GN=ALCAM PE=1 SV=2 - [CD16	61.26	8.06	3	0.979	5	2.6	**1.526**	5	3.8	**1.570**	5	10.6	583	65.1	6.25
P26885	Peptidyl-prolyl cis-trans isomerase FKBP2 OS=Homo sapiens GN=F	61.04	10.56	1	1.055	7	12.7	**1.575**	7	19.7	1.493	7	17.4	142	15.6	9.13
Q02818	Nucleobindin-1 OS=Homo sapiens GN=NUCB1 PE=1 SV=4 - [NUC	60.90	16.70	6	0.987	12	16.6	1.113	12	27.0	1.190	12	23.8	461	53.8	5.25
Q15102	Platelet-activating factor acetylhydrolase IB subunit gamma OS=H	60.76	24.68	5	0.767	12	15.9	1.179	12	18.6	1.415	12	13.9	231	25.7	6.84
Q9UMX0	Ubiquilin-1 OS=Homo sapiens GN=UBQLN1 PE=1 SV=2 - [UBQL1_	60.15	2.21	1	0.964	3	17.7	1.085	3	21.8	1.145	3	2.5	589	62.5	5.11
P05023	Sodium/potassium-transporting ATPase subunit alpha-1 OS=Homo	59.99	0.78	1	0.929	2	5.8	0.684	2	12.3	0.736	2	6.5	1023	112.8	5.49
P02768	Serum albumin OS=Homo sapiens GN=ALB PE=1 SV=2 - [ALBU_H	59.90	4.43	2	1.344	6	5.0	0.890	6	7.3	**0.657**	6	6.2	609	69.3	6.28
P49902	Cytosolic purine 5'-nucleotidase OS=Homo sapiens GN=NT5C2 PE	59.79	3.39	1	0.962	5	6.8	1.263	5	9.3	1.288	5	6.5	561	64.9	6.14
Q9BPU6	Dihydropyrimidinase-related protein 5 OS=Homo sapiens GN=DPY	59.00	1.24	1	1.045	1		**0.132**	1		**0.127**	1		564	61.4	7.20
P07099	Epoxide hydrolase 1 OS=Homo sapiens GN=EPHX1 PE=1 SV=1 - [	58.61	12.75	4	1.100	7	16.3	1.116	7	15.8	0.924	7	30.6	455	52.9	7.25
P26583	High mobility group protein B2 OS=Homo sapiens GN=HMGB2 PE=	58.50	14.83	3	0.889	1		1.269	1		1.427	1		209	24.0	7.81
Q86UP2	Kinectin OS=Homo sapiens GN=KTN1 PE=1 SV=1 - [KTN1_HUMA	57.96	7.66	7	0.789	11	8.2	0.901	11	13.6	1.064	11	1.2	1357	156.2	5.64
Q15459	Splicing factor 3A subunit 1 OS=Homo sapiens GN=SF3A1 PE=1 S	57.60	6.43	3	1.002	4	4.3	1.071	4	5.6	1.173	4	12.0	793	88.8	5.22
P67809	Nuclease-sensitive element-binding protein 1 OS=Homo sapiens GI	57.57	23.46	5	0.906	13	7.1	1.143	13	23.9	1.206	13	18.8	324	35.9	9.88
O43776	Asparagine--tRNA ligase, cytoplasmic OS=Homo sapiens GN=NARS	57.51	5.84	3	0.719	6	15.6	0.897	6	10.7	1.179	6	15.3	548	62.9	6.25
Q6UXD5	Seizure 6-like protein 2 OS=Homo sapiens GN=SEZ6L2 PE=1 SV=2	57.19	4.07	2	1.196	5	15.8	**1.553**	5	27.2	1.153	5	29.2	910	97.5	4.89
O95861	3'(2'),5'-bisphosphate nucleotidase 1 OS=Homo sapiens GN=BPNT	56.77	16.88	4	0.812	6	12.8	0.843	6	13.8	1.059	6	19.9	308	33.4	5.69
Q9HC38	Glyoxalase domain-containing protein 4 OS=Homo sapiens GN=GL	56.70	8.31	3	0.825	7	17.3	1.261	7	5.1	1.500	7	12.0	313	34.8	5.60
Q9UBW8	COP9 signalosome complex subunit 7a OS=Homo sapiens GN=CO	56.43	5.82	1	0.836	3	22.7	1.027	3	6.2	1.125	3	14.3	275	30.3	8.22
P35237	Serpin B6 OS=Homo sapiens GN=SERPINB6 PE=1 SV=3 - [SPB6_	55.96	12.77	3	0.900	8	12.9	0.921	8	22.8	1.026	8	13.0	376	42.6	5.27
P30046	D-dopachrome decarboxylase OS=Homo sapiens GN=DDT PE=1 S	55.75	44.07	5	0.925	13	12.0	1.152	12	37.8	1.143	12	23.1	118	12.7	7.30
Q9H4A4	Aminopeptidase B OS=Homo sapiens GN=RNPEP PE=1 SV=2 - [AI	55.38	22.00	10	0.839	14	7.7	0.741	14	26.1	0.949	14	27.9	650	72.5	5.74
Q16836	Hydroxyacyl-coenzyme A dehydrogenase, mitochondrial OS=Homo	55.31	16.56	4	1.045	12	8.2	0.974	12	18.0	0.897	12	14.2	314	34.3	8.85
P42167	Lamina-associated polypeptide 2, isoforms beta/gamma OS=Homo	55.19	14.10	4	0.786	8	7.0	0.967	8	8.7	1.200	8	13.3	454	50.6	9.38
Q13404	Ubiquitin-conjugating enzyme E2 variant 1 OS=Homo sapiens GN=	55.17	10.88	2	0.753	2	12.3	1.019	2	4.6	1.354	2	7.7	147	16.5	7.93
Q15746	Myosin light chain kinase, smooth muscle OS=Homo sapiens GN=I	55.14	0.57	1	**5.587**	3	12.8	**7.926**	3	38.9	1.464	3	4.6	1914	210.6	6.15
Q5H9R7	Serine/threonine-protein phosphatase 6 regulatory subunit 3 OS=H	55.08	2.18	1	0.741	3	0.2	1.132	3	28.3	**1.527**	3	5.0	873	97.6	4.60
P05114	Non-histone chromosomal protein HMG-14 OS=Homo sapiens GN=	54.99	10.00	1	1.092	5	25.1	1.244	5	12.0	1.233	5	13.9	100	10.7	9.60
P55209	Nucleosome assembly protein 1-like 1 OS=Homo sapiens GN=NAP	54.94	14.32	4	0.719	8	18.2	0.694	8	8.8	1.041	8	12.7	391	45.3	4.46
P15311	Ezrin OS=Homo sapiens GN=EZR PE=1 SV=4 - [EZRI_HUMAN]	54.59	16.72	8	0.853	10	9.1	0.797	10	13.8	0.941	10	24.7	586	69.4	6.27
Q13443	Disintegrin and metalloproteinase domain-containing protein 9 OS=	54.53	1.95	1	0.810	3	2.9	**1.849**	3	24.4	**2.403**	3	32.8	819	90.5	7.52
P21964	Catechol O-methyltransferase OS=Homo sapiens GN=COMT PE=1	54.38	5.54	1	0.805	3	1.5	0.769	3	3.3	0.966	3	12.4	271	30.0	5.47
P25311	Zinc-alpha-2-glycoprotein OS=Homo sapiens GN=AZGP1 PE=1 SV=	54.10	16.78	4	0.980	13	5.5	**2.670**	13	34.8	**2.953**	13	31.7	298	34.2	6.05
P25786	Proteasome subunit alpha type-1 OS=Homo sapiens GN=PSMA1 P	53.96	43.73	10	0.810	15	19.1	0.914	15	24.7	1.169	15	11.5	263	29.5	6.61

Q9H173	Nucleotide exchange factor SIL1 OS=Homo sapiens GN=SIL1 PE=	53.77	5.64	2	1.133	2	20.3	1.306	2	21.2	1.153	2	0.9	461	52.1	5.36
Q99436	Proteasome subunit beta type-7 OS=Homo sapiens GN=PSMB7 PE	53.29	20.58	3	0.859	12	18.1	1.092	12	11.2	1.253	12	18.6	277	29.9	7.68
Q13263	Transcription intermediary factor 1-beta OS=Homo sapiens GN=TF	53.23	6.95	6	**0.598**	13	10.5	**0.578**	13	14.1	1.012	13	17.8	835	88.5	5.77
Q15819	Ubiquitin-conjugating enzyme E2 variant 2 OS=Homo sapiens GN=	53.19	13.10	2	0.979	3	0.3	1.294	3	4.0	1.234	3	1.2	145	16.4	8.09
Q13435	Splicing factor 3B subunit 2 OS=Homo sapiens GN=SF3B2 PE=1 S	52.98	2.12	3	0.896	7	11.2	0.753	7	19.5	0.840	7	11.8	895	100.2	5.67
P08133	Annexin A6 OS=Homo sapiens GN=ANXA6 PE=1 SV=3 - [ANXA6_	52.68	6.24	4	0.944	7	7.5	1.154	7	11.1	1.310	7	15.0	673	75.8	5.60
P05026	Sodium/potassium-transporting ATPase subunit beta-1 OS=Homo	52.60	9.90	3	0.830	7	6.1	0.954	7	27.9	1.153	7	20.1	303	35.0	8.53
P12004	Proliferating cell nuclear antigen OS=Homo sapiens GN=PCNA PE=	52.26	21.07	3	0.844	5	1.4	0.867	5	10.3	1.045	5	9.2	261	28.8	4.69
P17987	T-complex protein 1 subunit alpha OS=Homo sapiens GN=TCP1 PI	51.83	7.91	3	**0.575**	4	10.0	**0.442**	4	15.9	0.782	4	8.6	556	60.3	6.11
Q01130	Serine/arginine-rich splicing factor 2 OS=Homo sapiens GN=SRSF2	51.64	4.98	1	0.916	11	26.2	1.477	11	5.8	**1.657**	11	21.1	221	25.5	11.85
Q99613	Eukaryotic translation initiation factor 3 subunit C OS=Homo sapie	51.55	3.18	3	0.703	4	5.6	0.727	4	6.8	1.059	4	2.8	913	105.3	5.68
P13693	Translationally-controlled tumor protein OS=Homo sapiens GN=TP	51.54	27.33	4	0.908	10	9.9	0.958	10	8.8	1.097	10	7.9	172	19.6	4.93
P62158	Calmodulin OS=Homo sapiens GN=CALM1 PE=1 SV=2 - [CALM_H	51.40	59.06	6	0.763	12	16.9	1.177	12	20.8	1.362	12	20.0	149	16.8	4.22
Q7Z6Z7	E3 ubiquitin-protein ligase HUWE1 OS=Homo sapiens GN=HUWE1	50.58	0.64	2	0.772	4	1.6	0.865	4	1.0	1.135	4	2.6	4374	481.6	5.22
P49458	Signal recognition particle 9 kDa protein OS=Homo sapiens GN=SI	50.44	32.56	3	0.834	8	16.3	**1.617**	8	11.4	**1.805**	8	18.0	86	10.1	7.97
O75531	Barrier-to-autointegration factor OS=Homo sapiens GN=BANF1 PE	49.86	13.48	1	0.782	5	7.7	1.236	5	4.8	**1.741**	5	5.4	89	10.1	6.09
Q9HB71	Calcyclin-binding protein OS=Homo sapiens GN=CACYBP PE=1 SV	49.79	29.39	6	0.804	9	9.0	0.919	9	12.7	1.142	9	9.2	228	26.2	8.25
Q9UNZ2	NSFL1 cofactor p47 OS=Homo sapiens GN=NSFL1C PE=1 SV=2 -	49.65	18.38	7	1.056	11	19.2	1.400	11	24.6	1.343	11	9.7	370	40.5	5.10
P43243	Matrin-3 OS=Homo sapiens GN=MATR3 PE=1 SV=2 - [MATR3_HU	49.20	3.78	2	0.745	5	2.7	0.742	5	19.2	1.044	5	10.3	847	94.6	6.25
P52803	Ephrin-A5 OS=Homo sapiens GN=EFNA5 PE=1 SV=1 - [EFNA5_HU	49.04	3.07	1	0.936	4	6.4	1.215	4	14.1	1.275	4	4.6	228	26.3	6.42
O43390	Heterogeneous nuclear ribonucleoprotein R OS=Homo sapiens GN	48.83	12.80	8	0.858	3	11.9	0.991	3	17.1	1.035	3	16.5	633	70.9	8.13
P62318	Small nuclear ribonucleoprotein Sm D3 OS=Homo sapiens GN=SN	48.14	23.81	2	0.899	5	12.6	1.004	5	6.0	1.026	5	20.2	126	13.9	10.32
P17900	Ganglioside GM2 activator OS=Homo sapiens GN=GM2A PE=1 SV=	48.03	31.09	4	1.154	7	11.4	**1.909**	7	24.3	**1.591**	7	12.3	193	20.8	5.31
Q15257	Serine/threonine-protein phosphatase 2A activator OS=Homo sapi	47.86	15.64	3	0.820	5	7.1	1.103	5	6.9	1.327	5	10.0	358	40.6	5.94
P13798	Acylamino-acid-releasing enzyme OS=Homo sapiens GN=APEH PE	47.80	9.43	6	0.729	13	16.4	0.864	13	39.0	1.098	13	19.1	732	81.2	5.48
O75643	U5 small nuclear ribonucleoprotein 200 kDa helicase OS=Homo sa	47.45	2.25	4	0.907	7	6.7	0.817	7	18.9	0.834	7	25.6	2136	244.4	6.06
Q96FJ2	Dynein light chain 2, cytoplasmic OS=Homo sapiens GN=DYNLL2 I	47.36	20.22	2	0.823	7	4.1	0.969	7	13.5	1.143	7	5.4	89	10.3	7.37
P61956	Small ubiquitin-related modifier 2 OS=Homo sapiens GN=SUMO2 I	47.12	18.95	2	**0.661**	7	12.2	1.033	7	4.7	1.498	7	14.4	95	10.9	5.50
Q96P70	Importin-9 OS=Homo sapiens GN=IPO9 PE=1 SV=3 - [IPO9_HUM	46.46	5.67	2	**0.598**	2	39.6	0.760	2	56.9	1.271	2	14.8	1041	115.9	4.81
Q96M27	Protein PRRC1 OS=Homo sapiens GN=PRRC1 PE=1 SV=1 - [PRRC	46.46	3.15	1	**0.652**	7	12.5	**0.644**	7	25.0	1.066	7	20.4	445	46.7	5.83
P61326	Protein mago nashi homolog OS=Homo sapiens GN=MAGOH PE=1	46.30	14.38	2	0.896	7	16.0	1.203	7	20.0	1.233	7	8.8	146	17.2	6.11
O00461	Golgi integral membrane protein 4 OS=Homo sapiens GN=GOLIM4	45.98	4.60	3	1.416	6	4.7	**2.017**	6	23.7	**1.551**	6	11.3	696	81.8	4.77
P12830	Cadherin-1 OS=Homo sapiens GN=CDH1 PE=1 SV=3 - [CADH1_H	45.92	5.67	4	**1.656**	15	17.2	**2.266**	15	12.7	1.469	15	10.6	882	97.4	4.73
Q13283	Ras GTPase-activating protein-binding protein 1 OS=Homo sapiens	45.55	4.94	2	0.904	2	4.3	0.993	2	9.3	1.098	2	13.6	466	52.1	5.52
P60900	Proteasome subunit alpha type-6 OS=Homo sapiens GN=PSMA6 P	45.46	35.37	8	0.744	13	7.4	0.864	13	6.1	1.158	13	9.2	246	27.4	6.76
O14745	Na(+)/H(+) exchange regulatory cofactor NHE-RF1 OS=Homo sap	45.41	18.99	7	1.129	14	12.6	**1.730**	14	28.8	1.380	14	21.4	358	38.8	5.77
P16035	Metalloproteinase inhibitor 2 OS=Homo sapiens GN=TIMP2 PE=1	45.35	27.27	5	**1.622**	8	23.0	**2.040**	8	27.9	1.361	8	14.7	220	24.4	7.49
O95881	Thioredoxin domain-containing protein 12 OS=Homo sapiens GN=	45.29	19.19	3	0.890	5	29.8	1.312	5	8.2	1.382	5	16.0	172	19.2	5.40
Q9ULC4	Malignant T-cell-amplified sequence 1 OS=Homo sapiens GN=MCT	45.22	9.39	1	**0.546**	2	9.4	0.739	2	1.7	1.354	2	11.1	181	20.5	8.82
P13489	Ribonuclease inhibitor OS=Homo sapiens GN=RNH1 PE=1 SV=2 -	45.18	8.03	2	0.725	3	2.9	0.918	3	7.6	1.228	3	14.6	461	49.9	4.82
P26368	Splicing factor U2AF 65 kDa subunit OS=Homo sapiens GN=U2AF2	45.16	10.74	3	0.782	4	8.1	0.863	4	21.1	1.028	4	2.6	475	53.5	9.09
P34913	Bifunctional epoxide hydrolase 2 OS=Homo sapiens GN=EPHX2 PE	45.09	3.78	1	0.898	3	3.1	1.026	3	8.9	1.098	3	3.2	555	62.6	6.28
P14678	Small nuclear ribonucleoprotein-associated proteins B and B' OS=H	44.95	9.17	3	0.897	7	11.1	0.943	7	8.7	1.052	7	9.1	240	24.6	11.19
Q9Y265	RuvB-like 1 OS=Homo sapiens GN=RUVBL1 PE=1 SV=1 - [RUVB1_	44.93	11.62	4	0.840	4	14.4	0.814	4	9.1	0.994	4	1.3	456	50.2	6.42
Q96CT7	Coiled-coil domain-containing protein 124 OS=Homo sapiens GN=	44.87	9.87	1	0.798	3	1.0	0.847	3	6.6	1.068	3	7.6	223	25.8	9.54
O00479	High mobility group nucleosome-binding domain-containing protei	44.71	10.00	1	1.187	4	1.1	**1.760**	4	9.5	**1.511**	4	5.6	90	9.5	10.48
Q6P587	Acylpyruvase FAHD1, mitochondrial OS=Homo sapiens GN=FAHD1	44.53	3.13	1	0.960	3	4.9	1.030	3	6.2	1.031	3	10.6	224	24.8	7.39
Q9NYF8	Bcl-2-associated transcription factor 1 OS=Homo sapiens GN=BCL	44.43	5.76	6	0.969	7	0.8	0.889	7	14.6	0.914	7	17.5	920	106.1	9.98
Q96DG6	Carboxymethylenebutenolidase homolog OS=Homo sapiens GN=C	44.09	6.94	1	0.681	3	7.3	1.249	3	6.7	**1.537**	3	1.7	245	28.0	7.18
P35241	Radixin OS=Homo sapiens GN=RDX PE=1 SV=1 - [RADI_HUMAN]	43.95	9.78	5	0.776	3	0.1	0.934	3	5.5	1.204	3	8.2	583	68.5	6.37
P23588	Eukaryotic translation initiation factor 4B OS=Homo sapiens GN=E	43.85	9.17	3	0.918	4	23.3	1.315	4	16.0	1.249	4	28.1	611	69.1	5.73
Q8NAB2	Kelch repeat and BTB domain-containing protein 3 OS=Homo sapi	43.40	1.15	1	0.743	13	11.0	0.786	13	14.2	1.063	13	10.4	608	69.3	5.39
P58107	Epiplakin OS=Homo sapiens GN=EPPK1 PE=1 SV=2 - [EPIPL_HUM	43.29	3.14	2	0.695	1		1.079	1		**1.552**	1		5090	555.3	5.60
Q10567	AP-1 complex subunit beta-1 OS=Homo sapiens GN=AP1B1 PE=1	42.54	7.06	4	0.770	5	6.1	0.901	5	9.8	1.095	5	12.8	949	104.6	5.06
P30042	ES1 protein homolog, mitochondrial OS=Homo sapiens GN=C21or	42.39	15.67	3	1.929	10	26.3	**1.911**	10	11.9	1.003	10	11.3	268	28.2	8.27
Q07955	Serine/arginine-rich splicing factor 1 OS=Homo sapiens GN=SRSF1	42.30	28.63	6	1.111	17	13.2	1.433	17	18.6	1.262	17	10.5	248	27.7	10.36
Q13247	Serine/arginine-rich splicing factor 6 OS=Homo sapiens GN=SRSF6	41.95	5.52	2	1.008	7	5.7	1.194	7	19.6	1.214	7	6.2	344	39.6	11.43
Q9H3U1	Protein unc-45 homolog A OS=Homo sapiens GN=UNC45A PE=1 S	41.63	2.12	1	0.809	2	7.2	1.001	2	1.7	1.236	2	8.9	944	103.0	6.07
Q99832	T-complex protein 1 subunit eta OS=Homo sapiens GN=CCT7 PE=	41.53	7.55	4	**0.595**	10	19.3	**0.378**	10	29.0	0.764	10	32.7	543	59.3	7.65
P02144	Myoglobin OS=Homo sapiens GN=MB PE=1 SV=2 - [MYG_HUMAN	41.08	31.82	5	0.836	8	9.7	1.062	8	14.5	1.238	8	10.9	154	17.2	7.68
O00625	Pirin OS=Homo sapiens GN=PIR PE=1 SV=1 - [PIR_HUMAN]	40.95	4.14	1	0.721	4	7.4	0.696	4	6.6	0.919	4	4.9	290	32.1	6.92
P00568	Adenylate kinase isoenzyme 1 OS=Homo sapiens GN=AK1 PE=1 S	40.85	25.26	3	0.768	10	24.0	0.969	10	10.8	1.261	10	19.5	194	21.6	8.63
Q15427	Splicing factor 3B subunit 4 OS=Homo sapiens GN=SF3B4 PE=1 S	40.71	14.15	3	0.834	8	3.4	0.928	8	14.4	1.089	8	16.4	424	44.4	8.56
Q96HE7	ERO1-like protein alpha OS=Homo sapiens GN=ERO1L PE=1 SV=2	40.25	2.35	1	1.153	3	1.1	**4.025**	3	16.3	**3.620**	3	23.0	468	54.4	5.68
P30049	ATP synthase subunit delta, mitochondrial OS=Homo sapiens GN=	40.21	8.33	1	0.999	4	2.2	1.225	4	6.2	1.200	4	9.5	168	17.5	5.49
P22307	Non-specific lipid-transfer protein OS=Homo sapiens GN=SCP2 PE	39.57	11.33	5	1.298	10	9.4	1.186	10	8.4	0.905	10	1.9	547	59.0	6.89
P09237	Matrilysin OS=Homo sapiens GN=MMP7 PE=1 SV=1 - [MMP7_HUM	39.56	2.62	1	0.808	1		**2.408**	1		**2.980**	1		267	29.7	7.91
P32929	Cystathionine gamma-lyase OS=Homo sapiens GN=CTH PE=1 SV=	39.51	4.94	1	0.978	3	6.2	1.204	3	0.8	1.162	3	5.8	405	44.5	6.70
Q15149	Plectin OS=Homo sapiens GN=PLEC PE=1 SV=3 - [PLEC_HUMAN]	39.38	0.96	3	1.078	2	0.4	1.117	2	33.6	1.036	2	34.1	4684	531.5	5.96
Q08209	Serine/threonine-protein phosphatase 2B catalytic subunit alpha is	39.35	3.45	2	0.883	2	10.7	1.265	2	5.6	1.433	2	5.1	521	58.7	5.86
Q8N3U4	Cohesin subunit SA-2 OS=Homo sapiens GN=STAG2 PE=1 SV=3 -	39.16	1.22	1	0.853	2	4.6	0.817	2	1.3	0.959	2	5.8	1231	141.2	5.43
Q14157	Ubiquitin-associated protein 2-like OS=Homo sapiens GN=UBAP2L	39.10	0.83	1	0.837	2	2.6	0.972	2	0.8	1.161	2	3.5	1087	114.5	7.11
P35637	RNA-binding protein FUS OS=Homo sapiens GN=FUS PE=1 SV=1	38.91	4.75	2	0.732	3	4.6	1.026	3	5.0	1.446	3	0.5	526	53.4	9.36
Q12907	Vesicular integral-membrane protein VIP36 OS=Homo sapiens GN=	38.73	5.90	1	0.847	3	2.3	1.012	3	3.3	1.195	3	5.6	356	40.2	6.95
P02795	Metallothionein-2 OS=Homo sapiens GN=MT2A PE=1 SV=1 - [MT2	38.49	19.67	1	0.789	4	4.8	**5.227**	4	1.0	**6.623**	4	5.6	61	6.0	7.83
Q15435	Protein phosphatase 1 regulatory subunit 7 OS=Homo sapiens GN=	38.44	12.78	3	0.768	4	3.1	0.746	4	4.8	0.968	4	4.3	360	41.5	4.91
O15372	Eukaryotic translation initiation factor 3 subunit H OS=Homo sapie	38.41	14.20	3	0.927	4	24.6	1.027	4	2.5	1.152	4	22.0	352	39.9	6.54
Q92928	Putative Ras-related protein Rab-1C OS=Homo sapiens GN=RAB1C	38.35	18.91	3	0.824	4	2.8	0.868	4	3.0	1.053	4	5.4	201	22.0	5.43
Q00839	Heterogeneous nuclear ribonucleoprotein U OS=Homo sapiens GN	38.26	7.52	5	0.896	11	14.5	0.943	11	19.6	1.053	11	25.6	825	90.5	6.00
P49189	4-trimethylaminobutyraldehyde dehydrogenase OS=Homo sapiens	38.12	14.78	7	0.897	11	10.2	0.705	11	16.3	0.777	11	8.1	494	53.8	5.87
P15559	NAD(P)H dehydrogenase [quinone] 1 OS=Homo sapiens GN=NQO	37.96	18.61	5	0.966	9	7.9	**0.519**	9	48.4	**0.570**	9	47.5	274	30.8	8.88
O15347	High mobility group protein B3 OS=Homo sapiens GN=HMGB3 PE=	37.64	6.00	1	0.880	1		0.966	1		1.098	1		200	23.0	8.37
Q9Y490	Talin-1 OS=Homo sapiens GN=TLN1 PE=1 SV=3 - [TLN1_HUMAN	37.55	2.01	4	0.823	5	14.4	0.790	5	18.5	0.999	5	5.3	2541	269.6	6.07
O00148	ATP-dependent RNA helicase DDX39A OS=Homo sapiens GN=DDX	37.55	9.84	4	0.850	1		1.008	1		1.187	1		427	49.1	5.68
P51991	Heterogeneous nuclear ribonucleoprotein A3 OS=Homo sapiens GI	37.31	8.99	5	0.956	6	6.3	1.139	6	10.3	1.194	6	8.5	378	39.6	9.01
P11216	Glycogen phosphorylase, brain form OS=Homo sapiens GN=PYGB	37.27	4.51	2	0.993	2	21.4	1.048	2	26.2	1.055	2	4.6	843	96.6	6.86
Q13310	Polyadenylate-binding protein 4 OS=Homo sapiens GN=PABPC4 PI	37.12	12.73	5	1.051	1		1.131	1		1.076	1		644	70.7	9.26
O43242	26S proteasome non-ATPase regulatory subunit 3 OS=Homo sapie	36.99	4.87	2	0.871	4	14.3	1.241	4	35.9	1.229	4	35.9	534	60.9	8.44
P39019	40S ribosomal protein S19 OS=Homo sapiens GN=RPS19 PE=1 SV	36.85	11.72	2	0.729	1		1.308	1		**1.793**	1		145	16.1	10.32
Q96FW1	Ubiquitin thioesterase OTUB1 OS=Homo sapiens GN=OTUB1 PE=1	36.52	12.18	3	0.782	7	13.7	0.874	7	4.1	1.204	7	19.6	271	31.3	4.94
O00584	Ribonuclease T2 OS=Homo sapiens GN=RNASET2 PE=1 SV=2 - [I	36.50	18.75	4	**1.911**	6	11.8	**2.479**	6	9.1	1.306	6	3.7	256	29.5	7.08
P60891	Ribose-phosphate pyrophosphokinase 1 OS=Homo sapiens GN=PF	36.47	7.86	2	0.828	3	1.0	0.826	3	1.4	0.878	3	15.3	318	34.8	6.98
P45974	Ubiquitin carboxyl-terminal hydrolase 5 OS=Homo sapiens GN=US	36.39	4.08	3	0.789	5	3.6	0.848	5	27.9	1.076	5	18.0	858	95.7	5.03
P12270	Nucleoprotein TPR OS=Homo sapiens GN=TPR PE=1 SV=3 - [TPR	36.32	3.05	6	1.041	7	14.1	0.937	7	23.3	0.935	7	10.5	2363	267.1	5.02
Q9NYL9	Tropomodulin-3 OS=Homo sapiens GN=TMOD3 PE=1 SV=1 - [TM	36.30	6.25	1	0.797	1		1.022	1		1.281	1		352	39.6	5.19
P51659	Peroxisomal multifunctional enzyme type 2 OS=Homo sapiens GN=	36.18	1.49	1	0.809	2	2.0	0.964	2	14.2	1.192	2	16.2	736	79.6	8.84
P46087	Putative ribosomal RNA methyltransferase NOP2 OS=Homo sapien	35.94	0.99	1	0.944	2	14.0	1.382	2	19.8	1.464	2	5.7	812	89.2	9.23
P62491	Ras-related protein Rab-11A OS=Homo sapiens GN=RAB11A PE=1	35.91	11.11	2	0.772	4	4.7	0.994	4	6.6	1.272	4	3.0	216	24.4	6.57
O14737	Programmed cell death protein 5 OS=Homo sapiens GN=PDCD5 P	35.83	10.40	1	0.882	3	0.7	1.131	3	15.5	1.304	3	2.4	125	14.3	6.04
Q14019	Coactosin-like protein OS=Homo sapiens GN=COTL1 PE=1 SV=3 -	35.80	11.27	1	0.875	3	4.8	1.345	3	11.8	**1.609**	3	8.7	142	15.9	5.67
Q9UK41	Vacuolar protein sorting-associated protein 28 homolog OS=Homo	35.47	6.79	1	0.871	3	5.5	1.199	3	11.4	1.212	3	15.4	221	25.4	5.54
Q9H9Q2	COP9 signalosome complex subunit 7b OS=Homo sapiens GN=CO	35.46	6.82	1	0.727	3	9.1	1.178	3	4.9	**1.729**	3	4.8	264	29.6	6.15
P55957	BH3-interacting domain death agonist OS=Homo sapiens GN=BID	35.45	7.69	1	0.743	1		1.156	1		**1.556**	1		195	22.0	5.44

Q8WZA0	Protein LZIC OS=Homo sapiens GN=LZIC PE=1 SV=1 - [LZIC_HUM	35.27	14.74	2	0.928	2	3.3	1.345	2	6.9	1.450	2	3.6	190	21.5	4.94
Q9UFN0	Protein NipSnap homolog 3A OS=Homo sapiens GN=NIPSNAP3A F	34.58	15.79	4	1.026	7	16.0	0.845	7	18.4	0.899	7	34.6	247	28.4	9.16
Q969H8	UPF0556 protein C19orf10 OS=Homo sapiens GN=C19orf10 PE=1	34.23	17.92	4	1.085	15	13.4	1.362	15	15.5	1.271	15	6.6	173	18.8	6.68
Q9BQT9	Calsyntenin-3 OS=Homo sapiens GN=CLSTN3 PE=1 SV=1 - [CSTN	33.68	1.15	2	1.096	5	24.4	1.870	5	41.0	1.629	5	11.1	956	106.0	5.44
P35579	Myosin-9 OS=Homo sapiens GN=MYH9 PE=1 SV=4 - [MYH9_HUM	33.37	3.88	6	0.838	4	4.6	0.878	4	6.5	1.080	4	5.8	1960	226.4	5.60
Q92616	Translational activator GCN1 OS=Homo sapiens GN=GCN1L1 PE=1	33.35	1.54	2	0.722	4	14.7	0.603	4	13.4	0.842	4	11.6	2671	292.6	7.47
P26038	Moesin OS=Homo sapiens GN=MSN PE=1 SV=3 - [MOES_HUMAN	33.35	8.15	5	1.006	1		1.091	1		1.084	1		577	67.8	6.40
P38606	V-type proton ATPase catalytic subunit A OS=Homo sapiens GN=A	33.23	2.92	1	0.741	1		0.663	1		0.894	1		617	68.3	5.52
O75347	Tubulin-specific chaperone A OS=Homo sapiens GN=TBCA PE=1 S	33.14	16.67	2	1.263	4	13.9	1.984	4	16.0	1.488	4	13.8	108	12.8	5.29
O43598	2'-deoxynucleoside 5'-phosphate N-hydrolase 1 OS=Homo sapiens	33.10	43.68	4	0.738	6	10.2	0.855	6	22.9	1.201	6	12.3	174	19.1	5.05
Q96AE4	Far upstream element-binding protein 1 OS=Homo sapiens GN=FU	33.02	13.51	10	0.877	9	2.8	1.037	9	6.9	1.187	9	1.0	644	67.5	7.61
O75884	Putative hydrolase RBBP9 OS=Homo sapiens GN=RBBP9 PE=1 SV	33.00	3.23	1	1.012	3	7.1	1.542	3	6.5	1.483	3	11.1	186	21.0	6.20
Q92945	Far upstream element-binding protein 2 OS=Homo sapiens GN=KH	33.00	11.53	6	0.894	5	3.4	1.244	5	9.8	1.392	5	6.4	711	73.1	7.30
O60664	Perilipin-3 OS=Homo sapiens GN=PLIN3 PE=1 SV=3 - [PLIN3_HU	32.95	4.38	1	0.946	1		1.155	1		1.221	1		434	47.0	5.44
Q96DB5	Regulator of microtubule dynamics protein 1 OS=Homo sapiens GI	32.92	2.23	1	0.863	2	2.4	1.251	2	8.1	1.450	2	5.6	314	35.8	8.50
O75533	Splicing factor 3B subunit 1 OS=Homo sapiens GN=SF3B1 PE=1 S	32.90	6.06	4	0.912	5	13.8	0.890	5	10.5	0.961	5	12.6	1304	145.7	7.09
P07686	Beta-hexosaminidase subunit beta OS=Homo sapiens GN=HEXB PI	32.75	6.65	3	1.230	6	7.6	1.395	6	2.7	1.140	6	4.7	556	63.1	6.76
O14561	Acyl carrier protein, mitochondrial OS=Homo sapiens GN=NDUFAB	32.52	5.77	1	1.118	4	2.9	0.970	4	3.3	0.855	4	3.2	156	17.4	4.93
P35244	Replication protein A 14 kDa subunit OS=Homo sapiens GN=RPA3	32.52	14.05	1	0.875	2	6.8	1.159	2	11.5	1.324	2	4.7	121	13.6	5.08
P43487	Ran-specific GTPase-activating protein OS=Homo sapiens GN=RAN	32.15	28.86	5	0.735	6	9.3	0.968	6	13.5	1.289	6	4.7	201	23.3	5.29
P61916	Epididymal secretory protein E1 OS=Homo sapiens GN=NPC2 PE=	32.07	38.41	4	0.922	11	5.2	1.797	11	15.8	1.821	11	8.5	151	16.6	7.65
Q9UKK9	ADP-sugar pyrophosphatase OS=Homo sapiens GN=NUDT5 PE=1	31.94	10.96	2	0.894	5	9.8	0.945	5	17.7	1.135	5	13.0	219	24.3	4.94
O43747	AP-1 complex subunit gamma-1 OS=Homo sapiens GN=AP1G1 PE	31.90	2.92	2	0.956	2	9.7	0.867	2	3.2	0.907	2	6.5	822	91.3	6.80
O75475	PC4 and SFRS1-interacting protein OS=Homo sapiens GN=PSIP1 F	31.73	4.15	3	0.954	1		1.119	1		1.173	1		530	60.1	9.13
P10155	60 kDa SS-A/Ro ribonucleoprotein OS=Homo sapiens GN=TROVE2	31.70	4.65	2	0.840	3	4.3	0.787	3	9.0	1.003	3	3.5	538	60.6	8.03
Q15843	NEDD8 OS=Homo sapiens GN=NEDD8 PE=1 SV=1 - [NEDD8_HUM	31.26	24.69	2	0.671	6	8.5	1.025	6	12.2	1.591	6	7.6	81	9.1	8.43
P17096	High mobility group protein HMG-I/HMG-Y OS=Homo sapiens GN=	31.16	15.89	2	0.983	5	1.2	1.155	5	5.8	1.171	5	14.9	107	11.7	10.32
Q9HAT2	Sialate O-acetylesterase OS=Homo sapiens GN=SIAE PE=1 SV=1 -	31.10	2.87	1	1.040	2	4.0	1.311	2	6.3	1.260	2	2.3	523	58.3	7.33
P54578	Ubiquitin carboxyl-terminal hydrolase 14 OS=Homo sapiens GN=U	31.09	4.05	2	0.861	2	6.5	0.943	2	28.8	1.095	2	22.1	494	56.0	5.30
P62304	Small nuclear ribonucleoprotein E OS=Homo sapiens GN=SNRPE F	31.01	52.17	3	0.880	8	6.0	0.917	8	4.9	1.026	8	7.0	92	10.8	9.44
P05204	Non-histone chromosomal protein HMG-17 OS=Homo sapiens GN=	30.94	22.22	2	1.384	5	4.1	2.678	5	48.1	1.891	5	24.9	90	9.4	9.99
P62857	40S ribosomal protein S28 OS=Homo sapiens GN=RPS28 PE=1 SV	30.94	15.94	1	0.911	4	5.3	1.151	4	2.9	1.210	4	6.2	69	7.8	10.70
P29218	Inositol monophosphatase 1 OS=Homo sapiens GN=IMPA1 PE=1 S	30.92	13.72	3	0.850	3	17.3	0.871	3	22.6	1.155	3	10.4	277	30.2	5.26
P46976	Glycogenin-1 OS=Homo sapiens GN=GYG1 PE=1 SV=4 - [GLYG_H	30.92	2.00	1	0.935	2	0.7	1.018	2	12.5	1.089	2	13.3	350	39.4	5.53
Q16629	Serine/arginine-rich splicing factor 7 OS=Homo sapiens GN=SRSF7	30.90	3.78	1	0.955	2	3.8	0.931	2	4.2	0.975	2	0.4	238	27.4	11.82
P40855	Peroxisomal biogenesis factor 19 OS=Homo sapiens GN=PEX19 PE	30.85	5.02	1	0.979	3	5.8	0.989	3	1.5	1.162	3	0.5	299	32.8	4.34
Q15370	Transcription elongation factor B polypeptide 2 OS=Homo sapiens	30.39	19.49	3	0.878	9	13.3	1.055	9	16.2	1.133	9	9.8	118	13.1	4.88
Q9UHV9	Prefoldin subunit 2 OS=Homo sapiens GN=PFDN2 PE=1 SV=1 - [F	30.36	7.79	1	0.985	3	7.8	1.047	3	13.3	1.009	3	5.5	154	16.6	6.58
Q9GZS3	WD repeat-containing protein 61 OS=Homo sapiens GN=WDR61 F	30.26	6.56	1	0.772	2	3.3	0.924	2	16.2	1.196	2	19.5	305	33.6	5.47
Q96EP5	DAZ-associated protein 1 OS=Homo sapiens GN=DAZAP1 PE=1 SV	30.22	15.48	4	0.833	6	12.9	0.966	6	9.2	1.204	6	8.0	407	43.4	8.56
Q3MJ13	WD repeat-containing protein 72 OS=Homo sapiens GN=WDR72 F	30.15	0.54	1	0.775	2	5.7	0.814	2	5.1	1.050	2	10.8	1102	123.3	6.67
P49006	MARCKS-related protein OS=Homo sapiens GN=MARCKSL1 PE=1 S	30.11	18.46	3	0.816	8	7.9	1.064	8	5.2	1.318	8	6.2	195	19.5	4.67
P61201	COP9 signalosome complex subunit 2 OS=Homo sapiens GN=COP	30.10	7.90	2	0.921	3	3.7	0.773	3	1.9	0.910	3	5.1	443	51.6	5.53
Q96BJ3	Axin interactor, dorsalization-associated protein OS=Homo sapiens	30.05	14.38	3	0.955	6	10.2	0.981	6	30.8	1.074	6	10.8	306	35.0	6.55
Q96TA1	Niban-like protein 1 OS=Homo sapiens GN=FAM129B PE=1 SV=3	30.00	4.69	2	0.812	3	9.6	0.991	3	14.9	1.190	3	11.1	746	84.1	6.19
P62495	Eukaryotic peptide chain release factor subunit 1 OS=Homo sapier	29.80	2.97	1	0.769	1		0.722	1		0.939	1		437	49.0	5.71
P25705	ATP synthase subunit alpha, mitochondrial OS=Homo sapiens GN=	29.66	6.51	2	0.677	2	27.7	0.728	2	3.7	1.074	2	31.6	553	59.7	9.13
P08473	Neprilysin OS=Homo sapiens GN=MME PE=1 SV=2 - [NEP_HUMAI	29.66	7.20	5	1.258	6	10.9	1.076	6	26.6	0.816	6	20.7	750	85.5	5.73
P20810	Calpastatin OS=Homo sapiens GN=CAST PE=1 SV=4 - [ICAL_HUM	29.63	3.53	3	0.720	6	4.0	1.379	6	7.5	1.959	6	6.2	708	76.5	5.07
P02794	Ferritin heavy chain OS=Homo sapiens GN=FTH1 PE=1 SV=2 - [FI	29.51	30.60	5	0.862	9	15.3	1.050	9	16.0	1.171	9	7.2	183	21.2	5.55
P10586	Receptor-type tyrosine-protein phosphatase F OS=Homo sapiens G	29.48	4.98	8	1.117	7	28.7	1.466	7	38.0	1.313	7	10.2	1907	212.7	6.30
P78527	DNA-dependent protein kinase catalytic subunit OS=Homo sapiens	29.22	0.46	1	0.885	2	16.2	0.573	2	11.6	0.647	2	4.6	4128	468.8	7.12
Q96HR9	Receptor expression-enhancing protein 6 OS=Homo sapiens GN=F	29.12	7.07	1	1.258	5	38.0	1.158	5	5.6	0.928	5	5.3	184	20.7	8.56
P61289	Proteasome activator complex subunit 3 OS=Homo sapiens GN=PS	29.00	8.66	2	0.793	5	7.2	0.842	5	5.2	1.048	5	6.3	254	29.5	5.95
Q92530	Proteasome inhibitor PI31 subunit OS=Homo sapiens GN=PSMF1 F	28.90	8.49	2	0.935	4	9.3	1.917	4	12.8	2.184	4	14.3	271	29.8	5.74
Q15428	Splicing factor 3A subunit 2 OS=Homo sapiens GN=SF3A2 PE=1 S	28.83	1.51	1	0.897	3	14.1	0.892	3	7.9	0.995	3	0.9	464	49.2	9.64
O14908	PDZ domain-containing protein GIPC1 OS=Homo sapiens GN=GIP(	28.78	6.61	1	1.154	2	9.6	1.186	2	9.4	1.028	2	0.2	333	36.0	6.28
Q9Y4L1	Hypoxia up-regulated protein 1 OS=Homo sapiens GN=HYOU1 PE	28.65	2.40	2	1.117	2	4.6	0.639	2	0.7	0.572	2	5.4	999	111.3	5.22
P61758	Prefoldin subunit 3 OS=Homo sapiens GN=VBP1 PE=1 SV=3 - [PF	28.43	9.64	1	0.866	2	5.1	1.112	2	0.3	1.284	2	5.4	197	22.6	7.11
P61960	Ubiquitin-fold modifier 1 OS=Homo sapiens GN=UFM1 PE=1 SV=1	28.36	17.65	1	0.878	3	11.4	1.078	3	7.7	1.228	3	2.8	85	9.1	9.31
Q13151	Heterogeneous nuclear ribonucleoprotein A0 OS=Homo sapiens GI	28.26	9.84	4	1.306	2	16.9	1.827	2	24.8	1.399	2	7.6	305	30.8	9.29
P08397	Porphobilinogen deaminase OS=Homo sapiens GN=HMBS PE=1 S\	28.24	5.54	1	0.636	2	7.6	0.759	2	10.0	1.192	2	2.4	361	39.3	7.18
P13797	Plastin-3 OS=Homo sapiens GN=PLS3 PE=1 SV=4 - [PLST_HUMAN	27.93	9.68	4	0.859	6	4.5	0.829	6	11.3	0.941	6	3.5	630	70.8	5.60
Q6IBS0	Twinfilin-2 OS=Homo sapiens GN=TWF2 PE=1 SV=2 - [TWF2_HU	27.60	2.01	1	0.896	2	2.0	1.035	2	0.0	1.155	2	2.0	349	39.5	6.84
P04156	Major prion protein OS=Homo sapiens GN=PRNP PE=1 SV=1 - [PI	27.46	3.56	1	0.853	1		2.768	1		3.245	1		253	27.6	9.00
P46940	Ras GTPase-activating-like protein IQGAP1 OS=Homo sapiens GN=	27.45	2.35	2	1.325	5	57.3	1.201	5	32.7	0.969	5	12.4	1657	189.1	6.48
P07305	Histone H1.0 OS=Homo sapiens GN=H1F0 PE=1 SV=3 - [H10_HU	27.40	11.86	2	0.601	5	26.8	1.107	5	7.6	1.752	5	38.0	194	20.9	10.84
Q99985	Semaphorin-3C OS=Homo sapiens GN=SEMA3C PE=1 SV=2 - [SEI	27.37	4.26	4	1.214	7	20.5	5.272	7	24.6	4.238	7	13.8	751	85.2	8.69
Q9NP62	Chorion-specific transcription factor GCMa OS=Homo sapiens GN=	27.33	1.38	1	0.723	2	2.2	0.815	2	4.9	1.127	2	7.2	436	49.2	7.02
Q8NBS9	Thioredoxin domain-containing protein 5 OS=Homo sapiens GN=T	27.32	8.33	2	1.152	3	13.8	1.373	3	5.7	1.143	3	16.7	432	47.6	5.97
Q14914	Prostaglandin reductase 1 OS=Homo sapiens GN=PTGR1 PE=1 SV	27.27	8.81	2	1.272	3	1.8	1.062	3	2.6	0.839	3	11.8	329	35.8	8.29
A8MWD9	Putative small nuclear ribonucleoprotein G-like protein 15 OS=Hon	27.08	35.53	3	0.928	9	16.7	0.962	9	24.2	1.025	9	9.5	76	8.5	8.84
P55809	Succinyl-CoA:3-ketoacid coenzyme A transferase 1, mitochondrial (	26.99	8.85	2	0.775	2	16.3	0.883	2	11.1	1.140	2	5.1	520	56.1	7.46
P52907	F-actin-capping protein subunit alpha-1 OS=Homo sapiens GN=CA	26.93	16.78	4	0.957	8	13.9	1.044	8	22.6	1.123	8	10.3	286	32.9	5.69
P35613	Basigin OS=Homo sapiens GN=BSG PE=1 SV=2 - [BASI_HUMAN]	26.77	3.64	1	1.286	3	9.8	2.089	3	3.9	1.561	3	5.9	385	42.2	5.66
Q9BRK5	45 kDa calcium-binding protein OS=Homo sapiens GN=SDF4 PE=1	26.61	22.38	6	1.190	8	13.6	1.559	8	29.9	1.321	8	20.3	362	41.8	4.86
Q53H82	Beta-lactamase-like protein 2 OS=Homo sapiens GN=LACTB2 PE=	26.53	2.78	1	0.685	1		0.494	1		0.722	1		288	32.8	6.80
P53004	Biliverdin reductase A OS=Homo sapiens GN=BLVRA PE=1 SV=2 -	26.51	7.09	1	0.692	1		0.881	1		1.274	1		296	33.4	6.44
P49773	Histidine triad nucleotide-binding protein 1 OS=Homo sapiens GN=	26.48	28.57	3	0.918	8	7.9	1.134	8	13.2	1.183	8	6.5	126	13.8	6.95
P31939	Bifunctional purine biosynthesis protein PURH OS=Homo sapiens (	26.44	7.26	2	0.754	3	12.4	0.881	3	22.1	1.168	3	7.3	592	64.6	6.71
P21926	CD9 antigen OS=Homo sapiens GN=CD9 PE=1 SV=4 - [CD9_HUM	26.19	21.49	4	1.135	9	9.5	0.851	9	7.1	0.749	9	5.8	228	25.4	7.15
P62081	40S ribosomal protein S7 OS=Homo sapiens GN=RPS7 PE=1 SV=	26.10	4.64	1	0.600	3	4.8	1.088	3	1.4	1.832	3	3.3	194	22.1	10.10
P61923	Coatomer subunit zeta-1 OS=Homo sapiens GN=COPZ1 PE=1 SV=	26.00	18.64	1	1.030	2	11.1	1.374	2	129.2	1.335	2	108.1	177	20.2	4.81
Q15691	Microtubule-associated protein RP/EB family member 1 OS=Homo	25.84	7.09	2	0.777	3	4.6	1.071	3	8.7	1.402	3	2.2	268	30.0	5.14
Q92734	Protein TFG OS=Homo sapiens GN=TFG PE=1 SV=2 - [TFG_HUM/	25.77	6.75	2	1.063	3	0.5	1.318	3	3.3	1.236	3	3.8	400	43.4	5.10
O00754	Lysosomal alpha-mannosidase OS=Homo sapiens GN=MAN2B1 PE	25.74	0.79	1	1.353	1		1.324	1		0.978	1		1011	113.7	7.28
Q9Y3D6	Mitochondrial fission 1 protein OS=Homo sapiens GN=FIS1 PE=1 S	25.57	7.24	1	0.905	5	6.8	0.851	5	8.7	0.984	5	3.2	152	16.9	8.79
Q9NWH9	SAFB-like transcription modulator OS=Homo sapiens GN=SLTM PE	25.37	0.87	1	1.230	3	0.3	1.462	3	1.6	1.186	3	6.1	1034	117.1	7.87
O00487	26S proteasome non-ATPase regulatory subunit 14 OS=Homo sapi	25.28	7.10	1	1.007	1		1.213	1		1.205	1		310	34.6	6.52
Q01085	Nucleolysin TIAR OS=Homo sapiens GN=TIAL1 PE=1 SV=1 - [TIA	25.27	4.80	1	0.806	2	0.8	0.888	2	13.3	1.101	2	12.6	375	41.6	7.74
P04040	Catalase OS=Homo sapiens GN=CAT PE=1 SV=3 - [CATA_HUMAN	25.22	6.07	2	1.463	3	16.2	1.807	3	10.6	1.190	3	5.5	527	59.7	7.39
P36873	Serine/threonine-protein phosphatase PP1-gamma catalytic subuni	25.05	8.36	2	0.829	3	7.0	0.629	3	5.1	0.769	3	1.9	323	37.0	6.54
P40222	Alpha-taxilin OS=Homo sapiens GN=TXLNA PE=1 SV=3 - [TXLNA_	24.96	2.56	1	1.036	1		1.353	1		1.305	1		546	61.9	6.52
O95777	U6 snRNA-associated Sm-like protein LSm8 OS=Homo sapiens GN	24.74	52.08	3	0.830	3	3.0	1.061	3	13.5	1.278	3	4.9	96	10.4	4.48
P26640	Valine--tRNA ligase OS=Homo sapiens GN=VARS PE=1 SV=4 - [SY	24.73	2.06	1	1.286	1		1.024	1		0.796	1		1264	140.4	7.59
P63244	Guanine nucleotide-binding protein subunit beta-2-like 1 OS=Hom	24.62	6.31	1	0.846	1		0.855	1		1.010	1		317	35.1	7.69
P13804	Electron transfer flavoprotein subunit alpha, mitochondrial OS=Hol	24.58	9.01	2	0.758	1		0.878	1		1.159	1		333	35.1	8.38
O94760	N(G),N(G)-dimethylarginine dimethylaminohydrolase 1 OS=Homo	24.29	7.37	2	0.846	4	29.9	1.154	4	30.3	1.253	4	17.0	285	31.1	5.81
P43363	Melanoma-associated antigen 10 OS=Homo sapiens GN=MAGEA1(	24.28	7.05	3	0.544	4	7.9	2.002	4	10.0	3.680	4	18.0	369	40.8	4.40
P59998	Actin-related protein 2/3 complex subunit 4 OS=Homo sapiens GN	24.21	23.21	2	1.332	6	10.9	1.090	6	12.8	0.830	6	10.2	168	19.7	8.43
Q15942	Zyxin OS=Homo sapiens GN=ZYX PE=1 SV=1 - [ZYX_HUMAN]	24.01	3.50	1	0.800	2	21.0	1.261	2	3.8	1.577	2	24.9	572	61.2	6.67
O75348	V-type proton ATPase subunit G 1 OS=Homo sapiens GN=ATP6V1	23.97	6.78	1	0.867	2	5.9	1.279	2	6.5	1.475	2	0.6	118	13.7	8.79
Q14566	DNA replication licensing factor MCM6 OS=Homo sapiens GN=MCI	23.88	2.07	1	0.883	1		1.129	1		1.279	1		821	92.8	5.41

O95373	Importin-7 OS=Homo sapiens GN=IPO7 PE=1 SV=1 - [IPO7_HUM	23.85	2.12	2	1.022	3	13.8	0.864	3	44.7	0.940	3	27.5	1038	119.4	4.82
Q9NUJ1	Mycophenolic acid acyl-glucuronide esterase, mitochondrial OS=H	23.45	5.23	1	0.925	2	2.0	0.953	2	14.9	1.030	2	16.9	306	33.9	8.57
Q9BUJ2	Heterogeneous nuclear ribonucleoprotein U-like protein 1 OS=Hon	23.36	2.57	1	1.052	1		0.882	1		0.838	1		856	95.7	6.92
P34096	Ribonuclease 4 OS=Homo sapiens GN=RNASE4 PE=1 SV=3 - [RN	23.25	8.84	1	1.050	1		**1.915**	1		**1.825**	1		147	16.8	9.03
Q9BRF8	Serine/threonine-protein phosphatase CPPED1 OS=Homo sapiens	23.14	5.41	1	0.848	2	4.6	1.373	2	12.9	**1.620**	2	8.3	314	35.5	6.20
P15170	Eukaryotic peptide chain release factor GTP-binding subunit ERF3A	22.83	2.61	2	0.848	2	1.8	0.898	2	2.0	1.059	2	3.8	499	55.7	5.62
Q99460	26S proteasome non-ATPase regulatory subunit 1 OS=Homo sapie	22.82	4.62	3	0.891	3	1.1	0.734	3	11.4	0.857	3	13.0	953	105.8	5.39
O94826	Mitochondrial import receptor subunit TOM70 OS=Homo sapiens G	22.82	2.96	1	0.952	1		0.835	1		0.877	1		608	67.4	7.12
Q7Z6K5	Arpin OS=Homo sapiens GN=ARPIN PE=1 SV=1 - [ARPIN_HUMAN	22.82	3.98	1	0.916	1		1.115	1		1.217	1		226	24.9	5.83
Q13510	Acid ceramidase OS=Homo sapiens GN=ASAH1 PE=1 SV=5 - [AS	22.82	7.34	2	1.056	2	10.2	1.304	2	6.1	1.236	2	16.4	395	44.6	7.62
Q9H0W9	Ester hydrolase C11orf54 OS=Homo sapiens GN=C11orf54 PE=1 S	22.79	2.54	1	0.804	2	5.7	0.867	2	25.1	1.077	2	19.2	315	35.1	6.70
P41091	Eukaryotic translation initiation factor 2 subunit 3 OS=Homo sapie	22.68	2.97	2	1.016	2	6.1	1.359	2	1.0	1.338	2	5.0	472	51.1	8.40
Q92922	SWI/SNF complex subunit SMARCC1 OS=Homo sapiens GN=SMAR	22.60	1.72	1	0.793	1		1.006	1		1.268	1		1105	122.8	5.76
P51665	26S proteasome non-ATPase regulatory subunit 7 OS=Homo sapie	22.47	4.63	1	0.901	1		0.963	1		1.070	1		324	37.0	6.77
O15042	U2 snRNP-associated SURP motif-containing protein OS=Homo sa	22.36	3.98	3	0.874	4	17.6	0.920	4	0.9	1.046	4	20.7	1029	118.2	8.47
P01130	Low-density lipoprotein receptor OS=Homo sapiens GN=LDLR PE=	22.36	1.86	1	0.868	1		0.951	1		1.096	1		860	95.3	5.05
P62993	Growth factor receptor-bound protein 2 OS=Homo sapiens GN=GF	22.34	5.53	1	0.865	1		1.050	1		1.215	1		217	25.2	6.32
P02786	Transferrin receptor protein 1 OS=Homo sapiens GN=TFRC PE=1	22.26	1.71	1	1.483	2	14.1	0.777	2	22.7	**0.524**	2	8.4	760	84.8	6.61
O75083	WD repeat-containing protein 1 OS=Homo sapiens GN=WDR1 PE=	22.25	5.12	4	0.993	5	16.2	1.019	5	14.8	1.107	5	10.2	606	66.2	6.65
P28072	Proteasome subunit beta type-6 OS=Homo sapiens GN=PSMB6 PE	22.18	28.87	4	0.691	4	3.9	0.868	4	11.2	1.168	4	11.4	239	25.3	4.92
Q8N684	Cleavage and polyadenylation specificity factor subunit 7 OS=Hom	22.17	5.31	1	0.670	1		0.702	1		1.048	1		471	52.0	8.00
Q9UL25	Ras-related protein Rab-21 OS=Homo sapiens GN=RAB21 PE=1 S	22.14	4.44	1	0.864	1		1.096	1		1.269	1		225	24.3	7.94
Q8TC20	Cancer-associated gene 1 protein OS=Homo sapiens GN=CAGE1 P	22.11	1.80	1	1.236	3	0.7	1.117	3	14.6	0.843	3	10.2	777	90.2	5.29
Q15019	Septin-2 OS=Homo sapiens GN=SEPT2 PE=1 SV=1 - [SEPT2_HUM	22.08	4.71	2	0.850	2	0.7	0.989	2	21.6	1.164	2	22.3	361	41.5	6.60
O14744	Protein arginine N-methyltransferase 5 OS=Homo sapiens GN=PRI	21.97	1.26	1	0.762	5	24.8	**0.556**	5	2.6	0.758	5	15.2	637	72.6	6.29
Q96JB5	CDK5 regulatory subunit-associated protein 3 OS=Homo sapiens G	21.86	3.36	1	0.937	1		0.733	1		0.782	1		506	56.9	4.75
P18077	60S ribosomal protein L35a OS=Homo sapiens GN=RPL35A PE=1	21.86	8.18	1	1.033	1		1.059	1		1.026	1		110	12.5	11.06
P51572	B-cell receptor-associated protein 31 OS=Homo sapiens GN=BCAP	21.85	2.85	1	0.949	4	2.5	0.873	4	1.7	0.934	4	9.5	246	28.0	8.44
Q5T9S5	Coiled-coil domain-containing protein 18 OS=Homo sapiens GN=C	21.77	1.03	2	0.699	1		0.749	1		1.072	1		1454	168.9	5.66
P06730	Eukaryotic translation initiation factor 4E OS=Homo sapiens GN=E	21.71	3.69	1	0.701	3	17.0	0.797	3	4.2	1.171	3	8.8	217	25.1	6.15
Q9UK45	U6 snRNA-associated Sm-like protein LSm7 OS=Homo sapiens GN	21.66	24.27	2	0.903	2	2.0	1.029	2	7.5	1.140	2	9.4	103	11.6	5.27
O00764	Pyridoxal kinase OS=Homo sapiens GN=PDXK PE=1 SV=1 - [PDXK	21.61	6.73	2	0.770	2	15.4	1.140	2	12.1	1.481	2	3.2	312	35.1	6.13
Q9UIJ7	GTP:AMP phosphotransferase AK3, mitochondrial OS=Homo sapie	21.19	11.01	2	0.998	3	34.6	1.232	3	19.5	1.353	3	0.7	227	25.5	9.16
O00273	DNA fragmentation factor subunit alpha OS=Homo sapiens GN=DF	21.08	6.95	2	0.822	2	5.6	**1.658**	2	4.3	**2.016**	2	9.8	331	36.5	4.79
P78417	Glutathione S-transferase omega-1 OS=Homo sapiens GN=GSTO1	21.02	14.94	2	0.902	2	30.5	0.902	2	17.3	1.001	2	12.7	241	27.5	6.60
Q8N8S7	Protein enabled homolog OS=Homo sapiens GN=ENAH PE=1 SV=	20.77	2.88	2	1.267	4	9.9	1.389	4	2.4	1.169	4	15.8	591	66.5	6.93
P11047	Laminin subunit gamma-1 OS=Homo sapiens GN=LAMC1 PE=1 SV	20.73	2.80	3	0.960	4	10.9	1.192	4	17.0	1.310	4	11.6	1609	177.5	5.12
P08865	40S ribosomal protein SA OS=Homo sapiens GN=RPSA PE=1 SV=	20.70	5.76	2	0.974	1		0.831	1		0.853	1		295	32.8	4.87
Q86SQ4	G-protein coupled receptor 126 OS=Homo sapiens GN=GPR126 P	20.59	1.47	1	**0.638**	1		0.747	1		1.170	1		1221	136.6	7.87
P46778	60S ribosomal protein L21 OS=Homo sapiens GN=RPL21 PE=1 SV	20.52	8.13	2	1.052	4	9.6	**1.579**	4	15.7	**1.530**	4	7.3	160	18.6	10.49
Q96AG4	Leucine-rich repeat-containing protein 59 OS=Homo sapiens GN=L	20.28	7.82	2	0.887	3	4.7	0.931	3	16.6	1.012	3	11.1	307	34.9	9.57
P26196	Probable ATP-dependent RNA helicase DDX6 OS=Homo sapiens G	20.23	3.93	1	1.244	1		0.901	1		0.724	1		483	54.4	8.66
Q9HA64	Ketosamine-3-kinase OS=Homo sapiens GN=FN3KRP PE=1 SV=2	20.19	4.53	1	0.879	1		0.841	1		0.957	1		309	34.4	7.33
Q09028	Histone-binding protein RBBP4 OS=Homo sapiens GN=RBBP4 PE=	16.02	6.35	3	0.876	7	4.2	0.926	7	11.5	1.056	7	7.5	425	47.6	4.89
O15020	Spectrin beta chain, non-erythrocytic 2 OS=Homo sapiens GN=SP	13.29	0.92	1	0.930	1		1.074	1		1.154	1		2390	271.2	6.11
Q8WZ42	Titin OS=Homo sapiens GN=TTN PE=1 SV=4 - [TITIN_HUMAN]	0.00	0.17	8	1.025	1		**0.466**	1		**0.454**	1		34350	3813.7	6.35
Q9BXP5	Serrate RNA effector molecule homolog OS=Homo sapiens GN=SR	0.00	2.97	1	**0.616**	1		0.773	1		1.255	1		876	100.6	5.96
Q3KP66	Uncharacterized protein C1orf106 OS=Homo sapiens GN=C1orf10	0.00	0.90	1	0.827	1		0.938	1		1.134	1		663	72.9	9.31
O00193	Small acidic protein OS=Homo sapiens GN=SMAP PE=1 SV=1 - [S	0.00	7.10	2	0.738	5	11.5	0.981	5	10.0	1.240	5	13.4	183	20.3	4.72
O75436	Vacuolar protein sorting-associated protein 26A OS=Homo sapiens	0.00	1.83	1	1.325	1		1.265	1		0.955	1		327	38.1	6.57
P62750	60S ribosomal protein L23a OS=Homo sapiens GN=RPL23A PE=1	0.00	4.49	1	0.881	1		1.410	1		**1.601**	1		156	17.7	10.45
Q9UNF0	Protein kinase C and casein kinase substrate in neurons protein 2 (	0.00	2.26	2	1.163	1		**1.765**	1		**1.518**	1		486	55.7	5.20
Q9Y592	Centrosomal protein of 83 kDa OS=Homo sapiens GN=CEP83 PE=	0.00	1.73	2										693	82.0	6.28
